# Softening the steps to gigantism in sauropod dinosaurs through the evolution of a pedal pad

**DOI:** 10.1126/sciadv.abm8280

**Published:** 2022-08-10

**Authors:** Andréas Jannel, Steven W. Salisbury, Olga Panagiotopoulou

**Affiliations:** ^1^ School of Biological Sciences, The University of Queensland, Brisbane, QLD 4072, Australia.; ^2^ Monash Biomedicine Discovery Institute, Department of Anatomy and Developmental Biology, Monash University, Clayton, VIC 3800, Australia.

## Abstract

How sauropod dinosaurs were able to withstand the forces associated with their immense size represents one of the most challenging biomechanical scenarios in the evolution of terrestrial tetrapods, but also one lacking robust biomechanical testing. Here, we use finite element analyses to quantify the biomechanical effects of foot skeletal postures with and without the presence of a soft tissue pad in sauropodomorphs. We find that none of the models can maintain bone stresses that fall within optimal bone safety factors in the absence of a soft tissue pad. Our findings suggest that a soft tissue pad in sauropods would have reduced bone stresses by combining the mechanical advantages of a functionally plantigrade foot with the plesiomorphic skeletally digitigrade saurischian condition. The acquisition of a developed soft tissue pad by the Late Triassic–Early Jurassic may represent one of the key adaptations for the evolution of gigantism that has become emblematic of these dinosaurs.

## Teaser

Biomechanical evidence attests that sauropod dinosaurs tiptoed around on cushioned heels to support their immense weight.

## INTRODUCTION

Sauropods, among the most iconic groups of dinosaurs, were originally believed to be semiaquatic animals that supported their gigantic size via water buoyancy (*1*, *2*). However, the discovery of the first sauropod tracks in terrestrial deposits in the mid-20th century (*3*), coupled with taphonomic survey (*4*), revealed their impressive land-dwelling abilities. Yet, despite extensive research on sauropod palaeobiology and morphological specializations associated with their evolutionary trajectory toward their archetypal gigantism (*5*–*7*), one outstanding question persists: How did sauropods, and in particular their feet, support their gigantic body on terra firma?

For extant large-bodied terrestrial mammals (e.g., elephants and rhinoceroses), the acquisition of large body size occurred through both an increase in limb bone robustness and a shift to a more upright limb posture (among other changes) (*8*–*10*). Current research into sauropods agrees that a combination of plesiomorphic characters (e.g., nonmastication and ovipary) and evolutionary trends (e.g., small head, long neck, vertebral pneumatization, and columnar limbs) has led to their iconic gigantism (*5*, *11*–*18*). However, to date, most research has focused almost exclusively on the analysis of the axial skeleton and upper and middle long bone segments (skull, neck, stylopodium, zeugopodium, and tail) (*14*, *19*, *20*). Perhaps because of the scarcity of complete sauropod autopodia in the fossil record (*21*), research on the lower limb regions [specifically the manus (forefoot) and pes (hind foot)] has been minimal in comparison. Given the important role the autopodia play in supporting body weight and facilitating locomotion in the largest extant terrestrial mammals (e.g., the elephant) (*22*–*27*), it seems likely that the autopodia may also have played a pivotal role in locomotor performance and gigantism within the sauropod clade (*28*, *29*).

While many hypotheses have been proposed to explain how the feet of sauropods could bear their massive weight, a shift in autopodial posture (i.e., the anatomical configuration of the autopodial skeleton) and the development of an enlarged soft tissue pad beneath the skeletal elements of the pes have received the most attention (*16*, *28*, *30*). This is understandable, given the correlation between body size, autopodial postures, and autopodial pads that has been shown in a diverse suite of terrestrial tetrapods (*22*–*27*, *29*, *31*–*33*). For instance, as stem proboscid mammals progressively became larger through their evolutionary history, they acquired a more upright autopodial skeletal posture and developed an enlarged pad of adipose and fibrous connective tissue that dominates the central and caudal part of the autopodium (*22*, *34*). A similar trend has been proposed for sauropod (*29*) and ornithopod dinosaurs (*35*), but this form-function relationship has not been tested directly using biomechanical means. This scientific gap is most likely due to the lack of soft tissue preservation in fossil specimens and past difficulties associated with developing a computational biomechanical framework.

Similar to elephants (*22*, *23*), it has been suggested that a soft tissue pad in the pes of sauropods may have played an important role in reducing bone stresses and autopodial pressures during weight bearing and locomotion (*28*, *29*). Nevertheless, the presence of a soft tissue pad in the sauropod pes currently relies on indirect and equivocal lines of evidence. First, apart from inferences made from ichnology where a rounded heel impression on the caudal margin of many sauropod pedal tracks has been observed (*3*, *36*, *37*), soft tissue pads in sauropods are not preserved in the fossil record [although the absence of evidence is not necessarily evidence of their genuine absence because soft tissues are rarely preserved in the course of diagenesis (*38*)]. Second, sauropod pedal tracks commonly conform to what are interpreted as pronounced plantigrade impressions (i.e., complete contact of the pes with the substrate) (*28*, *39*). This unique pattern may thus be interpreted as either (i) a skeletally plantigrade pes (i.e., complete contact of the pedal bones with the substrate) or (ii) an upraised skeletal pedal morphotype that incorporates a soft tissue pad beneath the elevated bones (*29*, *30*, *40*). Third, the evolutionary history of sauropods diverges markedly from that of large extant mammals (with which the soft tissue pad is compared). Caution should therefore be taken when inferring the morphofunctionalities of a structure within a clade with no living descendants. Consequently, one unsettled question remains: Did the pes of sauropods incorporate a soft tissue pad, and can evidence for it be quantified? With this question in mind, our main objectives were first to investigate the effects of skeletal pedal postures on bone stress distribution in species of various body sizes within distinct sauropod lineages and subsequently to investigate the effects of a virtual soft tissue pad on postural morphotypes subjected to similar mechanical conditions.

**Fig. 1.**
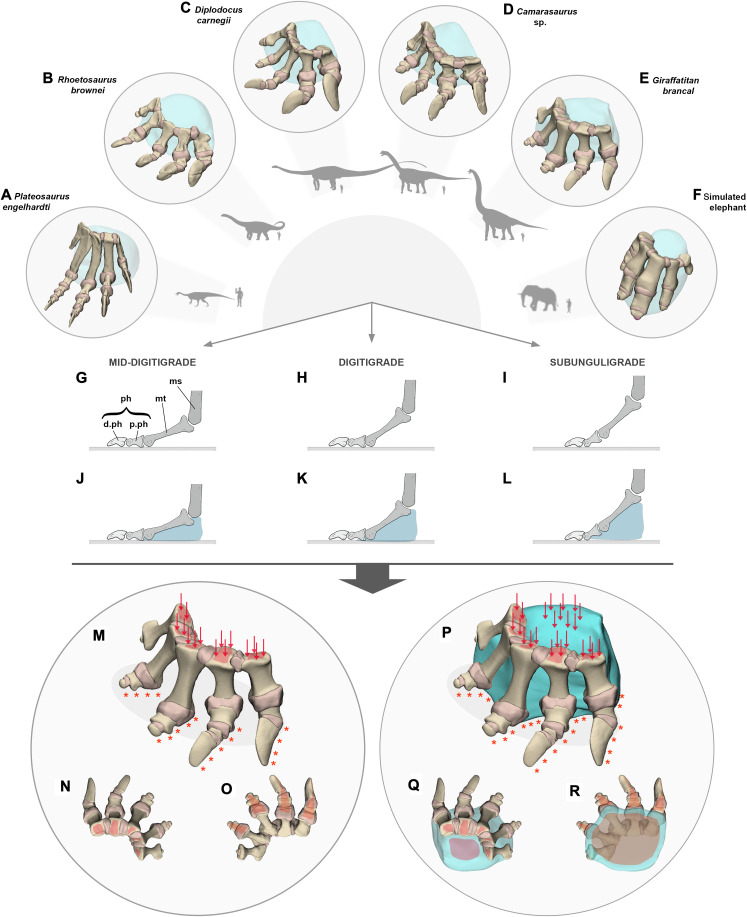
Schematic illustrations of the taxa and procedures used in this study. (**A** to **F**) Finite element models (FEMs) of (A) *Plateosaurus engelhardti*, (B) *Rhoetosaurus brownei*, (C) *Diplodocus carnegii*, (D) *Camarasaurus* sp., (E) *Giraffatitan brancai*, and (F) a simulated elephant pes. Schematic outlines of the animals illustrated next to each FEM. (**G** to **L**) Representations of the different postural morphotypes studied [where ms = mesopodium, mt = metapodium, and ph, p.ph, and d.ph = proximal and distal (generally the ungual) phalanges], including a mid-digitigrade posture (left), a digitigrade posture (center), and a subunguligrade posture (right). All postural morphotypes are compared between (G to I) pedal skeleton and (J to L) pedes with the presence of a hypothetical soft tissue pad. (**M** to **R**) Schematic representation of the digitigrade posture of *G. brancai* as an exemplar, including (M to O) the skeletal pes in (M) craniomedial view, (N) dorsal view, and (O) plantar view and (P to R) the pes with the presence of a hypothetical soft tissue pad in (P) craniomedial view, (Q) dorsal view, and (R) plantar view. These models replicate conditions in which the pedal FEMs would have been subjected to during the weight-bearing phase, where the dorsal surface of the pes is loaded vertically and downward (red arrows and red surfaces), and its plantar surface is constrained (orange asterisks and orange surfaces).

We hypothesized that sauropods had a soft tissue pad in their pes, which in turn functioned as a weight-bearing and bone stress reduction mechanism. To address these ideas, we conducted finite element analyses (FEA) to evaluate the biomechanical consequence of a wide range of pedal postures ranging from skeletally digitigrade to subunguligrade (i.e., with the heel elevated above the substrate) (*29*), which were subsequently supported by soft tissue pads of distinct sizes and material properties (Fig. 1). We performed sensitivity analyses to evaluate the effect of various unknowns surrounding fossils (e.g., skeletal pedal posture, soft tissue, material properties, and boundary conditions; Table 1). Last, we conducted validation studies by reproducing each analysis from a “blind anatomical perspective” using a simulated pes of an extant elephant (a phylogenetically distinct analog but the largest living land-dwelling vertebrate). This is the first time FEA has been used on a full multibody assembly of part of the postcranial skeleton (namely, the autopodium) of a fossil vertebrate, an approach easily applicable to other extinct taxa. Our data indicate that none of our models could have maintained pedal bone stresses that fall within optimal bone safety factors in the absence of a soft tissue pad.

**Table 1.** Details of the procedures and analyses undertaken in this study. BC, boundary conditions; C, cartilage property; *E*, Young’s modulus; F, soft tissue pad property; L, loading condition; SA, sensitivity analysis; ν, Poisson’s ratio (note: see fig. S1 for a simplified flowchart of the procedures).

**Assembly**		**Skeletal**	**Skeletal + Pad**	**References**
**Taxon**				
Outgroup		*Plateosaurus engelhardti*		Figs. 1 to 4; figs. S1 and S2
Sauropods		*Rhoetosaurus brownei*; *Diplodocus carnegii*; *Camarasaurus* sp.; *Giraffatitan brancai*		Figs. 1 to 4; figs. S1 and S2
Validation		Simulated elephant pes		Figs. 1 to 4; figs. S1 and S2
**Cartilage**		Cartilaginous capsule (20 mm)		Tables S1 to S5
**Postures**				
	Main study	Mid-digitigrade; Digitigrade; Subunguligrade		Figs. 1 to 4; Jannel *et al*. (*29*) (Fig. 5); figs. S6 to S11
	SA	Plantigrade; Unguligrade		Figs. S6 to S10
**Mesh density**		0.5 mm		Fig. S4; table S6
**Pad outlines**				
	Main study	N/A	Pad1	Fig. 2; figs. S3 and S6 to S10
	SA		Pad2	Fig. S12
			Pad3	Figs. S3, S34, S35, and S39
**Material properties**				
Bone		*E* = 10,000 MPa; ν = 0.3		Fig. 2; data S1
Cartilage	Main study	C1 (*E* = 100 MPa); ν = 0.4		Fig. 2; data S1
	SA	C2 (*E* = 10 MPa); C3 (*E* = 1 MPa); ν = 0.4		Figs. S13 to S18; data S1
Soft tissue pad	Main study	N/A	F4 (*E* = 100 MPa); ν = 0.49	Fig. 2; data S1
	SA		F1 (*E* = 0.1 MPa); F2 (*E* = 1 MPa); F3 (*E* = 10 MPa); ν = 0.49	Figs. S19 to S24; data S1
**Loads and constraints**				
Constraints	Main study	BC5	BC4′	Fig. 2; figs. S25 to S27
	SA	BC1; BC2; BC3; BC4; BC6	BC1′; BC2′; BC3′	Figs. S25 to S27
Loads	Main study	L2		Fig. 2; figs. S5 and S29 to S33; table S7
	SA	L1 (proxy)	L1 (proxy); L1B	Figs. S5 and S28; table S7
**Analyses**		Generalized linear models (*glm*)		Figs. S36 to S39; table S8

## RESULTS

**Fig. 2.**
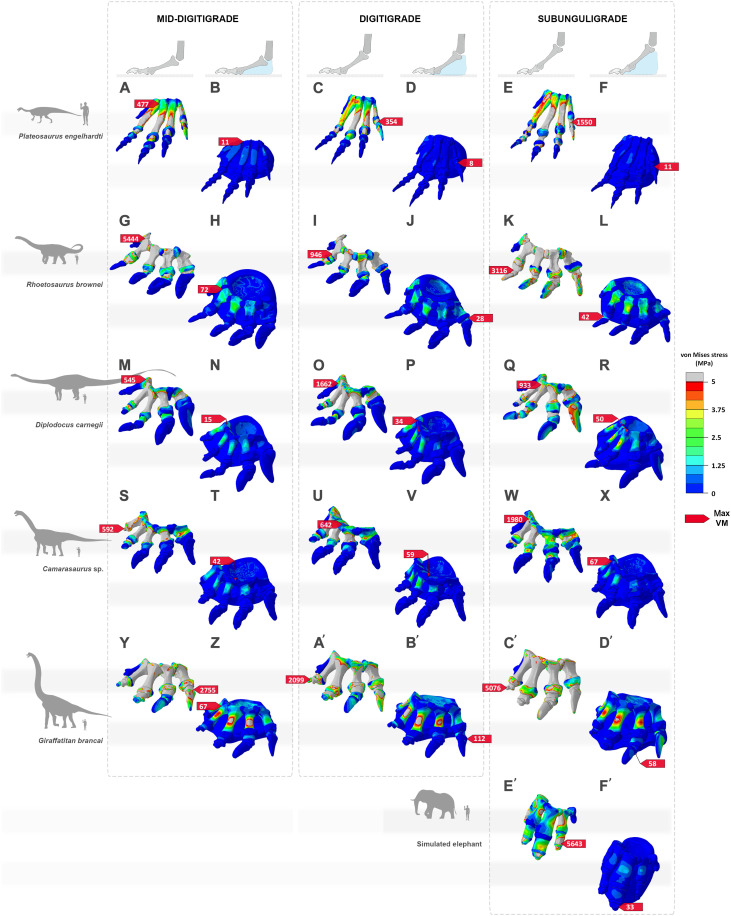
Von Mises stress (MPa) distribution results between distinct postural morphotypes of the pedal skeleton and pes with soft tissue pad of sauropodomorph specimens. These include (**A** to **F**) *P. engelhardti* in (A and B) a mid-digitigrade pedal morphotype, with (A) osteology alone and (B) a pes with a pad; (C and D) a digitigrade pedal morphotype, with (C) osteology alone and (D) a pes with a pad; (E and F) a subunguligrade pedal morphotype, with (E) osteology alone and (F) a pes with a pad; (**G** to **L**) *R. brownei* in (G and H) a mid-digitigrade pedal morphotype, with (G) osteology alone and (H) a pes with a pad; (I and J) a digitigrade pedal morphotype, with (I) osteology alone and (J) a pes with a pad; (K and L) a subunguligrade pedal morphotype, with (K) osteology alone and (L) a pes with a pad; (**M** to **R**) *D. carnegii* in (M and N) a mid-digitigrade pedal morphotype, with (M) osteology alone and (N) a pes with a pad; (O and P) a digitigrade pedal morphotype, with (O) osteology alone and (P) a pes with a pad; (Q and R) a subunguligrade pedal morphotype, with (Q) osteology alone and (R) a pes with a pad; (**S** to **X**) *Camarasaurus* sp. in (S and T) a mid-digitigrade pedal morphotype, with (S) osteology alone and (T) a pes with a pad; (U and V) a digitigrade pedal morphotype, with (U) osteology alone and (V) a pes with a pad; (W and X) a subunguligrade pedal morphotype, with (W) osteology alone and (X) a pes with a pad; (**Y** to **D′**) *G. brancai* in (Y and Z) a mid-digitigrade pedal morphotype, with (Y) osteology alone and (Z) a pes with a pad; (A′ and B′) a digitigrade pedal morphotype, with (A′) osteology alone and (B′) a pes with a pad; (C′ and D′) a subunguligrade pedal morphotype, with (C′) osteology alone and (D′) a pes with a pad; (**E′** and **F′**) a simulated elephant pes in a subunguligrade pedal morphotype, with (E′) osteology alone and (F′) a pes with a pad. Cold (blue) and warm (red) colors show lower and higher von Mises stresses, respectively.

When subjected to estimated lifelike physiological loads (*41*) (Fig. 2 and table S7), all sauropodomorph finite element models (FEMs) show an increased concentration of von Mises stresses in central and lateral pedal digits II to IV (with maximum von Mises stresses >> 500 MPa), with the highest stresses at the shaft of the metatarsals (Fig. 2 and figs. S6 to S10). Under similar conditions, the skeletal FEM of the simulated elephant pes without soft tissue pad shows a contrasting increase in concentration of von Mises stresses in central and medial digits II and III (with maximum von Mises stresses > 5000 MPa), with the highest stresses localized at the shafts of the metatarsals and in the phalanges (Fig. 2 and fig. S11).

**Fig. 3.**
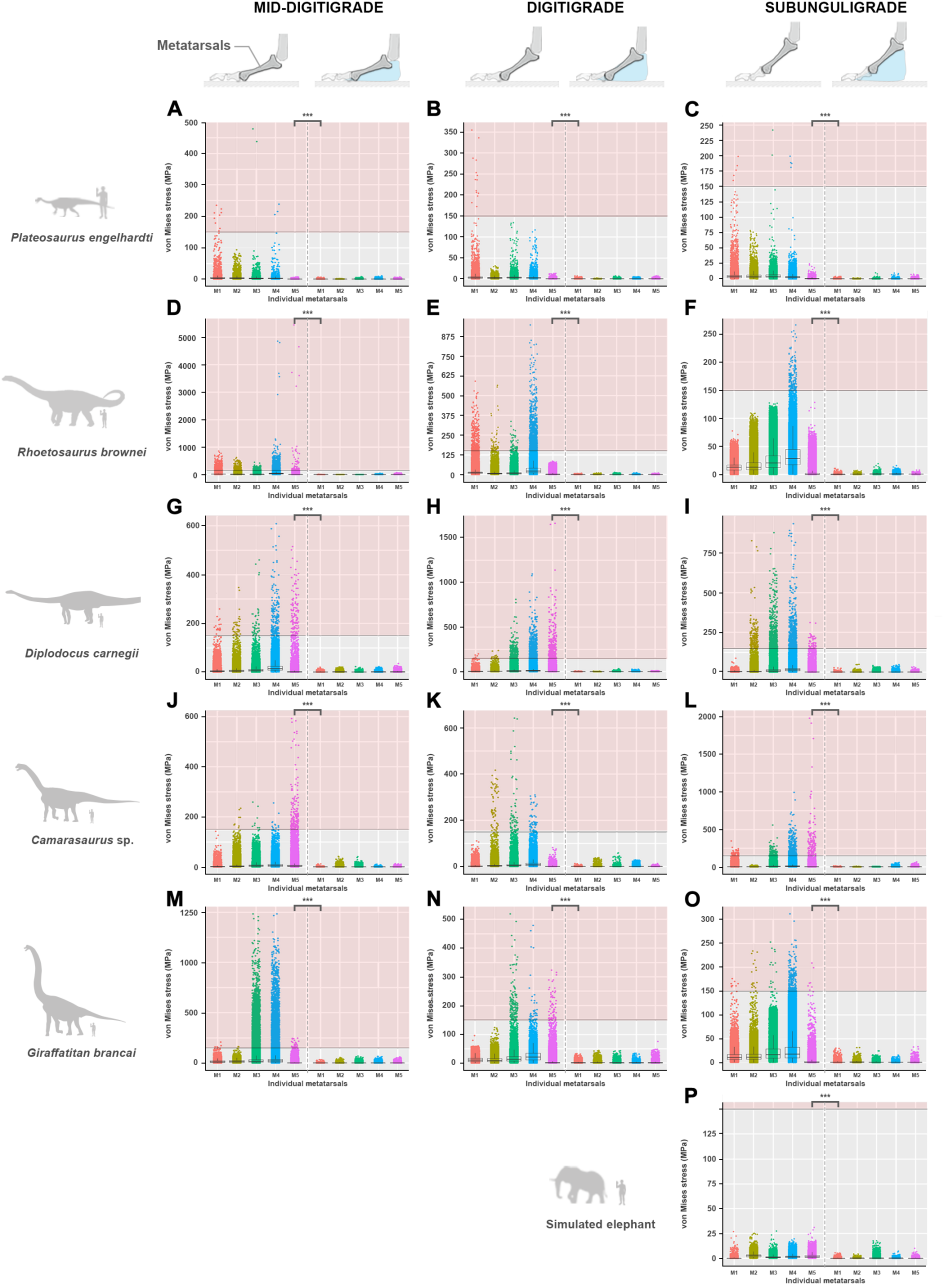
Comparisons in von Mises stress values (MPa) recorded on the nodes of each metatarsal for all pedal postural morphotypes between the skeletal FEMs with and without the presence of a hypothetical soft tissue pad for all studied specimens. These include (**A** to **C**) *P. engelhardti* in (A) a mid-digitigrade, (B) a digitigrade, and (C) a subunguligrade morphotypes; (**D** to **F**) *R. brownei* in (D) a mid-digitigrade, (E) a digitigrade, and (F) a subunguligrade morphotypes; (**G** to **I**) *D. carnegii* in (G) a mid-digitigrade, (H) a digitigrade, and (I) a subunguligrade morphotypes; (**J** to **L**) *Camarasaurus* sp. in (J) a mid-digitigrade, (K) a digitigrade, and (L) a subunguligrade morphotypes; and (**M** to **O**) *G. brancai* in (M) a mid-digitigrade, (N) a digitigrade, and (O) a subunguligrade morphotypes. (**P**) Simulated elephant pes in a subunguligrade morphotype. Individual data points (dot) represent singular von Mises stress value of a node for each metatarsal. Red backgrounds denote the theoretical limit of 150 MPa, representing the safety margin of a bone (following the value reported in the literature of what the bone of a human and bovid femur is capable of withstanding before physical damage). ***P < 0.001. Detailed statistical results provided in table S8. Note: The von Mises stress scale varies between graphics. This figure focuses on the metatarsals, but see fig. S36 for further details on the von Mises stress values recorded in the phalanges (particularly regarding the simulated elephant pes, which showed the clear difference in von Mises stresses between a pes with and without a soft tissue pad) and figs. S37 and S38 for the values of maximum and minimum principal stresses recorded in the metatarsals and the phalanges of each specimen, respectively.

Comparatively, the presence of a pedal soft tissue pad in all FEMs substantially reduces von Mises stresses for all postural morphotypes and each taxon compared with loading the skeletal postures alone (with maximum von Mises stresses < 100 MPa) (Figs. 2 and 3 and figs. S6 to S10). The results are supported by a statistically clear difference of von Mises stresses (*P* < 0.001) between each bone of the skeletal FEMs lacking a soft tissue pad and FEMs that had one, an outcome retained for all sensitivity analyses, as well as for maximum and minimum principal stresses (Fig. 3, figs. S36 to S38, and table S8). In all cases, the general spatial patterning of pedal bone stresses of the FEMs with a soft tissue pad resembles the one recorded in the corresponding skeletal FEMs that lacked a soft tissue pad.

The sensitivity analyses on the geometry and size of the soft tissue pad show that a pad extending from, and plantar to, the tarsus-crus complex to the most distal joints results in marginally higher concentrations of von Mises stresses compared to a pad impeded by the first joints in contact with the substrate (fig. S12). Dimensions aside, all soft tissue pad configurations substantially reduce bone stresses in all postural morphotypes compared with loading of the skeletal postures alone.

The sensitivity analyses on the material properties of the cartilaginous capsules and on the soft tissue pad result in similar spatial patterning of von Mises stresses for all FEMs of each taxon but differ in stress magnitude, respectively (figs. S13 to S23). Our analyses show that components with a lower Young’s modulus *E* value (<10 MPa) record marginally higher concentrations of von Mises stresses compared to higher *E* value (>10 MPa). Similar outcomes are observed in the simulated elephant pes (figs. S18 and S24).

The sensitivity analysis on the boundary conditions shows marginally similar spatial patterning of von Mises stresses for all postural morphotypes but differs substantially in stress magnitude (figs. S25 to S28). Constraining nodes in the plantar surfaces of the most distal elements of the pes results in higher degree of von Mises stress magnitudes compared to constraining nodes in the plantar surfaces of all the elements in contact with the ground (figs. S25 to S27). For a more detailed comparison of all results and sensitivity analyses, see the Supplementary Materials.

## DISCUSSION

Our results show that, irrespective of skeletal pedal posture (Fig. 1), all sauropodomorph specimens examined (i.e., representatives of distinct clades and diverse body sizes) would have been unable to support their weight without a soft tissue pad in the pes (Figs. 2 and 3). All skeletal morphotypes without a soft tissue pad resulted in maximum von Mises stresses higher than 500 MPa for all pedal models (up to 5000 MPa as recorded in *Rhoetosaurus brownei*; Fig. 3). As expected, bone stress increased principally in the shafts of each metatarsal and the most proximal phalanges (Figs. 2 and 3), likely due to the pedal posture, boundary conditions, and the fact that the metatarsals are the longest bones in the sauropod pes. Mechanically, it is highly unlikely that sauropod pedal bones could have withstood bone stresses of this magnitude without failing (*42*). This is because sauropod bones have been shown to retain the general structural properties of Haversian bone tissue seen in modern birds and large mammals (*5*, *43*), indicating that they were most likely subjected to comparable mechanical constraints. In humans and bovids, cortical bone (e.g., such as in the femur) has been evaluated to withstand maximum stress < 150 to 200 MPa (*44*, *45*). Hence, within the context of comparable loading regimes, the mechanical state of each sauropod model examined suggests that all skeletal pedal postures would most likely have resulted in mechanical failure (e.g., stress fractures). This state would have been intensified when subjected to repetitive heavy loadings, as would be expected during normal locomotion, ultimately resulting in fatigue fracture in all digits. Being unable to support or move properly, the high probability of mechanical failure would have had a substantial impact on the animal’s survival.

Our analysis provides quantitative numerical evidence that the presence of a soft tissue pad substantially reduces the overall stress-strain responses within the pedal bones of all sauropodomorph specimens examined (Figs. 2 and 3), an outcome recorded regardless of the relative dimensions (fig. S12) and material properties of the soft tissue pad itself (figs. S19 to S24), constraints (figs. S26 to S28), or applied forces (figs. S29 to S33). In all cases, the presence of a soft tissue pad beneath the central and caudal part of the pedal skeleton reduces von Mises stresses well under the limits of potential physical failure (with maximum von Mises stresses < 100 MPa) (Fig. 3). The marked reduction in bone stress recorded in our FEMs is reminiscent of the biomechanical aptitude of the cushioning pads of large extant mammals. In elephants, rhinoceroses, and camelids, padding structures have been suggested to absorb mechanical shock, store and release elastic energy, as well as to keep locomotor pressures low (*23*–*26*, *34*, *46*–*49*). This idea is consistent with the results of our simulated elephant pes (Figs. 2 and 3). Given this similarity in our FEMs, it is possible that a pedal soft tissue pad in sauropods also had the capacity to absorb and release energy. This energy could have been stored initially in the soft tissue pad before being released during the subsequent stance phase of locomotion.

While our results show that the presence of a soft tissue pad would have been imperative for the survival of each of the species we studied, the similarities in bone stress patterns among different postural configurations reinforces the idea that a functional autopodium may encompass a range of subtly different postures (*29*) (an outcome supported by the joint movements observed during loading; see movies S1 to S6). Moreover, the uniform accumulation of bone stresses recorded on the lateral digits of each sauropod FEM could have been damaging when subjected to repetitive loadings, thus likely reducing the margin of safety of the pedal bone assembly. This vulnerable mechanical state may correlate with some pathological cases reported within the bones of sauropod autopodia (such as fracture, enthesophyte, and osteoarthritis) (*50*, *51*). Analogous foot pathologies have been observed among living elephants and rhinoceroses (*23*, *24*, *52*–*54*). However, considering their immense size, it is unlikely that sauropods would have progressed through life with a continuously high probability for chronic injuries. Hence, we hypothesize that additional extrinsic soft tissues (e.g., muscles, tendons, ligaments, and sole skin) and decreased forces throughout kinematics (such as the potential for absorbing loads at heel strike and/or distributing forces evenly between the limbs) would have also played an important role in reducing the stress applied to the pedal bones. In addition, given that our FEMs on a simulated elephant pes record higher stress-strains responses in the central and medial pedal digits, but given that extant elephants have been shown to exhibit higher concentration of peak pressures laterally (*23*), it could mean that elephants transfer most of the locomotor forces laterally to reduce stress in the most vulnerable medial region of their pedes. Therefore, a similar, yet opposite, scheme may be extrapolated in our sauropod models. Given our results, it is possible that the taxa analyzed, and presumably sauropods in general, might have used the medial portion of the pes to counter most of the loading forces, thus reducing stresses on the more vulnerable lateral region of the pes. Although this hypothesis necessitates further in vivo and mechanical investigations, the idea is corroborated by the pedal morphology of sauropods and is further supported by the ichnological record. First, sauropod medial digits I and II are known to be more robust and often considered as the weight-bearing digits (*15*), an adaptation conventionally linked to the acquisition of larger body size (*8*–*10*). Second, the craniomedial margin of well-preserved sauropod pedal tracks often appears more deeply impressed than the lateral one (*37*). While this pattern does not necessarily imply an increase in applied forces medially (*55*), it does suggest that the medial portion of the pes likely played the leading weight-bearing role during locomotion.

**Fig. 4.**
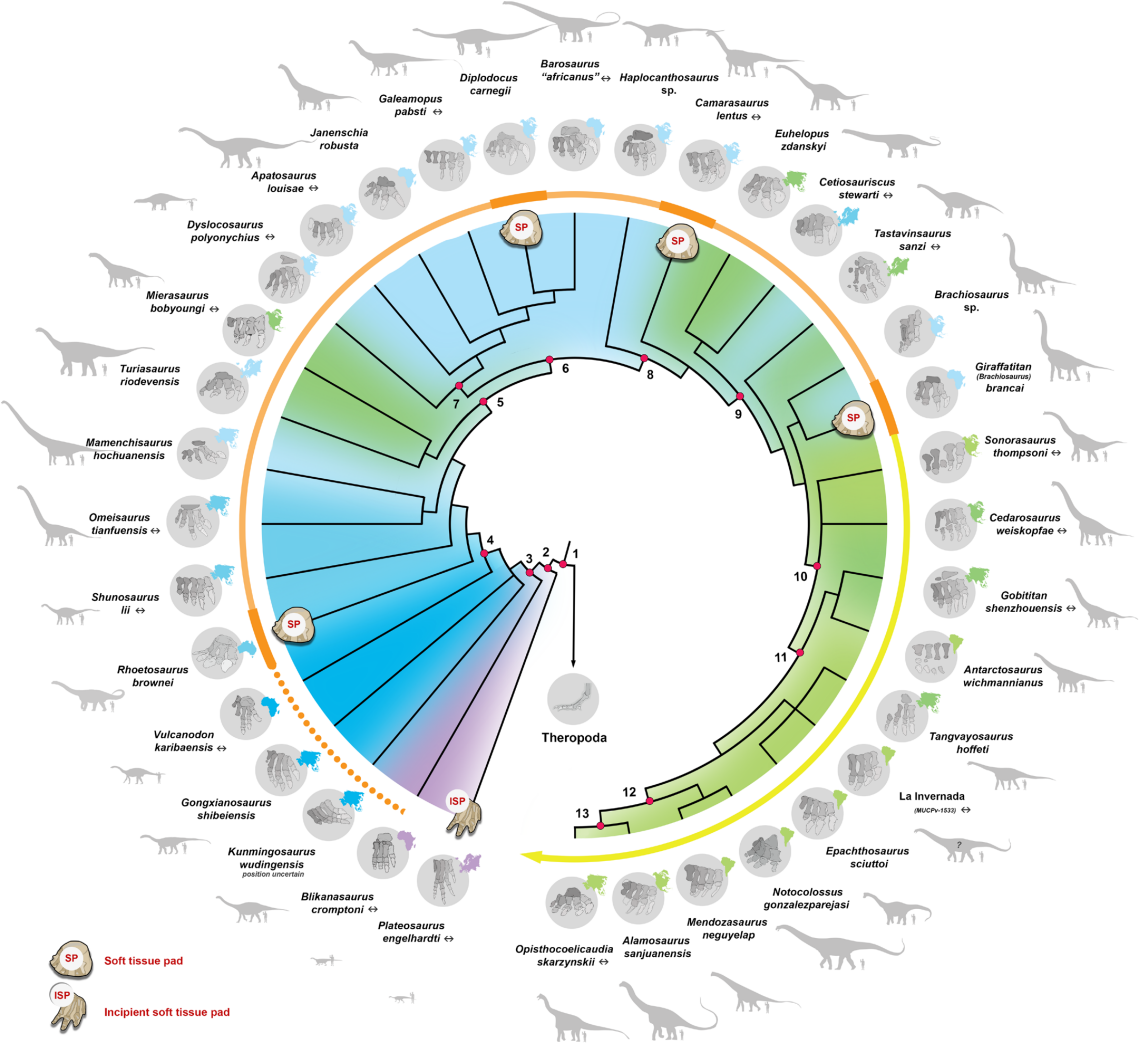
Simplified phylogeny of sauropod taxa with the most complete pedes. For each taxon, drawings illustrate (i) articulated (or closely associated) right pes in dorsal view (↔ indicate mirrored left pes), (ii) outline of their respective spatiotemporal localities in the top right corner of each ellipse (conforming with the chronostratigraphic color coding), and (iii) reconstructed outlines of each specimen put to scale compared to a human outline. The colored gradient arrow represents the current qualitative and quantitative evidence for the presence of a pedal soft tissue pad within the clade, which is subdivided in distinct colors: (i) the initial orange dotted line denotes the proposed acquisition of a caudally developed pedal pad sometimes by the Late Triassic–Early Jurassic times; (ii) the orange solid line represents the quantified (thick orange lines) and the extrapolated (light orange lines) presence of a developed soft tissue pedal pad in our study specimens and all the other taxa included in between, respectively; (iii) the yellow line denote the extrapolated retention of the neomorphic pedal soft tissue pad within all derived lineages. Simplified soft tissue pad outlines illustrated for each of our specimen examined, including an incipient soft tissue pad (ISP) and a soft tissue pad (SP). Numbers indicate major clades, including 1, Saurischia; 2, Sauropodomorpha; 3, Sauropoda; 4, Gravisauria; 5, Eusauropoda; 6, Neosauropoda; 7, Diplocoidea; 8, Macronaria; 9, Titanosauriformes; 10, Somphospondyli; 11, Titanosauria; 12, Eutitanosauria; 13, Lithostratia. Source of adapted drawing and notes are listed in table S9 and data S2.

**Fig. 5.**
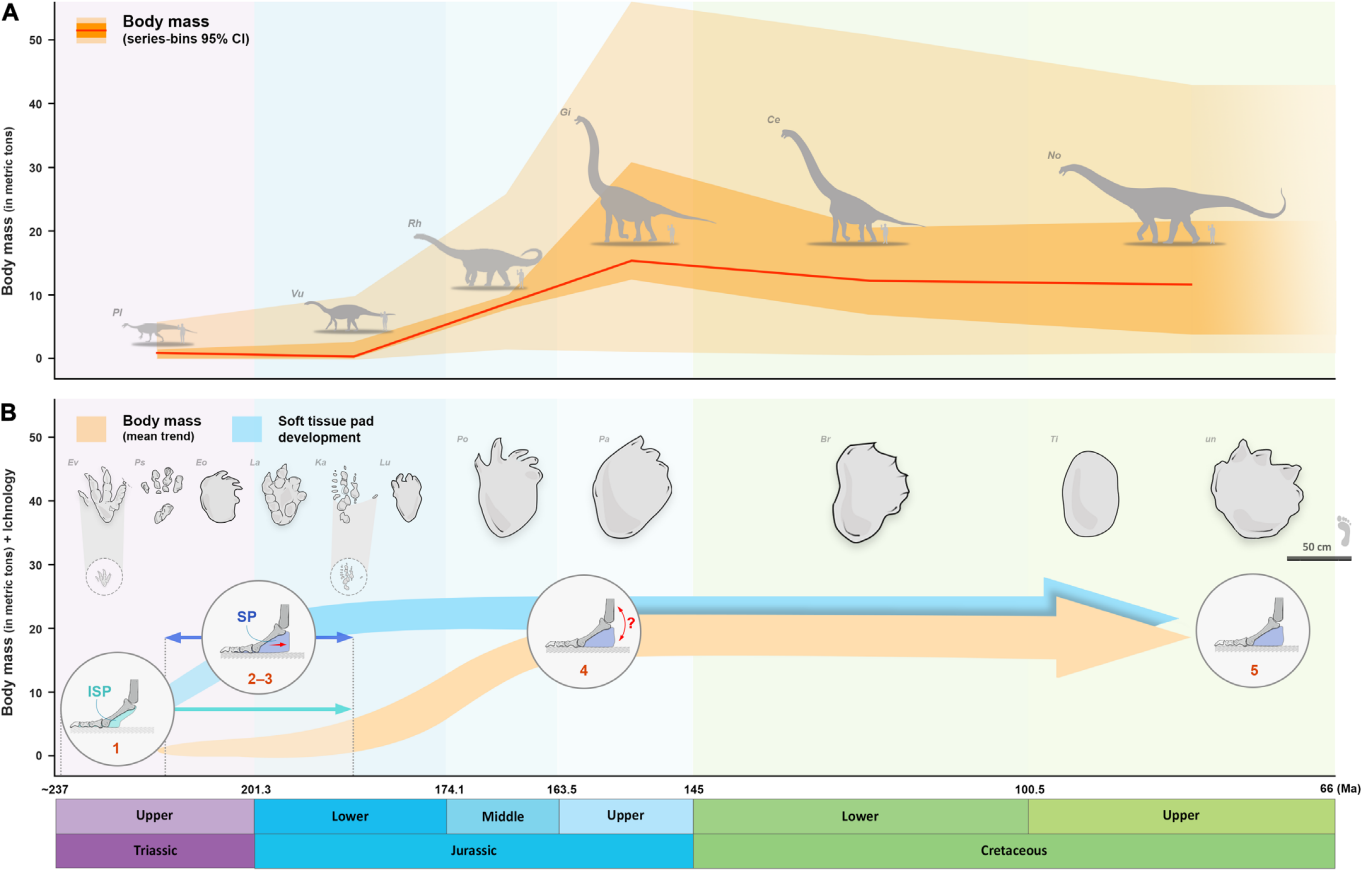
Main evolutionary steps proposed for the morphofunctional and postural changes of the sauropod pedes. (**A**) Sauropod body mass through time (in metric tons) based on the sauropod body mass estimations of (*41*) (NB: data lacking for the second half of the Upper Cretaceous so illustrated here faded, in continuity with the data recorded in the Cretaceous). Schematic outlines of selected large specimens illustrated in the curve, including (from left to right) *P. engelhardti*, *Vulcanodon karibaensis*, *R. brownei*, *G. brancai*, *Cedarosaurus weiskopfae*, and *Notocolossus gonzalezparejasi*. (**B**) Projected evolutionary changes occurring in the sauropod pes associated with trend in body mass, including 1, skeletal and functional digitigrade pedal posture among basal non-sauropod sauropodomorphs with an incipient soft tissue pad (ISP) (see figs. S34 and S35); 2 and 3, expansion of a well-developed soft tissue pad beneath the elevated pedal bones (SP), resulting in a functionally plantigrade pes + retention of skeletal posture within a range of digitigrady; 4, retention of a soft tissue pad and yet undetermined trend toward more elevated bones; 5, conservation of the neomorphic soft tissue pad within all lineages. Selected examples of well-preserved non-sauropod sauropodomorph and sauropod pedal tracks illustrated above the trends, including (from left to right) *Evazoum siriguii*; *Pseudotetrasauropus bipedoida*, *Eosauropus isp.*, *Lavinipes cheminii*; *Kalosauropus pollex*, *Liujianpus shunan, Polyonyx gomesi*; *Parabrontopodus mcintoshi*; *Brontopodus birdi*; *Titanopodus mendozensis*; and unnamed Asian sauropod track. Source of adapted drawing and notes are listed in table S9 and data S2.

Our survey of the fossil record allows us to partially assess how sauropod pedal posture and function have evolved (Figs. 4 and 5). Basal non-sauropod sauropodomorphs, such as *Panphagia protos* (*56*), were small and gracile animals, with an estimated mass of <150 kg (*41*). More derived forms, such as *P. engelhardti*, were considerably larger, with an estimated mass of <1 metric tons. These sauropod precursors are conventionally reconstructed as facultative bipeds with a digitigrade pedal posture (i.e., standing on the toes and not touching the ground with the heel)—the characteristic stance of all basal saurischians (*16*, *57*). On the basis of our results, the pedal skeleton of *P. engelhardti* would be insufficient to brace the pes against the mechanical loads associated with bipedal weight bearing without the additional support of an incipient soft tissue pad, although this model resulted in the lowest von Mises stresses compared to all other FEMs (Figs. 2 and 3). Tracks that are typically attributed to the earliest diverging sauropodomorphs usually indicate a tetradactyl pes with four distally directed digital impressions (Fig. 5) (*58*). Ichnotaxa such as *Evazoum* (*59*, *60*) and *Otozoum* (*61*) show evidence of an enlarged, sometimes coalesced, metatarsophalangeal pad proximal to the impressions of digits III and IV, and, farther proximally, what could be regarded as an incipient “heel” pad (Fig. 5). This proximally positioned heel pad impression is also evident in other Upper Triassic and Lower Jurassic ichnotaxa such as *Kalosauropus* and *Pseudotetrasauropus*, both of which have been linked to massospondylid (*62*) and *Plateosaurus*-like (*63*) non-sauropod sauropodomorphs, respectively. It seems that the likely presence of an incipient soft tissue pad in non-sauropod sauropodomorphs could have provided the foundation for the gradual acquisition of a more substantial structure (i.e., a caudal expansion of the pad and increased continuity between the phalangeal pads of each digit) to effectively reduce bone stresses in the pes as sauropods increased in size (*29*). Indeed, the earliest true sauropods [such as *Tazoudasaurus naimi* or *Vulcanodon karibaensis* (*64*, *65*)] and their closest relatives (*66*, *67*) have been proposed to display more graviportal stances [with estimated mass around 10 metric tons (*5*, *41*, *66*, *67*)]. Unfortunately, these taxa lack sufficiently well-preserved pedal skeletons (Fig. 4), which might have otherwise provided evidence on early morphological changes. Some contemporaneous pedal tracks (i.e., from Upper Triassic–Lower Jurassic strata), depicting clear, rounded caudal margins, have been tentatively attributed to sauropods or their immediate predecessors (Fig. 5) (*68*, *69*). This pattern is in accordance with the development of a large pedal soft tissue pad structure (*3*, *36*, *37*, *70*). If tracks assigned to some of these ichnotaxa do indeed pertain to early sauropod trackmakers, it could push back the origin of the clade Sauropoda to the Late Triassic, along with the acquisition of an apomorphic soft tissue pad. It cannot be excluded, however, that these trackmakers were non-sauropod sauropodiforms (*68*), suggesting a possible convergent adaptation of a large sauropod-like soft tissue pad within the clade Sauropodomorpha. Eventually, most sauropod lineages attained gigantic body sizes throughout the rest of the Mesozoic (*41*). Hence, given our results, it is unlikely that more derived taxa could have reached larger body mass without retaining the mechanical benefits of a soft tissue pedal pad (Figs. 4 and 5).

On the basis of our FEA results and the current state of the sauropodomorph fossil record, we propose that sauropod species likely acquired a pedal soft tissue pad early in the course of their evolution. Ultimately, this early acquisition modified their pedes into a functionally plantigrade morphotype while retaining a plesiomorphic skeletal posture close to a range of digitigrady to subunguligrady. This evolutionary model differs from the scenario proposed for Proboscidea, where the development of a cushion structure was suggested to occur through a transition of “flat-footed” to more derived “tip-toed” morphologies (*22*). The results also differ from the preferred evolutionary model proposed for ornithischian dinosaurs, where larger forms (e.g., hadrosaurs) have been proposed to present more upright skeletal pedal postures than basal ones (*35*). Because our study cannot attribute a specific skeletal postural morphotype to any of our specimens but rather reveal a range of possible ones, linking an evolutionary transition toward a more upright skeletal pedal posture in sauropods would be premature given these results. It remains unclear whether the conventional model in which larger animals adopt more upright posture (*9*) does not apply to sauropods or whether this traditional model may be more complex than previously thought. Nevertheless, these findings do indicate that distinct lineages of large terrestrial tetrapods evolved convergent soft tissue pad structures within their pedes. Hence, there is probably a biological link between the acquisition of gigantism and the benefits of having a soft tissue structure to absorb and redirect some loads away from the pedal skeleton.

Ultimately, the association of a digitigrade-to-subunguligrade skeletal posture and functionally plantigrade pes would have combined the mechanical advantages of both morphotypes. For instance, postural traits unique to sauropods could have included the presence of (i) a larger plantar surface area [i.e., larger transfer of pressure (*29*)], (ii) the long axis of bones being better aligned to the ground reaction force [i.e., allowing the support of higher loads (*9*, *10*, *29*, *35*, *71*)], (iii) the retention of some digital joint movements triggered by a range of skeletal pedal posture [i.e., acting as stress-reducing mechanisms (*29*)], and (iv) a recurved proximal articulation between the metatarsals [bringing the peripheral elements closer to the central axis of support (*29*, *72*)]. The development of an incipient soft tissue pad would have therefore provided the required mechanical advantages to withstand increasing forces. This apomorphy would in turn have allowed sauropod species to reach a larger body mass (>10 metric tons) early in their evolution, possibly by Late Triassic–Early Jurassic times [~230 to 174 million years (Ma) ago]. This apomorphy could therefore represent one of the key adaptations that helped sauropods achieve their emblematic gigantism. Our findings provide some of the first compelling pieces of evidence supporting the long-standing hypothesis for the presence of a soft tissue pad within the sauropod pes (*15*, *28*, *36*, *40*).

## MATERIALS AND METHODS

### Model construction

The nearly complete pedes of five sauropodomorphs representative of distinct clades and various body sizes (900 to 34,000 kg) based on fossils spanning the Upper Triassic to Upper Jurassic (~235 to 145 Ma ago; Fig. 1 and figs. S1 and S2) were subjected to photogrammetry using Agisoft Photoscan Professional v1.0.3 (*73*, *74*). We selected the Upper Triassic *Plateosaurus engelhardti* to represent a non-sauropod (out-group) exemplar. Sauropod pedes included the lower Upper Jurassic *R. brownei* and three Upper Jurassic taxa representing three distinct clades: *Diplodocus carnegii*, *Camarasaurus* sp., and *Giraffatitan brancai* (Table 1 and figs. S1 and S2). To validate our methods, we used the pes of an extant African elephant, which was virtually reconstructed using referenced image planes (fig. S2). The three-dimensional (3D) models of each taxon were imported into Autodesk Maya 2017 (www.autodesk.com), where the full set of virtual postural morphotypes and associated soft tissues was reconstructed (figs. S1 to S3). Choices of the range of postural morphotypes (i.e., mid-digitigrade, digitigrade, and subunguligrade) were selected on the basis of (*29*). Other postures (i.e., plantigrade and unguligrade) were also subjected to sensitivity analyses (Table 1 and figs. S6 to S10 and S29 to S33). Each joint was enclosed in a virtual cartilaginous capsule generated in Autodesk to replicate a synovial joint (Table 1 and tables S1 to S5 for cartilage estimations). Each 3D surface model was then converted into a volumetric mesh file of continuum linear tetrahedral elements of types C3D4 using 3-Matic 11.0 software (Materialize Inc., Leuven, Belgium). In addition, we performed a series of sensitivity analyses to evaluate the effect of mesh density on the model (fig. S4). Each mesh file was then imported into Abaqus/CAE 6.13-6.23 FEA software, preserving the coordinate systems of each segment as defined for each morphotype. Further details regarding the constructions of the models are available in the Supplementary Materials.

### Material properties

Because of the current lack of information on the material properties of anatomical constituents in fossils, linear elasticity, homogeneity, and isotropy were assumed for each model. Previous studies have suggested that bones of many dinosaurs (and other archosaurs) appear analogous to the Haversian bones of fast-growing bovine mammals (*75*–*77*). Thus, each bone was assigned a Young’s modulus (*E*) value of 10,000 MPa and a Poisson’s ratio (ν) of 0.3 for consistency (Table 1). For each virtual cartilaginous capsule, an *E* value of 100 MPa and a ν value of 0.4 were assigned, assuming that cartilage in sauropods would have presented similar universal cartilaginous properties to that of most other vertebrates, i.e., predominantly sharing elastic functions (*78*, *79*). A sensitivity analysis was conducted to assess the potential variations in cartilage properties on our models (with *E* ranging from 1 to 100 MPa; Table 1 and figs. S13 to S18). The soft tissue pad was modeled as nonlinear, viscoelastic tissue, somewhat analogous to cartilage, as interpreted for the padding tissues found in living terrestrial organisms (*47*, *80*). We conducted additional sensitivity analyses to assess some of the unknowns regarding the modeling of the soft tissue pad, including (i) variations in overall outlines (figs. S12, S34, and S35) and (ii) variations in material properties (with *E* ranging from 0.1 to 100 MPa; Table 1 and figs. S19 to S24). Each soft tissue pad presented here was assigned a proxy Young’s modulus value of 100 MPa and a Poisson’s ratio of 0.49 (Figs. 2 and 3 and Table 1).

### Loads and constraints

To simulate weight bearing of the autopodium in the 3D Cartesian coordinate system (i.e., complete contact of the autopodium with the substrate), the dorsal surface of the pes was loaded vertically in the inverse direction of the *z* axis (i.e., toward the substrate), and its plantar surface (assumed in interaction with the ground) was constrained (Fig. 1). Specifically, the applied load was distributed evenly on selected nodes on the proximal surfaces of each metatarsal. An initially applied force of 10,000 N was used as a proxy between our specimens (Table 1 and figs. S6 to S10), which corresponded to the loading value for a living elephant (*23*). More physiologically realistic loading values were then applied to each sauropodomorph model (figs. S29 to S33 and table S7). These loads were measured by multiplying the estimated body mass of each specimen (*m*, in kg) with the gravitational acceleration (i.e., *g* = 9.834 m/s^2^) to obtain a force measurement in newtons. The resulting force was divided by four under the assumption that force was equally distributed between the four autopodia during the support phase (i.e., *W* = *mg*; with *F* = $\frac{1}{4}$*W*). Thus, these more realistic applied forces ranged from 8000 to 85,000 N for *P. engelhardti* and *G. brancai*, respectively (Table 1 and table S7). Constraints were fixed from rigid body motion on selected nodes on the plantar surfaces of each element in assumed contact with the ground. A series of sensitivity analyses were conducted to assess variations in constrained nodes for both boundary and loading conditions (figs. S25 to S28). Strictly similar material properties and mechanical conditions were applied on the simulated elephant pes.

## Supplementary Materials

### This PDF file includes:

Figs. S1 to S39 Tables S1 to S9 References

## Notes

View/request a protocol for this paper from *Bio-protocol*.
