# Supplementary material for: Softening the steps to gigantism in sauropod dinosaurs through the evolution of a pedal pad
Source: Sci Adv. 2022 Aug 10;8(31):eabm8280. doi: 10.1126/sciadv.abm8280 (PMC9365286; doi:10.1126/sciadv.abm8280)
Supplement: Supplementary file 1 — Figs. S1 to S39 Tables S1 to S9 References [file sciadv.abm8280_sm.pdf]

Science Advances
AAAS

Supplementary Materials for

Softening the steps to gigantism in sauropod dinosaurs through the evolution of a pedal pad

Andréas Jannel *et al.*

Corresponding author: Andreas Jannel, andreas.jannel@gmail.com, andreas.jannel@mfn.berlin; Olga Panagiotopoulou, olga.panagiotopoulou@monash.edu

Sci. Adv. **8**, eabm8280 (2022)
DOI: 10.1126/sciadv.abm8280

The PDF file includes:

Figs. S1 to S39
Tables S1 to S9
Legends for data S1 and S2
Legends for movies S1 to S6
References

Other Supplementary Material for this manuscript includes the following:

Data S1 and S2
Movies S1 to S6

1. Supplementary materials

1.1. Expanded Materials and Methods

Figure S1 illustrates a flowchart of the different steps and sensitivity analyses performed in this study. Succinctly, these steps include: (1) the selection of specimens of various periods, lineages, and body sizes; (2) the 3D reconstruction of each component (bones, cartilage, soft tissue pad) for each specimen; (3) the virtual reconstruction of various pedal postures for each specimen; (4) a sensitivity analysis on the size of the mesh; and (5) Finite Element Analysis (FEA) along with sensitivity analyses on tissue material properties and boundary conditions.

Institutional Abbreviations – CM[NH]: Carnegie Museum of Natural History (Pittsburgh, U.S.A.); MB.R., see MfN; MdCL: Musée des Confluences (Lyon, France); MfN, Museum für Naturkunde – Leibniz−Institut für Evolutions− und Biodiversitätsforschung an der Humboldt−Universität zu Berlin (collection numbers: MB.R.) (Berlin, Germany); POL: Collection of the centre de conservation et d'étude (Lons-le-Saunier, Jura, France); QM: Queensland Museum (Brisbane, Australia); YPM: Yale Peabody Museum (New Haven, Connecticut, U.S.A.).

1.1.1. Specimens

Sauropodomorph specimens – This research focuses on five nearly complete sauropodomorph pedes within a broad phylogenetic and spatiotemporal range (Figs. S1–2), including: (1) one non-sauropod (out-group) exemplar with the mirrored left pes of the Late Triassic (~227–208.5Ma) French *Plateosaurus engelhardti* (POL 70, formerly *Dimodosaurus polignensis*; Fig. S2a) (*81*) ; (2) the right pes of the early Late Jurassic (~162.6Ma) Australian gravisaurid *Rhoetosaurus brownei* (QM F1659; Fig. S2b) (*17, 82*); (3) the right pes of the Late Jurassic (~157.3–152.1Ma) North American macronarian *Camarasaurus* sp. (mounted cast at MdCL; we kept this specimen as an unspecified species due to the uncertainty of its affinity, possibly based on *C. grandis* [YPM 1901]; Fig. S2c) (*83*); (4) the right pes of the Late Jurassic (~157.3–152.1Ma) North American diplodocoid *Diplodocus carnegii* (CM[NH] 94, mounted cast at MfN; Fig. S2d) (*1*); and (5) the right pes of the Late Jurassic (~157.3–145Ma) East African macronarian *Giraffatitan brancai* (mounted pes at MfN, likely based on MB.R.5023; Fig. S2e) (*84, 85*). With the exception of *Omeisaurus tianfuensis* (*86*) and *Shunosaurus lii* (*87*), that could not be used in this research due

to access constraints, Early–Middle Jurassic sauropods lack well-preserved pedal materials, which might have otherwise provided more details on this temporal range. Additionally, due to the incompleteness of some specimens, any missing bones were virtually reconstructed based on close relative taxa.

Provisional validation specimen – To validate the analyses, a 3D model of the right pes of an extant elephant was virtually reconstructed in Autodesk Maya 2017 using referenced image planes of anatomical representations of African elephant pedes (*22, 34, 88-90*) (Fig. S2f). This approach was chosen due to lack of CT scan data of an elephant pes.

1.1.2. Model construction

3D Modelling – Three-dimensional (3D) digital surface models of each specimen were generated using photogrammetry (*73, 74*) (Figs. S1–2). Photographs were taken with a Canon EOS 550D (18 MP, f/5.6, 28mm) and an iPhone6 camera (8 MP, f/2.2, 29mm) to produce a spherical coverage of each specimen and individual bones. Approximately 150 photographs per specimen were taken to cover the entire external surface of each element. In Agisoft Photoscan Professional (v1.0.3; www.agisoft.com) the photographs were aligned, generated into dense clouds, triangulated into a surface mesh, given a textured map and outputted to Wavefront Object file format (*.obj). The 3D models were then imported into Autodesk Maya 2017 (www.autodesk.com) where the full set of pedal posture morphotypes was reconstructed.

Pseudo-cartilage thickness – An arbitrary 20 mm space between all pedal bones was applied from an experimental standpoint (*29*). Each cartilaginous joint was modelled in Autodesk Maya 2017 as individual cartilaginous capsules between two adjacent bones, representing the synovial joint of vertebrates (*91*). For a detailed explanation of the choice for this cartilaginous thickness refer to (*29*) (and its associated supplemental materials).

Published data on sauropod cartilage thickness are currently lacking in the literature. Nonetheless, a previous study evaluated the proportion of cartilage thickness in the long limbs of several extant phylogenetic bracketing taxa (*92*), ranging between a maximum of 10.8% of the bone length (in *Alligator mississippiensis*) and 1.8% (in *Coturnix japonicus*). Applying these proportional ranges to the pedal phalanges of our specimens yields mean hypothetical cartilage thicknesses between 3.1–18.4 mm for *P. engelhardti*; 5.7–34.4 mm for *R. brownei*; 3.4–20.3 mm

for *D. carnegii*; 4.8–29 mm for *C.* sp.; 4.4–26.2 mm for *G. brancai* (Tables S1–S5). Given the range of values between specimens, we limited 20 mm as the value of maximum cartilage thickness for ease of comparisons and replications. This value is further supported by the sensitivity analysis conducted by (*29*) who demonstrated unclear differences in ROM values ($p>0.001$; sensu *93*) between large articular thicknesses (i.e., between 20, 30, and 40 mm) in the pes of *R. brownei* (*29: supplemental materials*). Some additional support for a cartilage thickness less than 20 mm is inferred from the preservation of the study specimens. In QM F1659 for instance, the interconnected matrix between the closely articulated phalanges might be somewhat indicative of cartilaginous gaps, irrespective of the degree of taphonomic distortion (*17: p. 371*). Following measurements, these matrix-filled spaces ranged from 5–20 mm. For the elephant model, a maximal cartilage thickness of 1.5 ± 0.5 mm was considered, representing a slightly overestimated value for an adult African elephant as reported in the literature (*94, 95*).

Postural morphotypes – Our main study focuses on the range of mid-digitigrade to subunguligrade continuum; although the two boundaries morphotypes (i.e., plantigrady and unguligrady), were also investigated (see 1.2. Expanded Results for further details).

Pseudo soft tissue pads – Because a soft tissue pad is not preserved in the sauropod body fossil record, we conducted a series of sensitivity analyses on soft tissue pad configuration and material properties based on observations made from the sauropod ichnological record (e.g., *36, 58, 96*) and comparisons with extant animals, such as the feet of elephants (e.g., *23, 26, 97*) and the feet of ostrich (e.g., *98:fig.3, 99:fig.1, 100:fig.3*) (Fig. S3). These included: (1) PAD1, a soft tissue pad positioned caudally below the tarsus and crus complex and extended to the plantar surfaces of the first joints in contact with the substrate (i.e., either metatarsophalangeal or proximal-most interphalangeal joints); (2) PAD2, a soft tissue pad positioned caudally below the tarsus and crus complex and prolonged further underneath the same joints, assumed to act as a secondary digital cushion; (3) PAD3, an incipient soft tissue pad positioned caudally below the tarsus and crus complex, forming a faint slender heel area, and expanded below the plantar surfaces of each digit in contact with the substrate, similar to the phalangeal pad structures seated underneath the pedal phalanges of large ground birds (e.g., casuariiforms) (*101*). PAD3 was principally investigated in

P. engelhardti to reconstruct an incipient soft tissue pad that would have been closer to the sauropodomorph condition (Figs. S3g–l; S34–S35).

Further details on the soft tissue pad material properties are provided in the section "*Materials properties*". Please refer to (*29: supplemental materials*) regarding the method used for the virtual reconstruction of the hypothetical pad in Autodesk Maya 2017.

1.1.3. Finite Element Analysis (FEA)

Model discretization – The surface file of each component (i.e., bone, cartilage, soft tissue pad) was imported into 3-Matic 11.0 software (Materialize Inc., Leuven, Belgium) for the generation of volumetric mesh files. We performed a sensitivity analysis to select the most suitable mesh properties for our models. Based on this analysis, we used continuum linear tetrahedral elements of type C3D4 with a nominal size of 0.5 mm for the bones and cartilaginous capsules and 1 mm for the soft tissue pad (Fig. S4 and Table S6). Each component was then imported into Abaqus/CAE 6.13-6.23 FEA software, preserving the coordinate systems of each segment as defined for each taxon and each postural morphotype. Choices for the mesh properties are detailed below.

Previous FE studies have found that the size of the mesh represents a critical issue in FEA (*102, 103*). Hence, a sensitivity analysis was conducted to assess the effect of distinct mesh size and number of elements on our models using duplicates of the third metatarsal of *Giraffatitan brancai* (for simplicity, computational time and power available) (Table S6). The results show that all mesh models record marginally similar contour plots, demonstrating that mesh density, and thus node arrangements, do not substantially affect the general von Mises stress distribution in our study. However, FEMs with larger nominal size (≥ 1.0 mm) and lower number of elements (< 5300 elements) seem to underestimate strain and displacement as smaller stress areas were consistently recorded compared to finer mesh models (Fig. S4). Comparatively, the use of FEMs with mesh of very fine nominal size (< 0.25 mm) increased exponentially the number of elements (ranging from ~20,000 up to ~500,000 elements for a nominal size of 0.5 mm and 0.1 mm, respectively) (Table S6). Consequently, FEA on these very fines meshes could not be run in ABAQUS due to insufficient memory available. Ultimately, we selected the higher-density mesh that our computational power could handle for each component (i.e., a mesh with a nominal size of 0.5 mm

for the bones and cartilaginous capsules and of 1 mm for the soft tissue pad due to its comparatively larger volume size).

Materials properties – Due to the lack of data on tissue material properties of anatomical constituents in fossil specimens, linear elasticity, homogeneity and isotropy were assumed for each model. To validate the tested material properties values, we surveyed 130^+ FEA papers performed on extant and extinct taxa to document the material properties (i.e., Young's modulus and Poisson ratio) assigned to a variety of bones and soft tissues in the literature (Data S1).

(1) *Bones*: The vast majority of FE analyses in palaeontology suggests that bones of many dinosaurs (and other archosaurs) appear analogous to the Haversian bones of fast-growing bovine mammals (*75-77*). In reality, however, the review of the literature denotes the wide range of material properties values attributed to bones in distinct fossil taxa (Data S1). Without further histological insights about the sauropod pedal elements, we assigned a proxy E value of 10,000 MPa and a v value of 0.3 to each bone model, representative of the most commonly used E value for bones in the literature.

(2) *Pseudo-cartilages*: We deem that these tissues would have presented similar universal cartilaginous properties than most vertebrates (i.e., predominantly sharing elastic functions (*78, 79*)). However, our literature review reveals that Young's modulus values of cartilage vary between studies by a factor of 10 (e.g., 10 MPa (*104*); 1 MPa (*105*); 100 MPa (*106*)) (Data S1). Such a variation in reported cartilage properties is likely contingent on multiple factors, including ontogeny, species, size, or a specific region of a joint, among other (*94, 95*). Consequently, a sensitivity analysis was performed to assess how variations in E values of the cartilaginous capsules affect stresses distribution. Three values of Young's modulus were assigned to our pseudo-cartilages, including: 100 MPa (C1), 10 MPa (C2), and 1 MPa (C3) (Figs. S13–S18). In all cases, a constant Poisson's ratio of 0.4 was assigned, representative of the most commonly E values for cartilages among living taxa (Data S1).

(3) *Pseudo soft tissue pads*: Among living terrestrial tetrapods, only a few organisms seem to possess padding tissues within the palmar/plantar regions of their autopodia that may resemble the structure investigated here. Previous studies interpreted these structures to exhibit nonlinear, viscoelastic behaviours (*47, 80, 107*), somewhat similar to cartilage, and consisting of fibrous

connective tissues (*34, 48, 108*) (Data S1). Owing perhaps on the intra- and inter-variability of these tissues through ontogeny and taxa (*34, 80*), detailed material properties of such padding tissues reveal equivocal Young's modulus values in the literature (*109, 110*) (Data S1). To avert making unfounded assumptions, a sensitivity analysis was performed to assess how variations in in the soft tissue pad elastic moduli affect stresses distributions. Four values of Young's modulus, ranging from highly viscoelastic to more cartilaginous materials, were assigned to our pseudo soft tissue pad, including: 0.1 MPa (F1), 1 MPa (F2), 10 MPa (F3), and 100 MPa (F4) (Figs. S19–S24). In all cases, a constant Poisson's ratio of 0.49 was used, representing the only value attributed to fatty tissues among living taxa in the literature (Data S1).

Boundary conditions – We replicated the weight-bearing phase of locomotion by assuming that the sauropod pes was in complete contact with the substrate. To simulate weight-bearing in the Cartesian system, the dorsal surface of the pes was loaded vertically in the inverse direction of the *z*-axis (i.e., towards the substrate), and its plantar surface, assumed in interaction with the ground, was constrained (with the exception of digit V, which did not interact with the substrate in most postures). Nonetheless, a series of sensitivity analyses was conducted to test the effects of constraint and loading conditions on each model.

(1) *Constraints*: Each model was constrained from rigid body motion to allow movement in the vector direction only. Six boundary conditions (BC1 to BC6) were investigated (Fig. S25):

- BC1: nodes constrained in translation and rotation on a small portion of the surface at the tip of the distalmost phalanges;
- BC2: nodes constrained in translation and rotation on the plantar surfaces of each distal phalanx only (i.e., usually the unguals);
- BC3: nodes constrained in translation and rotation on the plantar surfaces of the interphalangeal joints;
- BC4: nodes constrained in translation and rotation on the plantar surfaces of the mid-portion of the pes only;
- BC5: nodes constrained in translation and rotation on the central portion of the plantar surfaces of all bony elements in assumed contact with the ground;
- BC6: nodes constrained in translation and rotation on the complete plantar surfaces of all bony elements in assumed contact with the ground.

In our FEMs that included a hypothetical soft tissue pad, four distinct constraints conditions (BC1' to BC4') were tested (Fig. S26):

- BC1': nodes constrained in translation and rotation only on the central portion of the plantar surface of the virtual pad in assumed contact with the ground;
- BC2': nodes constrained in translation and rotation only on the full plantar surface of the virtual pad in assumed contact with the ground;
- BC3': nodes constrained in translation and rotation on the central portion of the plantar surfaces of the virtual pad and all bones in assumed contact with the substrate;
- BC4': nodes constrained in translation and rotation on the full plantar surfaces of the virtual pad and of all bones in assumed contact with the substrate.

Similar sensitivity analyses were performed on the FEMs of the simulated elephant pes (with and without soft tissue pad), yet the boundary conditions slightly differed due to the elephant distinct pedal morphology (see Fig. S27).

(2) *Loading*: Forces were applied to surface nodes at the proximal surfaces of each metatarsal and the proximal surface of the soft tissue pad when included (Fig. S28). An initial vertical force of 10,000 N (L1) was used as a proxy between our FEMs to permit direct comparison between each sauropodomorph taxon and with our simulated elephant pes. Force estimates originate from force estimations in living elephants, corresponding to an animal with a body mass of 3,000–4,000 kg (*23*) (Table S7), which was therefore used in this discourse for our simulated elephant pes. In FEMs that included a hypothetical pad, a sensitivity analysis was performed to assess how the applied forces affect stress distributions. Two sets of applied forces were investigated, including:

- L1: an applied force on the proximal surfaces of the metatarsals and the soft tissue pad (Fig. S28);
- L1B: an applied force on the proximal surfaces of the metatarsals alone (Fig. S28).

Ultimately, more physiologically realistic loads were tested for each fossil taxon (L2) using the body mass estimation for each of our specimen. In palaeontology, the '*body mass scaling method*' is generally used to estimate the body mass of a fossil (*41*). This method is based on the empirical scaling relationship of stylopodial circumferences (i.e., humerus and femur) derived from extant tetrapods. As described previously (*41*): Appendix S1, masses in kilograms are estimated using

femoral and humeral circumferences (FC and HC, respectively), which are expressed within the following equations:

$$mass = \frac{10^a}{1000} \quad (1)$$

Where for bipedal taxa:

$$a_{bipedal} = 2.749 \times \log_{10}(FC \times 2^{0.5}) - 1.104 \quad (2)$$

and for quadrupedal taxa:

$$a_{quadrupedal} = 2.749 \times \log_{10}(FC + HC) - 1.104 \quad (3)$$

In this study, the body masses of *P. engelhardti*, *D. carnegii, C.* sp., and *G. brancai* were estimated following the equations above (Table S7). Conversely, the estimation of *R. brownei* body mass was hampered because this specimen only preserves a partial femur. To base our inferences on empirical data, a regression plot was generated using stylopodial circumferences of all known sauropods (*41*) (Fig. S5). We estimated that the humerus circumference of *R. brownei* fitted in a range of 450–600 cm, using its known femur circumference (i.e., 696 mm), (Fig. S5 and Table S7). The body mass of *R. brownei* was therefore projected to be within a range of 20,000–28,000 kg (mean of 24,000 kg) (Tables S7). Finally, all loading forces were calculated by multiplying the estimated body mass of each specimen (*m*, in *kg*) with the gravitational acceleration (i.e., $g = 9.834\ m/s^2$) to obtain a force measurement in Newtons (*N*). The resulting force was divided by four under the assumption that loads were equally distributed between the four autopodia during the support phase (i.e., $W = mg$; with $F = \frac{1}{4}\ W$) (Table S7). However, this load regime may be an underestimation in some of our specimens examined considering that the hindlimbs have been proposed to bear most of the body weight during locomotion in more derived forms (*5, 111*).

1.2. Expanded Results

Brief overview – This section provides an extended summary of the results for each sensitivity analysis. It should be noted that most of the sensitivity analyses were undertaken on all sauropodomorph specimens. However, some features (e.g., pad outlines, constraints) were only investigated in one sauropod taxon (herein *R. brownei*), our non-sauropod (out-group) exemplar

(herein *Plateosaurus engelhardti*) and the simulated elephant pes for simplicity. Figures including skeletal FEMs without soft tissue pad are presented using a von Mises stresses scale of 0–25 MPa; while figures including FEMs with hypothetical soft tissue pad are presented using a von Mises stress scale of 0–1 MPa. Moreover, most sensitivity analyses were performed using a proxy load L1 of 10,000 N to allow strict comparison between specimens. Hence, we refer the reader to Figures S29–S33 for a comparison with more physiologically realistic loading condition (L2).

1.2.1. Postural morphotypes: Skeletal without soft tissue pad

When subjected to a proxy load of 10,000 N, each postural morphotype of each sauropodomorph skeletal FEM showed a marginally similar spatial patterning in von Mises stresses, with maximum stresses >> 100 MPa (Figs. S6–S10). In all cases, the plantigrade, mid-digitigrade, digitigrade, and subunguligrade morphotypes showed the highest concentrations of von Mises stresses in the lateral digits, particularly within the shaft of metatarsals II–IV in *P. engelhardti* and in the shaft of metatarsals III–IV in the other specimens. Contrastingly, the unguligrade morphotypes recorded the highest concentrations of von Mises stresses within the distalmost phalanges in all taxa, particularly in digits II–IV. Under similar conditions, the skeletal FEM of the simulated elephant pes without soft tissue pad showed an increase in von Mises stresses in the central and medial digits II–III, with the highest stresses in the shafts of the metatarsals and the phalanges (Fig. S11).

1.2.2. Postural morphotypes: Skeletal with soft tissue pad

We conducted sensitivity analysis to test the different skeletal postural morphotypes with a soft tissue pad for each sauropodomorph taxon (i.e., from mid-digitigrady to unguligrady; *Note*: by definition the plantigrade morphotype does not include a developed soft tissue pad but this posture was nonetheless studied) (Figs. S6–S10). Irrespective of the postures, we showed that the presence of a hypothetical soft tissue pad substantially reduces von Mises stresses for each taxon compared to loading the skeletal postures alone, with maximum von Mises stresses < 100 MPa. All sauropodomorph FEMs including a hypothetical pad showed the highest concentrations of von Mises stresses in the lateral digits, particularly within the shaft of metatarsals II–IV in *P. engelhardti* and in the shaft of metatarsals III–IV in the other specimens. Similar outcomes were observed in the simulated elephant pes with a pad (Fig. S11). In the latter, von Mises stresses were substantially reduced compared to loading the skeletal FEM only (with maximum von Mises

stresses < 100 MPa), and the general spatial patterning of von Mises stresses was preserved in the central and medial digits II–III and intensified in the shafts of the metatarsals and the phalanges.

Due to the reduction of bone stresses in all of our morphotypes that include a soft tissue pad, we could not nominate a definite skeletal posture for our specimens. However, two of our morphotypes (i.e., plantigrady and unguligrady) can be rejected based on the following rationales. Firstly, in full plantigrady, the metatarsals bended severely in the dorsoplantar direction to the point that their proximal condyles overlapped with the plantar surface of the soft tissue pad (an outcome particularly apparent under physiologically realistic loading conditions; see Figures S29–S33). Moreover, the plantigrade morphotype resulted in increased von Mises stresses in the soft tissue pad in all specimens compared to the other morphotypes, an outcome likely to result from its reduced dorsoplantar thickness. Therefore, this configuration of the pes would have most likely resulted in mechanical failures in the bones and the soft tissue pad (e.g., stress fractures). Thus, a plantigrade posture would not have been vital for the animal's life. Secondly, the inherent anatomy and configuration of the unguligrade morphotype is problematic (as previously advocated (*29*)). In unguligrady, only the distal portion of the ungual of each digit firmly contacts the substrate, resulting in higher von Mises stresses in the distalmost phalanges compared to the other morphotypes. These results suggest that the large mediolaterally compressed unguals, combined with their bevelled and concave articular facets, are not anatomically adapted for loading the tips of the unguals alone. Additionally, among modern unguligrade tetrapods, the distalmost phalanges include hoofed and keratinous-covered material that engage the substrate via a flat distal surface (e.g., Equidae, among other unguligrade artiodactyls; (*112, 113*)). Lastly, the unguligrade morphotype implies that all metapodia and phalanges are impeded in the soft tissue pad, thus precluding any large motion of the unguals. This arrangement contradicts with the ichnological record according to which clear digit impressions have been recorded in pedal tracks from Middle Jurassic to Upper Cretaceous strata (*36, 96*). Thus, we conclude that the plantigrade and unguligrade configurations in our specimens are unlikely given their anatomy. These results corroborate and supplement a previous study (*29*).

1.2.3. Soft tissue pad outlines

The sensitivity analyses of varying the outlines of the soft tissue pad (PAD1 and PAD2) were investigated in *R. brownei*. The results revealed that, regardless of the outline, the presence of a

soft tissue pad substantially reduces von Mises stresses in all postural morphotypes compared to loading the skeletal postures alone, with maximum von Mises stresses < 100 MPa. Both pad outlines showed similar spatial patterning of von Mises stresses but differed in stress magnitude (Fig. S12). Indeed, our analyses revealed that PAD2 (i.e., a pad extending from the tarsus-crus complex and prolonged further underneath the most distal joints) increased bone stresses compared to PAD1 (i.e., a pad extending from the tarsus-crus complex to the plantar surfaces of the first joints in contact with the substrate). Therefore, we chose to apply PAD1 to all other specimens for ease of comparison and replication.

The sensitivity analysis of varying the soft tissue pad configurations to a more sauropodomorph-like condition (PAD3) was investigated in *P. engelhardti* and our sauropod case exemplar *R. brownei*. Irrespective of the pedal posture, the presence of an incipient soft tissue pad resulted in a reduction of bone stresses compared to loading the skeletal postures alone, with maximum von Mises stresses < 60 MPa in *P. engelhardti* and < 200 MPa in *R. brownei*. Both PAD1 and PAD3 outlines showed similar spatial patterning of von Mises stresses but differed in stress magnitudes (Figs. S34–35). Indeed, PAD3 recorded slightly higher concentrations of von Mises stresses than PAD1 in *P. engelhardti* and *R. brownei* (Fig. S39). This result was expected considering the larger volume of PAD1 and its interdigital connection, which would have allowed a greater surface area to absorb mechanical shocks. This outcome is consistent with our initial hypothesis that early diverging non-sauropod sauropodomorphs, such as *P. engelhardti*, would have likely presented an incipient soft tissue pad that was likely similar in general material properties to but distinct in configuration from that seen in sauropods. Our results show that the presence of phalangeal pads could have effectively reduced bone stresses in *P. engelhardti*.

Whilst PAD3 reduced bone stresses in *R. brownei* compared to loading the skeletal pes alone, the maximum von Mises stress values recorded in the bones of *R. brownei* with the presence of PAD3 (i.e., ~200 MPa) were within the limits to what cortical bones have been proposed to withstand before fractures (i.e., ~150–200 MPa) (51, 52). This state suggests that the presence of PAD3 would have been inadequate to reduce effectively bone stresses in *R. brownei*, which would have most likely resulted in mechanical failures (e.g., stress fractures). As a result, we consider this hypothetical pad configuration unlikely and rule out the presence of an incipient, sauropodomorph-like, pad for *R. brownei*, and by extension to other larger more derived sauropods. Nevertheless,

the similar mechanical properties recorded for PAD3 could support the idea that a more substantial pad (PAD1) might have originated from the gradual adaptation of a plesiomorphic sauropodomorph-like condition (PAD3).

1.2.4. Cartilage properties

The sensitivity analysis of varying Young's modulus (*E*) of the cartilaginous capsules was investigated for each sauropodomorph taxon and the simulated elephant pes (Figs. S13–S17). Results showed that von Mises stress patterns were insensitive to variations in *E* values of the cartilaginous capsules. All FEMs reported the highest concentrations of von Mises stresses in the lateral digits, particularly within the shaft of metatarsals II–IV in *P. engelhardti* and in the shaft of metatarsals III–IV in the other specimens. For all FEMs, the analyses showed that cartilaginous capsules with a lower *E* value (10 MPa [C2] and 1 MPa [C3]) recorded a marginally higher concentration of von Mises stresses compared to higher *E* value (100 MPa [C1]). Similar outcomes were observed in the simulated elephant pes (Fig. S18). Therefore, we chose to apply an *E* value of 100 MPa (C1) to the cartilaginous capsule of all FEMs for ease of comparison and replication.

1.2.5. Soft tissue pad properties

We conducted sensitivity analyses to test the effect of varying the soft tissue pad *E* values on bone stresses (Figs. S19–S23). We found that soft tissue pad properties did not impact bone stresses distributions, but affected stress magnitudes. All FEMs reported the highest concentrations of von Mises stresses in the central and lateral digits, particularly within the shaft of metatarsals II–IV in *P. engelhardti* and in the shaft of metatarsals III–IV in the other specimens. In all cases, soft tissue pads with lower *E* values (0.1 MPa [F1] and 1 MPa [F2]) showed marginally higher concentration of von Mises stresses than higher *E* values (10 MPa [F3] and 100 MPa [F4]), with the condition F4 (E = 100 MPa) recording the lowest concentration of von Mises stresses. Similar outcomes were observed in the simulated elephant pes (Fig. S24). Therefore, we chose to assign an *E* value of 100 MPa (F4) to the soft tissue pad of all FEMs for ease of comparison and replication.

1.2.6. Constraints: Skeletal without soft tissue pad

We tested the effect of constraints on bone stresses in *R. brownei* and the simulated elephant pes (Fig. S25). We found that constraint location had an effect on bone stress magnitudes but not on stress distribution. All FEMs showed the highest concentrations of von Mises stresses in the lateral

digits, particularly within the shaft of metatarsals III–IV. Strikingly, constraints BCs 1–3 (i.e., constraining nodes in the plantar surfaces of the most distal elements of the pes) recorded the highest concentration of von Mises stresses than constraints BCs 4–6 (i.e., constraining nodes in the plantar surfaces of all the elements in contact with the ground). Similar outcomes were observed in the simulated elephant pes where constraining the plantar surfaces of all the elements in contact with the ground decreased von Mises bone stresses (Fig. S27). We applied the more cost-effective condition BC5 for all other taxa in the main text for ease of comparison and replication as strain results between BC5 and BC6 were similar, yet BC6 increased substantially computational time in our FEA.

1.2.7. Constraints: Skeletal with soft tissue pad

We tested the boundary conditions on skeletal morphotypes with a soft tissue pad in *R. brownei* and the simulated elephant pes (Fig. S26). Irrespective of the type of constraints, the results showed a reduction in bone stresses for each postural morphotype compared to loading the skeletal postures alone, with maximum von Mises stresses < 100 MPa. Constraints BCs' 1–2 (i.e., constraining the nodes on the plantar surfaces of the soft tissue pad alone) recorded marginally reduced stress magnitudes compared to constraints BCs' 3–4 (i.e., constraining the nodes on the plantar surfaces of the digits and the soft tissue pad). Similar outcomes were observed in the simulated elephant pes (Fig. S27). Therefore, we chose to assign the more realistic condition BC4' to all other specimens by constraining the plantar surfaces of all the components in complete contact with the substrate (i.e., pad + bones and cartilages) for ease of comparison and replication.

1.2.8. Applied forces on soft tissue pad

We tested the loading conditions on the skeletal postural morphotype with a soft tissue pad in *R. brownei* and the simulated elephant pes (Fig. S28). Irrespective of the loading conditions, the results showed a reduction in bone stresses for all postural morphotypes with a soft tissue pad compared to loading the skeletal postures alone, with maximum von Mises stresses < 100 MPa. Loading condition L1 (i.e., an applied force on the proximal surfaces of the metatarsals and the soft tissue pad) recorded lower concentrations in von Mises stresses compared to L1B (i.e., an applied force on the proximal surfaces of the metatarsals). Moreover, the application of L1B bended the metatarsals in the dorsoplantar direction. Similar outcomes were observed in the

simulated elephant pes. Therefore, we chose to apply the loading conditions L1 and L2 to all FEMS for ease of comparison and replication.

1.2.9. Applied forces: proxy vs. realistic – Skeletal without soft tissue pad

The results showed that the applications of more physiologically realistic loading forces on our models without a pad retained the same stress distribution but differed in stress magnitude compared to our proxy load (Figs. S29–S33).

1.2.10. Applied forces: proxy vs. realistic – Skeletal with soft tissue pad

As above, the results showed that the applications of more physiologically realistic loading forces on our models with a pad retained the same stress distribution but differed in stress magnitude compared to our proxy load (Figs. S29–S33). These results are expected because each model has been subjected to strictly similar conditions. Hence, variations of the loading forces should not change the stress distribution and only affect stress magnitude.

1.2.11. Additional notes: Trend towards a reduction and loss of the number of autopodial phalanges in sauropod evolutionary history

The uniform pattern of stress recorded between FEMs may correlate with the conspicuous trend towards a reduction and loss of the number of autopodial phalanges in sauropod evolutionary history (*18, 30, 40*). Given that a decrease in von Mises stresses is recorded on the distal phalanges when a pad is included, it is proposed that the shock absorbing aptitude of a soft tissue pad would have impeded the weight-bearing purposes of the distalmost phalanges. This mechanical state could explain the general reduction in the size of the phalanges (*18*), making them susceptible to be lost throughout the course of sauropod evolution.

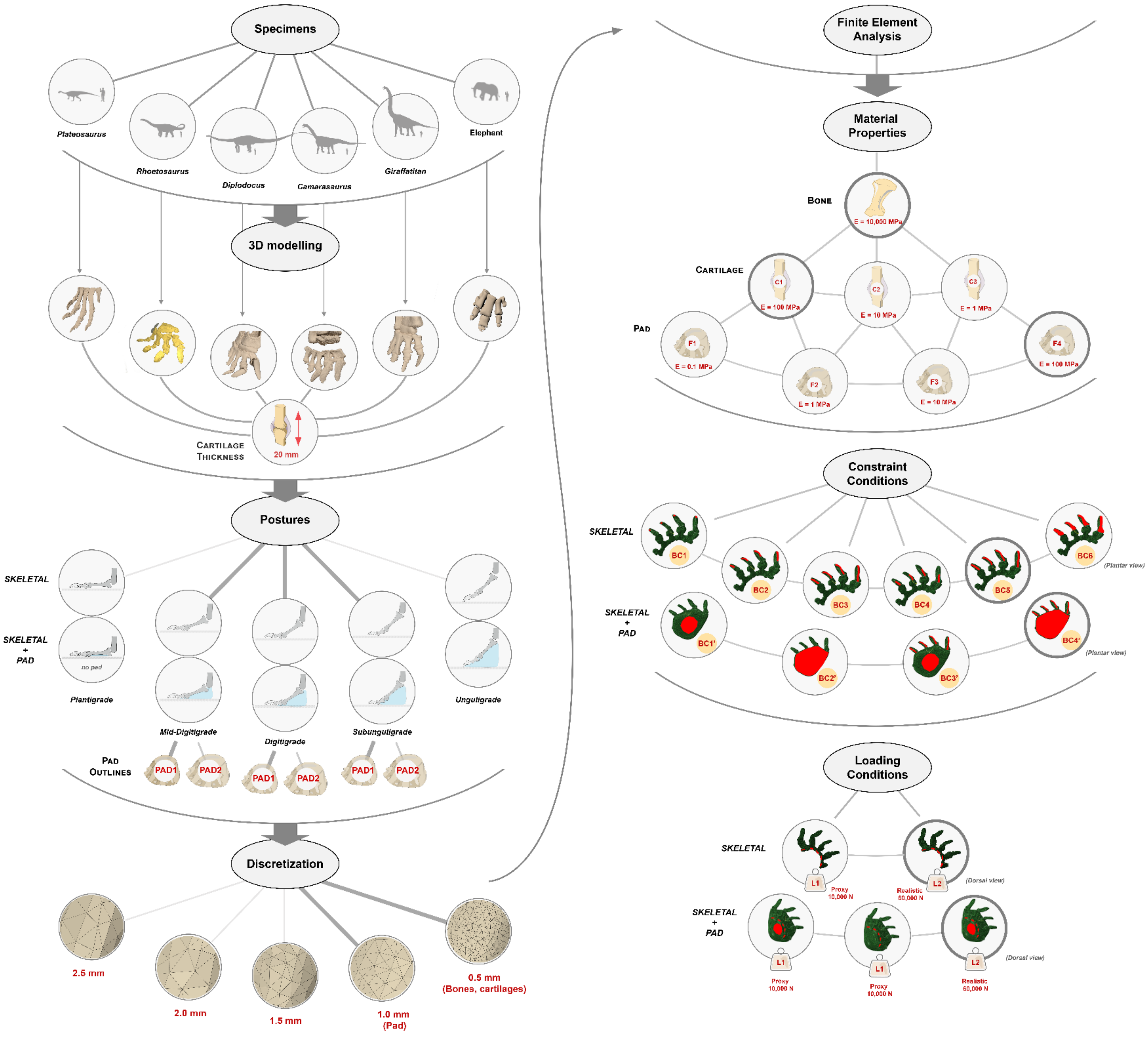

Fig. S1. Flowchart illustrating all analytical steps and sensitivity analyses.

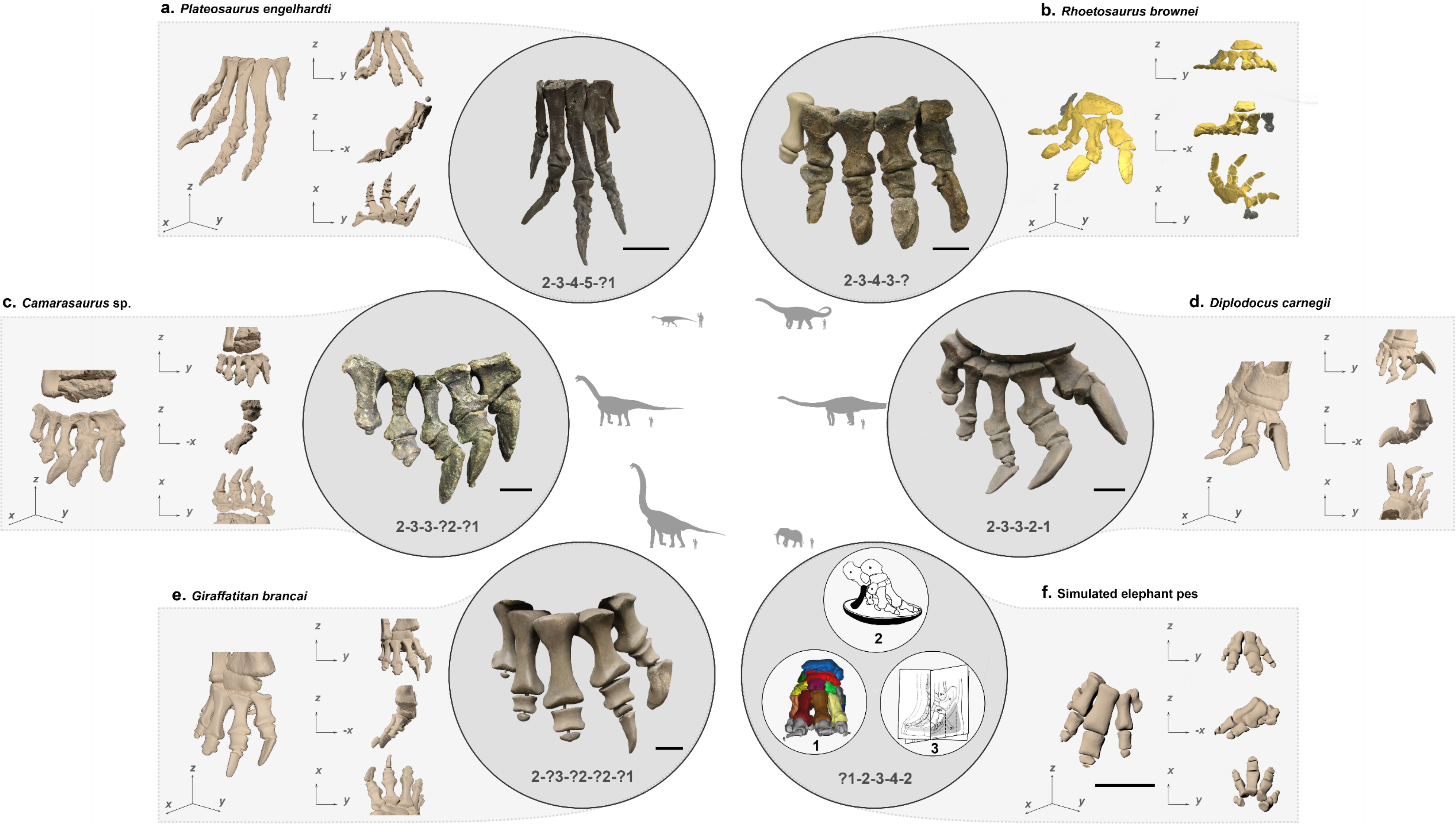

Fig. S2. Details of the five sauropodomorph pedes specimens and simulated elephant pes used in this study. (**a-f**) Original specimens illustrated in each circle and their respective 3D generated models illustrated aside, including each 3D model in craniomedial view (left side); dorsal view (bottom right side); medial view (central right side); and cranial view (top right side). The phalangeal formulae are reported for each taxon, where the number corresponds to the count of phalanges from digits I to V (thus opposite to the models here). "?" indicates uncertain count of phalanges due to incompleteness of the fossil record. (**a**) Complete left pes of *P. engelhardti* (POL 70, formerly *D. polignensis*) (*81*); (**b**) right pes of *R. brownei* (QM F1659) (*17*, *29*, *82*); (**c**) right pes of *C.* sp. (mounted cast at MdCL, likely ?YPM 1901, but this affinity is uncertain); (**d**) right pes of *D. carnegii* (CM[NH] 94, mounted cast at MfN) (*1*); (**e**) right pes of *G. brancai* (reconstructed mounted pes at MfN, likely based on MB.R.5023) (*85*); (**f**) simulated right pes of an extant elephant, reconstructed based on: 1. the supplemental movie S1 from (*22*) (colored model); 2. the illustration from (*89*); and 3. the illustrations from (*34*).

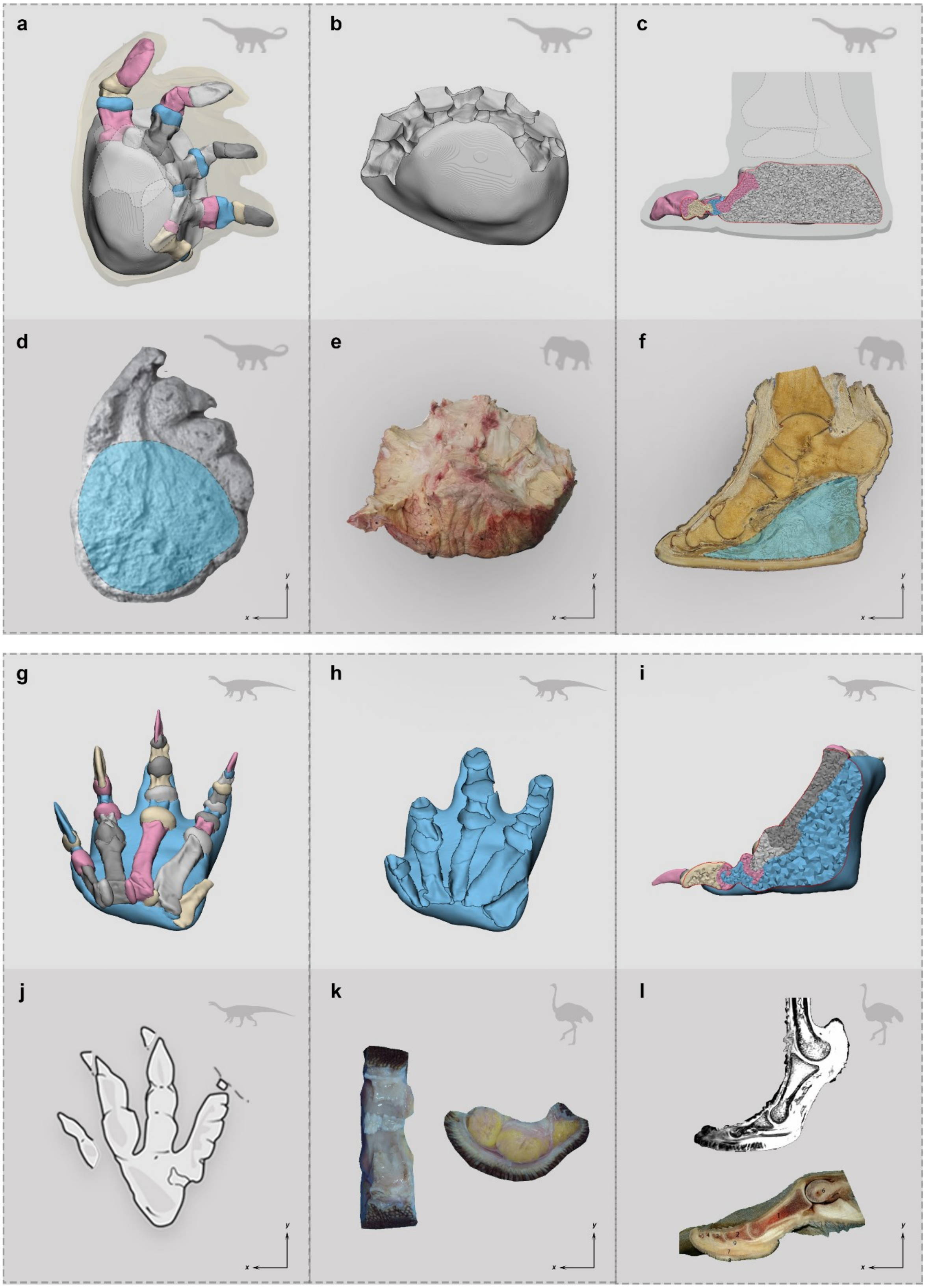

Fig. S3. Virtual soft tissue pads reconstructions. (a-c) Virtual soft tissue pad reconstruction (e.g., pes of *Rhoetosaurus brownei* in the digitigrade morphotype), including: **(a)** position of the virtual soft tissue pad in dorsal view, with pseudo-track outlined; **(b)** internal view of the virtual soft tissue pad in dorsal view; **(c)** cross-section of the virtual soft tissue pad in medial view. **(d-f)** Comparative data used for the reconstruction of the virtual sauropod-like soft tissue pad, including: **(d)** ichnological record, illustrating the natural cast of *Brontopodus* sp. as an exemplar (CU cast 194-2; *96*); **(e-f)** extant animals, here involving the pes of an elephant, with: **(e)** dissected soft tissue pad in caudodistal view (© J. Hutchinson, from *whatsinjohnsfreezer.com*); **(f)** Sagittal plane cross-section of an elephant pes (*97*). **(g-i)** Virtual incipient soft tissue pad reconstruction (e.g., pes of *Plateosaurus engelhardti* in the digitigrade morphotype), including: **(a)** position of the virtual soft tissue pad in dorsal view; **(b)** internal view of the virtual soft tissue pad in dorsal view; **(c)** cross-section of the virtual soft tissue pad in medial view. **(j-l)** Comparative data used for the reconstruction of the virtual incipient soft tissue pad, including: **(d)** ichnological record, illustrating *Evazoum siriguii* (*58*); **(k-l)** extant animals, here involving the pes of an ostrich, with: **(k)** dissected cross section of the soft tissue pad of an ostrich pes in dorsal & proximal views (*100*); **(f)** Sagittal plane CT-scan & cross-section of an ostrich pes (*98*, *99*). Blue outline indicates pad area.

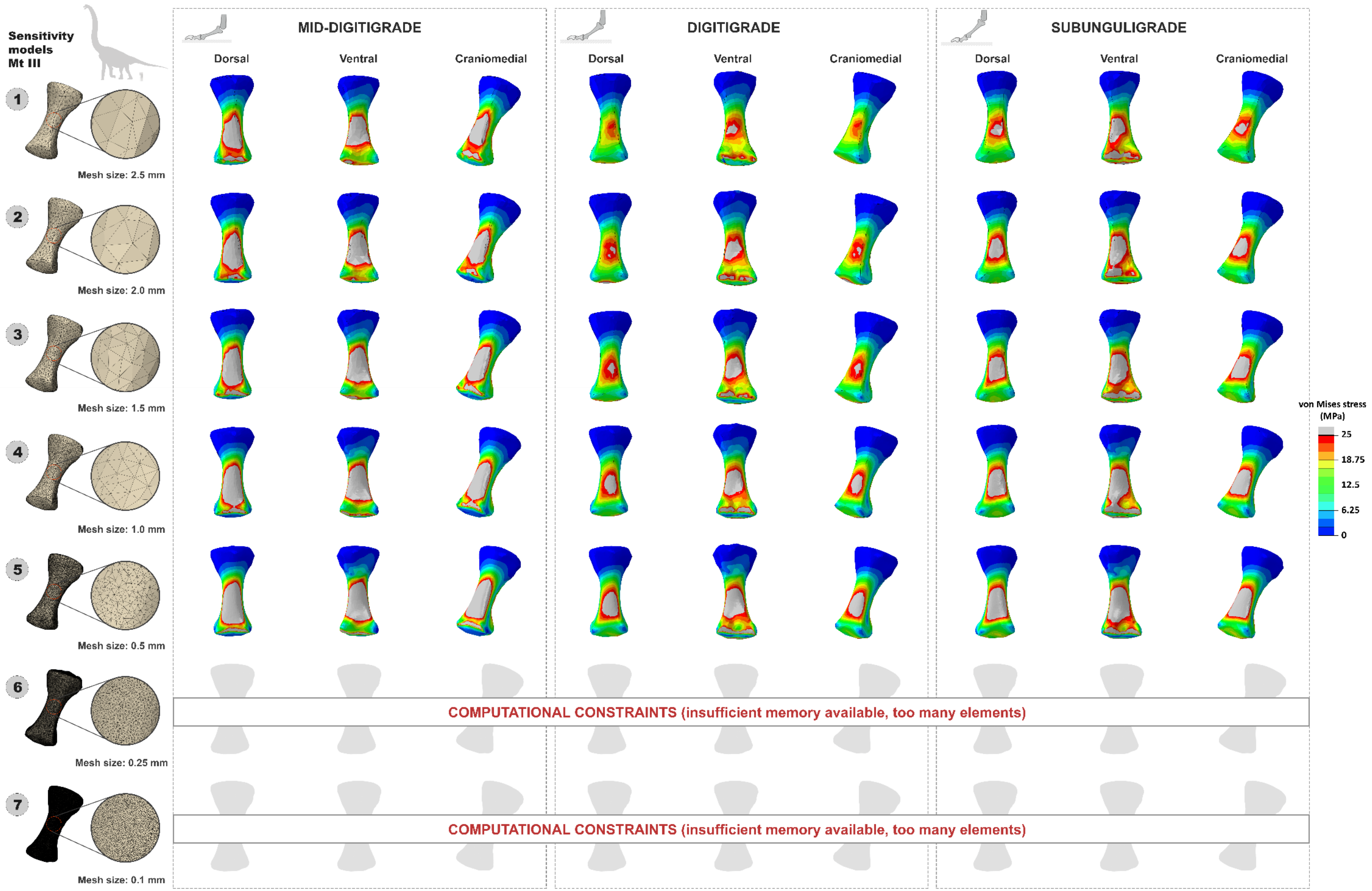

Fig. S4. Von Mises stress (MPa) distribution results for the sensitivity analysis of varying mesh density, using metatarsal III of *Giraffatitan brancai* as an exemplar. Duplicates of seven distinct mesh density ranging from 0.1 to 2.5 mm illustrated in rows from bottom to top. Results for each postural morphotype represented in distinct rectangle, including: mid-digitigrade (left rectangle); digitigrade (middle rectangle); and subunguligrade (right rectangle). For each postural morphotypes and each mesh size duplicate, contour plots of von Mises stresses results illustrated in dorsal (left), ventral (centre), and dorsomedial (right) views, respectively. See labels on the figures for clarity. See Table S6 for details on the respective number of elements.

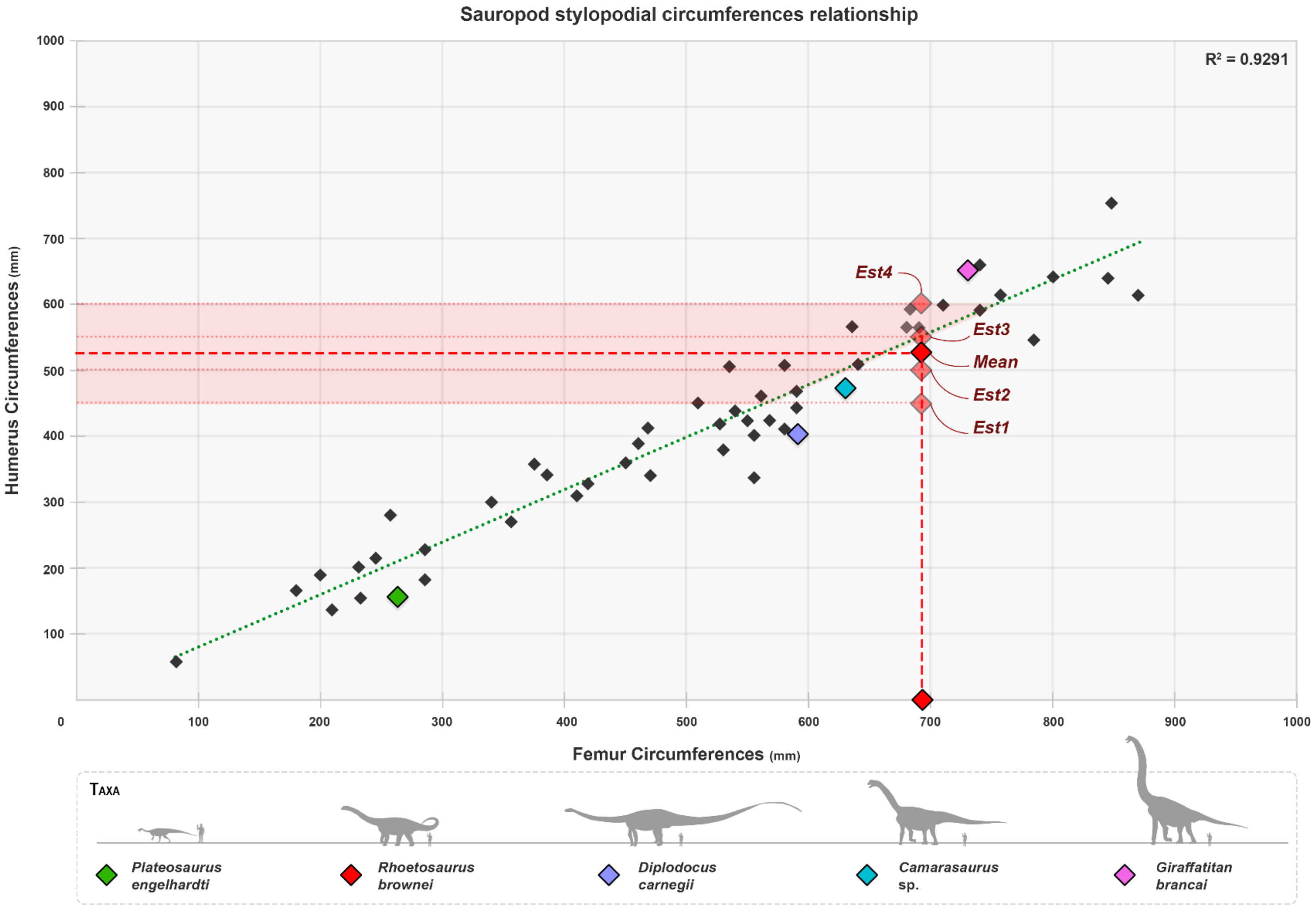

Fig. S5. Sauropod stylopodial circumferences regression. The five sauropodomorphs specimens of interests are illustrated in colours, including: (1) *P. engelhardti* in green; (2) *R. brownei* in red; (3) *D. carnegii* in purple; (4) *C.* sp. in blue; and (5) *G. brancai* in pink. Estimations of *R. brownei* humerus circumference are indicated as the projection of its femur circumference (vertical red dotted line) on the linear regression (green dotted line) where the estimated values of its humerus circumference are indicated in red (horizontal red dotted lines and red background; with values ranging between 450 to 600 mm).

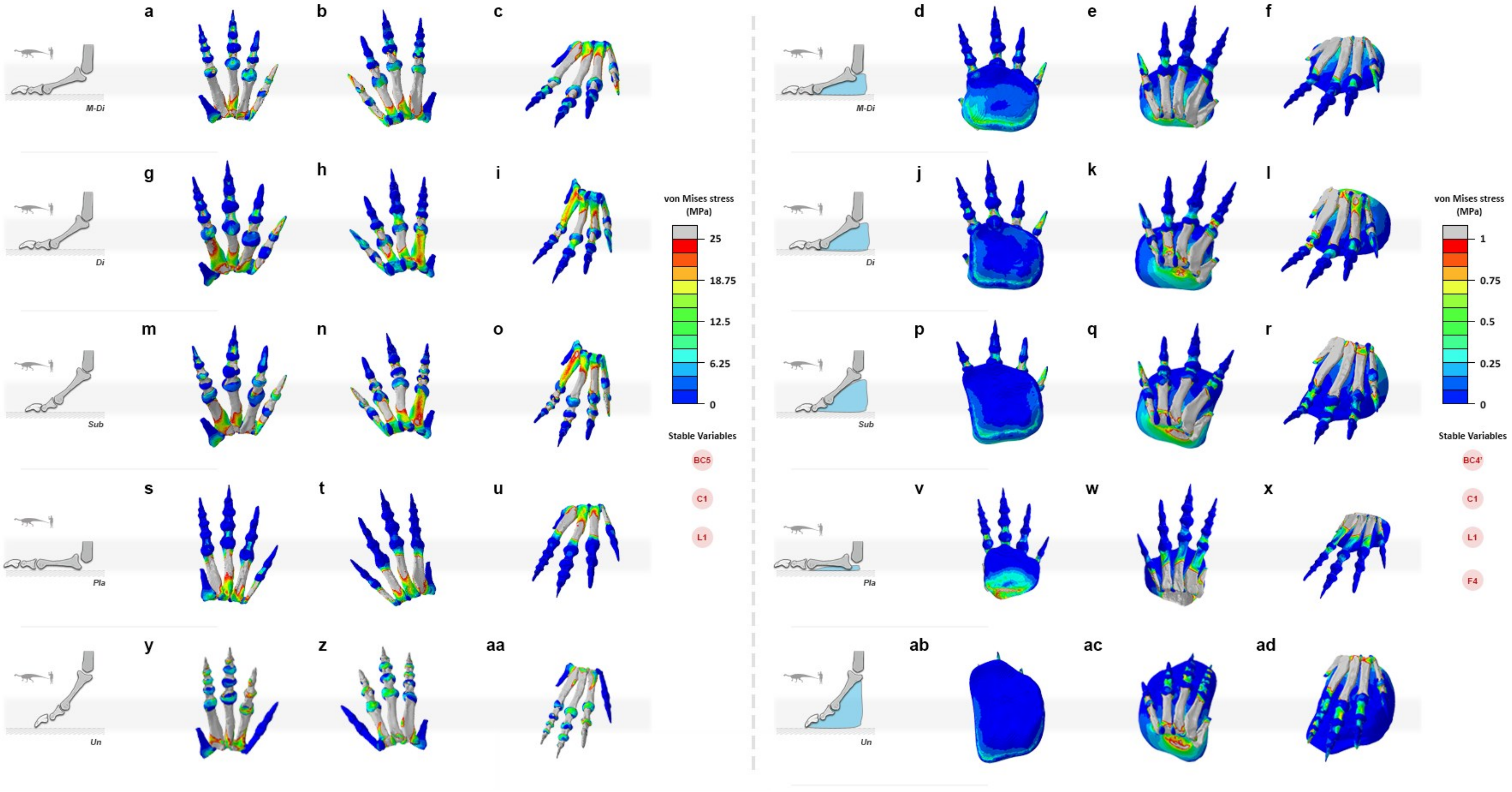

Fig. S6. Von Mises stress (MPa) distribution results for the sensitivity analysis of all postural morphotypes of the skeletal FEMs without (left) and with a hypothetical soft tissue pad (right) for *Plateosaurus engelhardti*. (a-f) Von Mises stress (MPa) distribution results for a mid-digitigrade morphotype, in: (a, d) plantar view; (b, e) dorsal view; and (c, f) craniomedial view. (g-l) Von Mises stress (MPa) distribution results for a digitigrade morphotype, in: (g, j) plantar view; (h, k) dorsal view; and (i, l) craniomedial view. (m-r) Von Mises stress (MPa) distribution results for a subunguligrade morphotype, in: (m, p) plantar view; (n, q) dorsal view; and (o, r) craniomedial view. (s-x) Von Mises stress (MPa) distribution results for a plantigrade morphotype, in: (s, v) plantar view; (t, w) dorsal view; and (u, x) craniomedial view. (y-ad) Von Mises stress (MPa) distribution results for an unguligrade morphotype, in: (y, ab) plantar view; (z, ac) dorsal view; and (aa, ad) craniomedial view. Cold (blue) and warm (red) colours show lower and higher von Mises stresses, respectively. Abbreviations: M-Di, Mid-Digitigrady; Di, Digitigrady; Sub, Subunguligrady; Pla, Plantigrady; Un, Unguligrady; BC5 & BC4', Boundary conditions 5 and 4', respectively; C1, Cartilage condition 1 (*E* value of 100 Mpa); F4, Soft tissue pad condition 4 (*E* value of 100 Mpa); L1, Loading condition 1 (applied force of 10,000 N). Note: distinct scales of von Mises stresses used between left and right. See Figure S29 for a comparison with more physiologically realistic loading condition (L2).

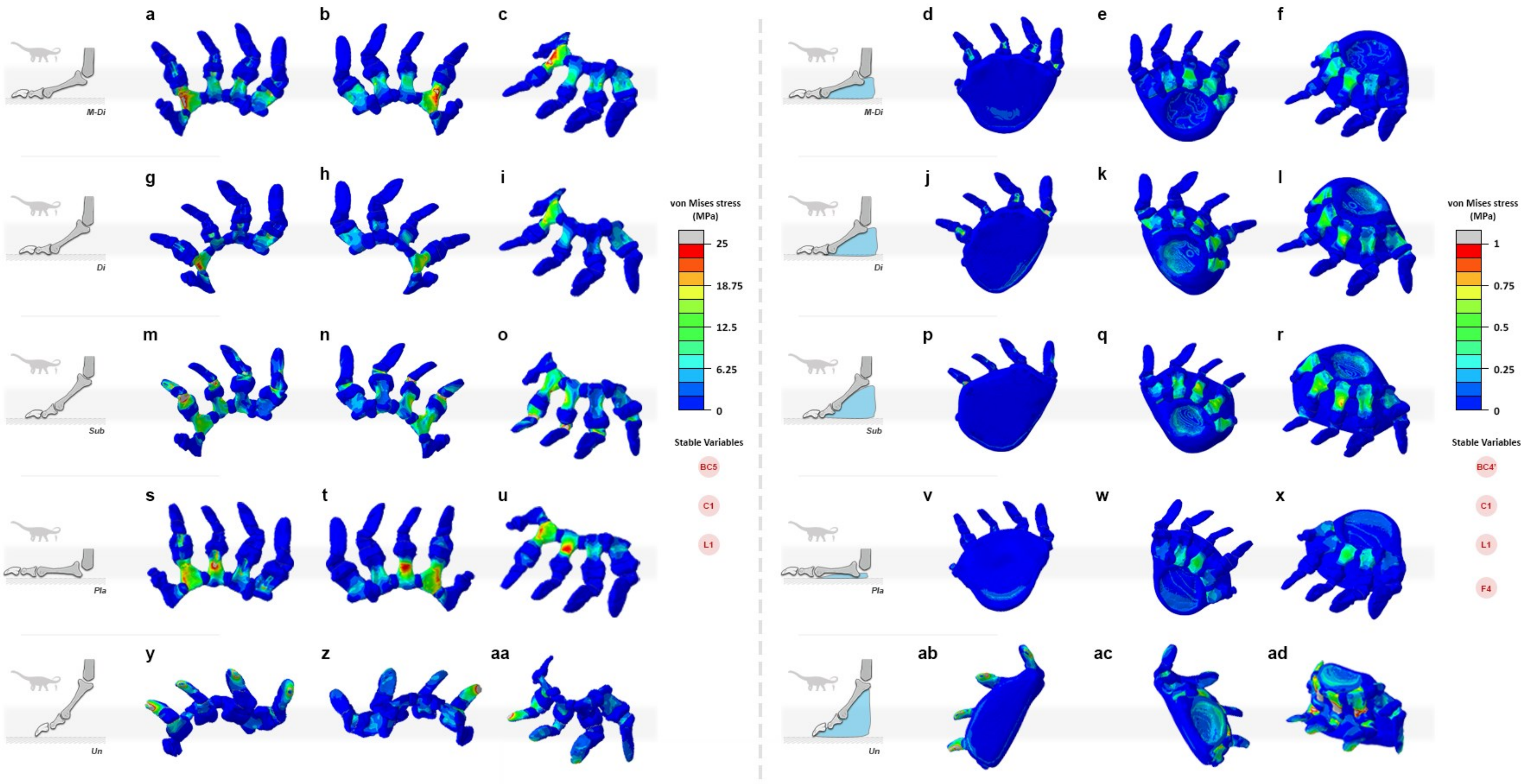

Fig. S7. Von Mises stress (MPa) distribution results for the sensitivity analysis of all postural morphotypes of the skeletal FEMs without (left) and with a hypothetical soft tissue pad (right) for *Rhoetosaurus brownei*. (a-f) Von Mises stress (MPa) distribution results for a mid-digitigrade morphotype, in: (a, d) plantar view; (b, e) dorsal view; and (c, f) craniomedial view. (g-l) Von Mises stress (MPa) distribution results for a digitigrade morphotype, in: (g, j) plantar view; (h, k) dorsal view; and (i, l) craniomedial view. (m-r) Von Mises stress (MPa) distribution results for a subunguligrade morphotype, in: (m, p) plantar view; (n, q) dorsal view; and (o, r) craniomedial view. (s-x) Von Mises stress (MPa) distribution results for a plantigrade morphotype, in: (s, v) plantar view; (t, w) dorsal view; and (u, x) craniomedial view. (y-ad) Von Mises stress (MPa) distribution results for an unguligrade morphotype, in: (y, ab) plantar view; (z, ac) dorsal view; and (aa, ad) craniomedial view. Cold (blue) and warm (red) colours show lower and higher von Mises stresses, respectively. Abbreviations: M-Di, Mid-Digitigrady; Di, Digitigrady; Sub, Subunguligrady; Pla, Plantigrady; Un, Unguligrady; BC5 & BC4', Boundary conditions 5 and 4', respectively; C1, Cartilage condition 1 (*E* value of 100 Mpa); F4, Soft tissue pad condition 4 (*E* value of 100 Mpa); L1, Loading condition 1 (applied force of 10,000 N). Note: distinct scales of von Mises stresses used between left and right. See Figure S30 for a comparison with more physiologically realistic loading condition (L2).

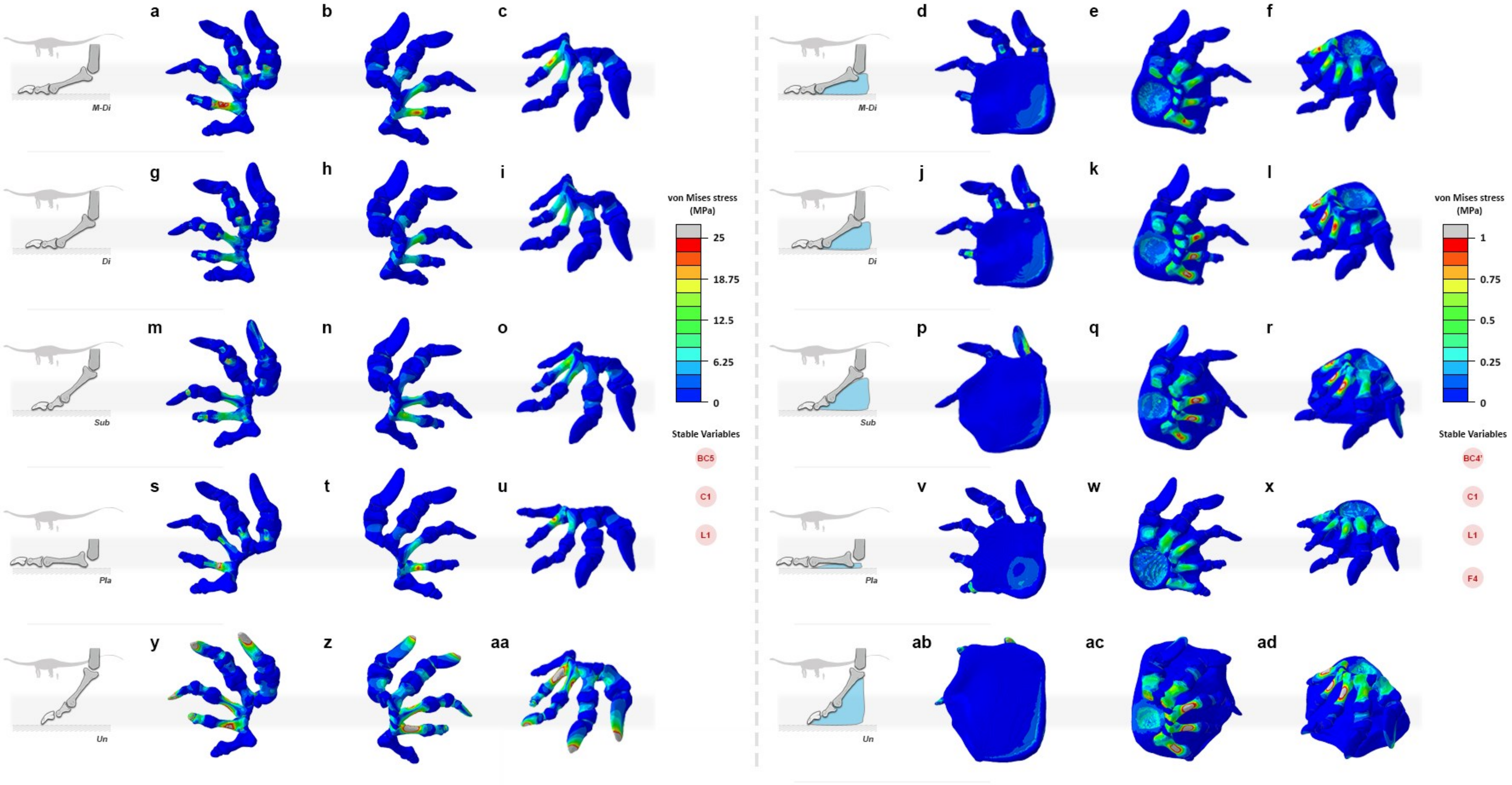

Fig. S8. Von Mises stress (MPa) distribution results for the sensitivity analysis of all postural morphotypes of the skeletal FEMs without (left) and with a hypothetical soft tissue pad (right) for *Diplodocus carnegii*. (a-f) Von Mises stress (MPa) distribution results for a mid-digitigrade morphotype, in: (a, d) plantar view; (b, e) dorsal view; and (c, f) craniomedial view. (g-l) Von Mises stress (MPa) distribution results for a digitigrade morphotype, in: (g, j) plantar view; (h, k) dorsal view; and (i, l) craniomedial view. (m-r) Von Mises stress (MPa) distribution results for a subunguligrade morphotype, in: (m, p) plantar view; (n, q) dorsal view; and (o, r) craniomedial view. (s-x) Von Mises stress (MPa) distribution results for a plantigrade morphotype, in: (s, v) plantar view; (t, w) dorsal view; and (u, x) craniomedial view. (y-ad) Von Mises stress (MPa) distribution results for an unguligrade morphotype, in: (y, ab) plantar view; (z, ac) dorsal view; and (aa, ad) craniomedial view. Cold (blue) and warm (red) colours show lower and higher von Mises stresses, respectively. Abbreviations: M-Di, Mid-Digitigrady; Di, Digitigrady; Sub, Subunguligrady; Pla, Plantigrady; Un, Unguligrady; BC5 & BC4', Boundary conditions 5 and 4', respectively; C1, Cartilage condition 1 (*E* value of 100 Mpa); F4, Soft tissue pad condition 4 (*E* value of 100 Mpa); L1, Loading condition 1 (applied force of 10,000 N). Note: distinct scales of von Mises stresses used between left and right. See Figure S31 for a comparison with more physiologically realistic loading condition (L2).

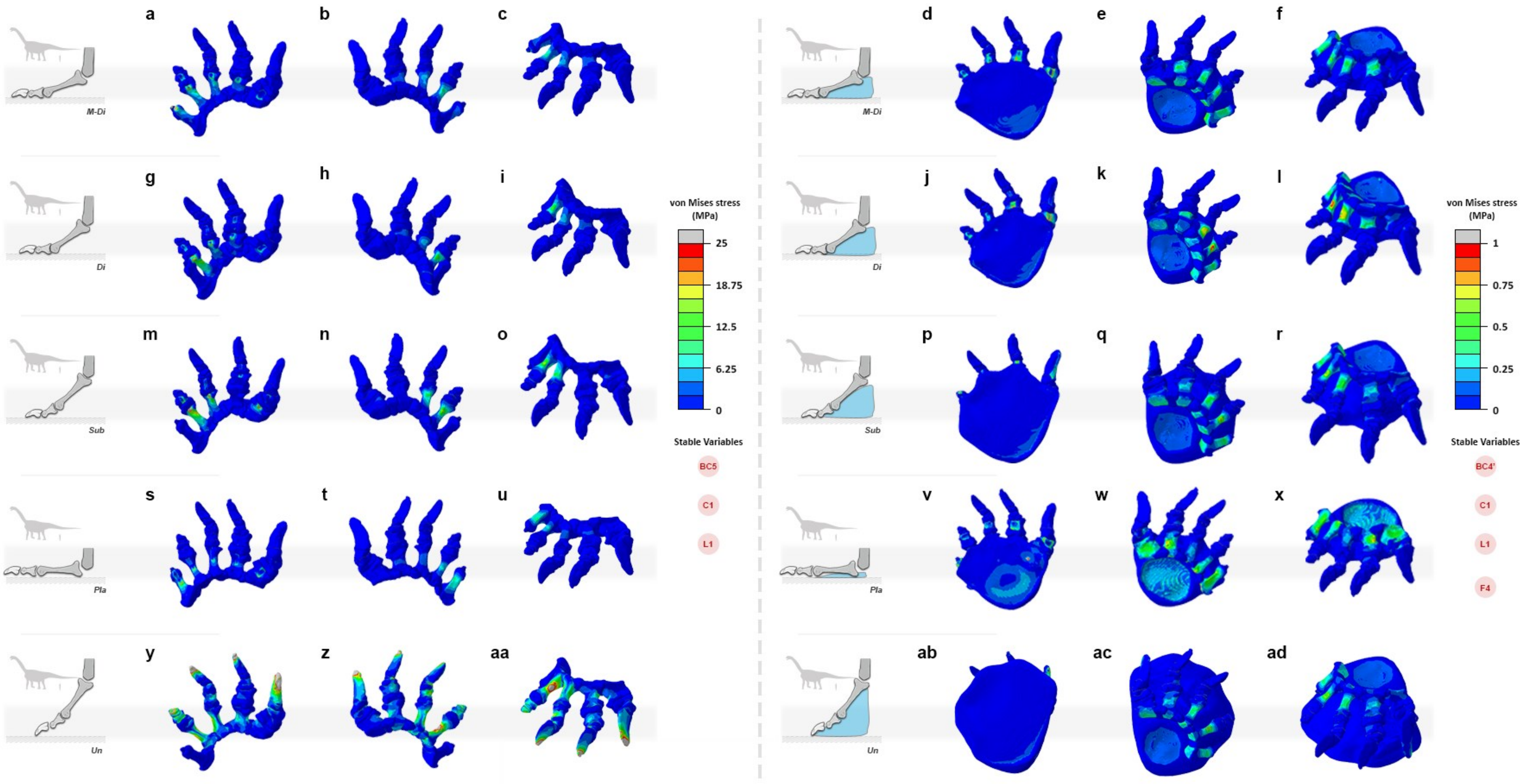

Fig. S9. Von Mises stress (MPa) distribution results for the sensitivity analysis of all postural morphotypes of the skeletal FEMs without (left) and with a hypothetical soft tissue pad (right) for *Camarasaurus* sp. (a-f) Von Mises stress (MPa) distribution results for a mid-digitigrade morphotype, in: (a, d) plantar view; (b, e) dorsal view; and (c, f) craniomedial view. (g-l) Von Mises stress (MPa) distribution results for a digitigrade morphotype, in: (g, j) plantar view; (h, k) dorsal view; and (i, l) craniomedial view. (m-r) Von Mises stress (MPa) distribution results for a subunguligrade morphotype, in: (m, p) plantar view; (n, q) dorsal view; and (o, r) craniomedial view. (s-x) Von Mises stress (MPa) distribution results for a plantigrade morphotype, in: (s, v) plantar view; (t, w) dorsal view; and (u, x) craniomedial view. (y-ad) Von Mises stress (MPa) distribution results for an unguligrade morphotype, in: (y, ab) plantar view; (z, ac) dorsal view; and (aa, ad) craniomedial view. Cold (blue) and warm (red) colours show lower and higher von Mises stresses, respectively. Abbreviations: M-Di, Mid-Digitigrady; Di, Digitigrady; Sub, Subunguligrady; Pla, Plantigrady; Un, Unguligrady; BC5 & BC4', Boundary conditions 5 and 4', respectively; C1, Cartilage condition 1 (*E* value of 100 Mpa); F4, Soft tissue pad condition 4 (*E* value of 100 Mpa); L1, Loading condition 1 (applied force of 10,000 N). Note: distinct scales of von Mises stresses used between left and right. See Figure S32 for a comparison with more physiologically realistic loading condition (L2).

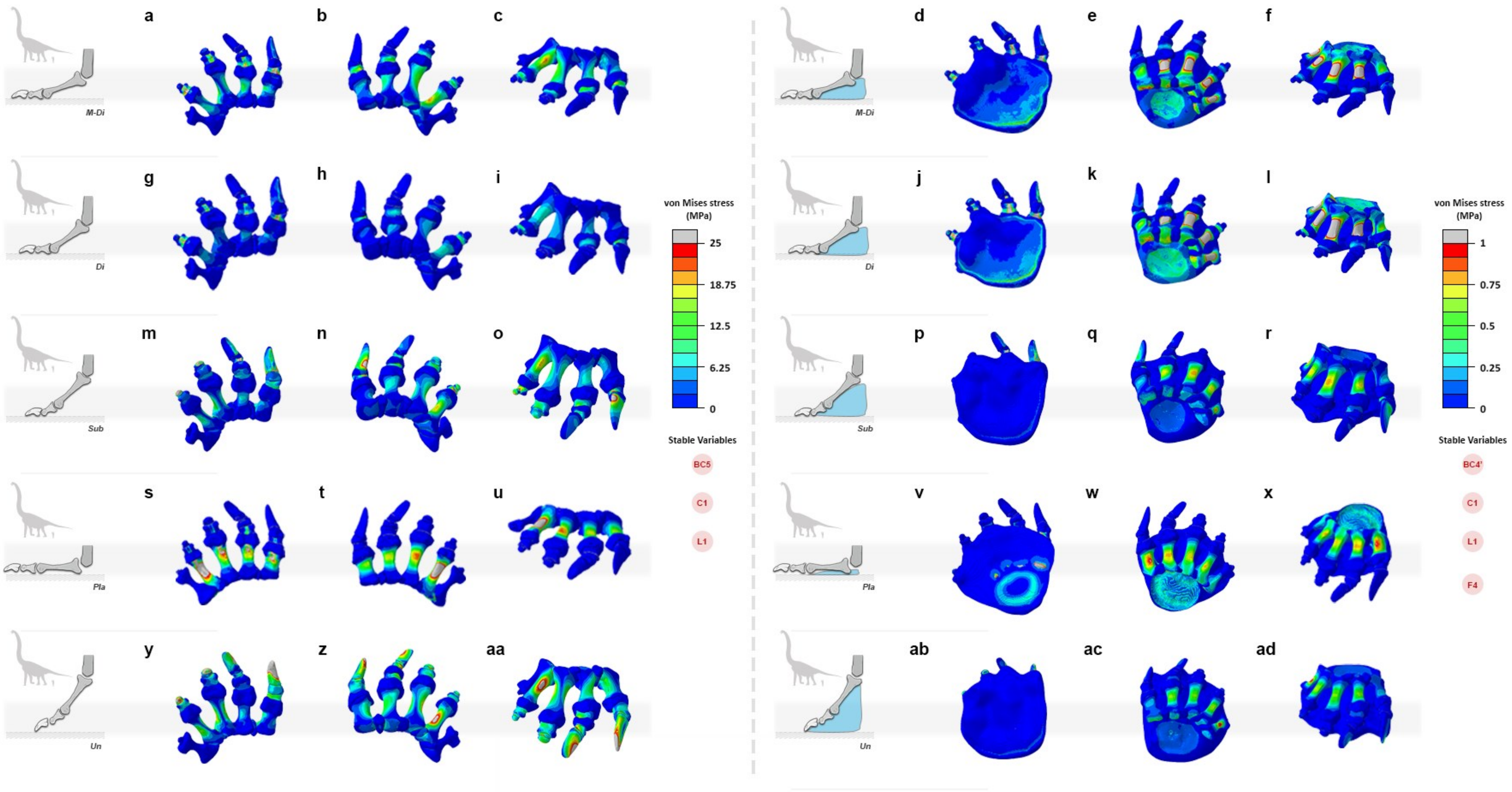

Fig. S10. Von Mises stress (MPa) distribution results for the sensitivity analysis of all postural morphotypes of the skeletal FEMs without (left) and with a hypothetical soft tissue pad (right) for *Giraffatitan brancai*. (a-f) Von Mises stress (MPa) distribution results for a mid-digitigrade morphotype, in: (a, d) plantar view; (b, e) dorsal view; and (c, f) craniomedial view. (g-l) Von Mises stress (MPa) distribution results for a digitigrade morphotype, in: (g, j) plantar view; (h, k) dorsal view; and (i, l) craniomedial view. (m-r) Von Mises stress (MPa) distribution results for a subunguligrade morphotype, in: (m, p) plantar view; (n, q) dorsal view; and (o, r) craniomedial view. (s-x) Von Mises stress (MPa) distribution results for a plantigrade morphotype, in: (s, v) plantar view; (t, w) dorsal view; and (u, x) craniomedial view. (y-ad) Von Mises stress (MPa) distribution results for an unguligrade morphotype, in: (y, ab) plantar view; (z, ac) dorsal view; and (aa, ad) craniomedial view. Cold (blue) and warm (red) colours show lower and higher von Mises stresses, respectively. Abbreviations: M-Di, Mid-Digitigrady; Di, Digitigrady; Sub, Subunguligrady; Pla, Plantigrady; Un, Unguligrady; BC5 & BC4', Boundary conditions 5 and 4', respectively; C1, Cartilage condition 1 (*E* value of 100 Mpa); F4, Soft tissue pad condition 4 (*E* value of 100 Mpa); L1, Loading condition 1 (applied force of 10,000 N). Note: distinct scales of von Mises stresses used between left and right. See Figure S33 for a comparison with more physiologically realistic loading condition (L2).

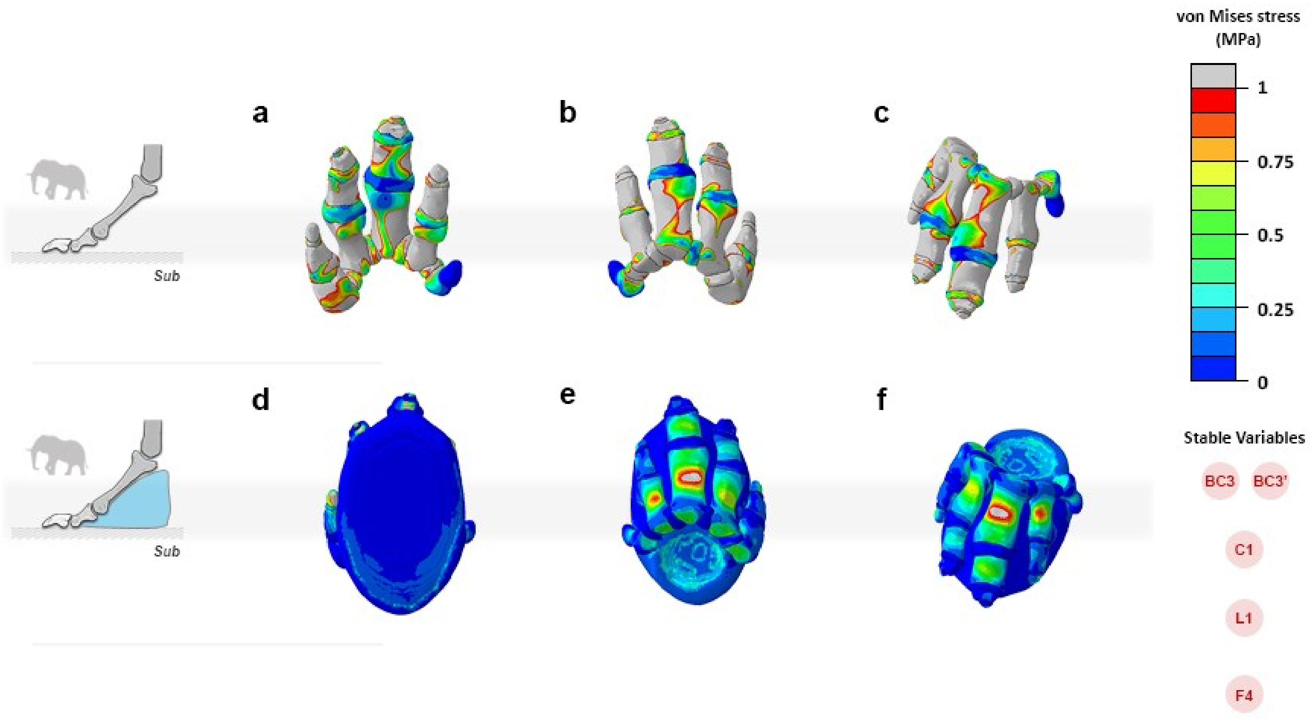

Fig. S11. Von Mises stress (MPa) distribution results for the sensitivity analysis between the skeletal FEM without (top) and with a hypothetical soft tissue pad (bottom) for the simulated elephant pes. (a-c) Von Mises stress (MPa) distribution results for a skeletally subunguligrade morphotype, in: (a) plantar view; (b) dorsal view; and (c) craniomedial view. (d-f) Von Mises stress (MPa) distribution results for a skeletally subunguligrade morphotype with hypothetical pad, in: (d) plantar view; (e) dorsal view; and (f) craniomedial view. Cold (blue) and warm (red) colours show lower and higher von Mises stresses, respectively. Abbreviations: Sub, Subunguligrady; BC3 & BC3', Boundary conditions 3 and 3', respectively; C1, Cartilage condition 1 (*E* value of 100 Mpa); F4, Soft tissue pad condition 4 (*E* value of 100 Mpa); L1, Loading condition 1 (applied force of 10,000 N).

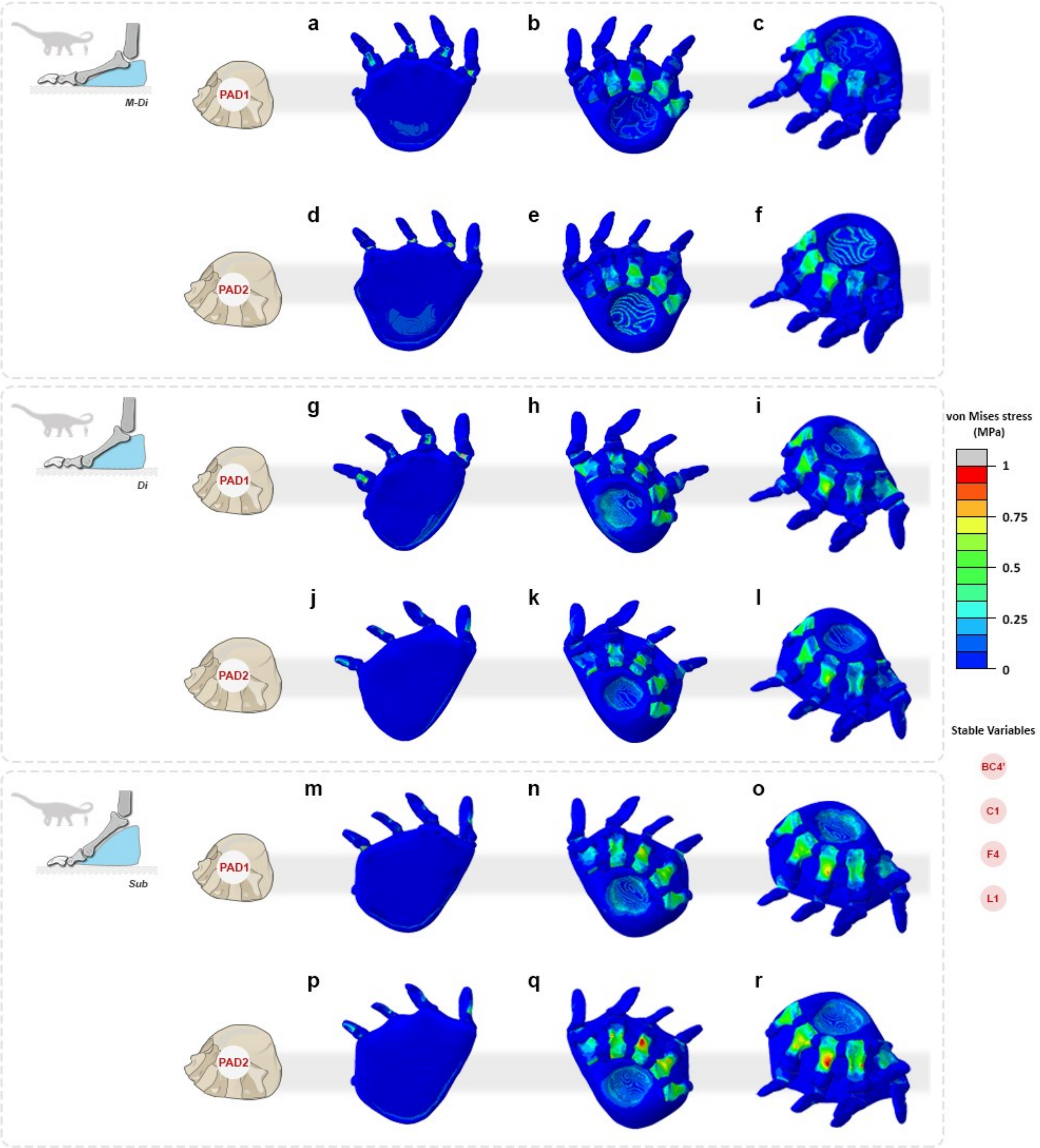

Fig. S12. Von Mises stress (MPa) distribution results for the sensitivity analysis of the soft tissue pad outlines in the mid-digitigrade, digitigrade, and subunguligrade postural morphotypes of *Rhoetosaurus brownei*. (a-f) FEM of a mid-digitigrade morphotype, with: (a-c) Von Mises stress (MPa) distribution results for a pad extended from the tarsus-crus complex to the plantar surfaces of the first joints in contact with the substrate (PAD1), in: (a) plantar view; (b) dorsal view; and (c) craniomedial view; (d-f) Von Mises stress (MPa) distribution results for a pad extended from the tarsus-crus complex and further prolonged underneath the same joints (PAD2), in: (d) plantar view; (e) dorsal view; and (f) craniomedial view. (g-l) FEM of a digitigrade morphotype, with: (g-i) Von Mises stress (MPa) distribution results for PAD1, in: (g) plantar view; (h) dorsal view; and (i) craniomedial view; (j-l) Von Mises stress (MPa) distribution results for PAD2, in: (j) plantar view; (k) dorsal view; and (l) craniomedial view. (m-r) FEM of a subunguligrade morphotype, with: (m-o) Von Mises stress (MPa) distribution results for PAD1, in: (m) plantar view; (n) dorsal view; and (o) craniomedial view; (p-r) Von Mises stress (MPa) distribution results for PAD2, in: (p) plantar view; (q) dorsal view; and (r) craniomedial view. Cold (blue) and warm (red) colours show lower, and higher von Mises stresses, respectively. Abbreviations: M-Di, Mid-Digitigrady; Di, Digitigrady; Sub, Subunguligrady; BC4', Boundary condition 4'; C1, Cartilage condition 1 (*E* value of 100 Mpa); F4, Soft tissue pad condition 4 (*E* value of 100 Mpa); L1, Loading condition 1 (applied force of 10,000 N); PAD1–2, Pad outlines conditions 1–2.

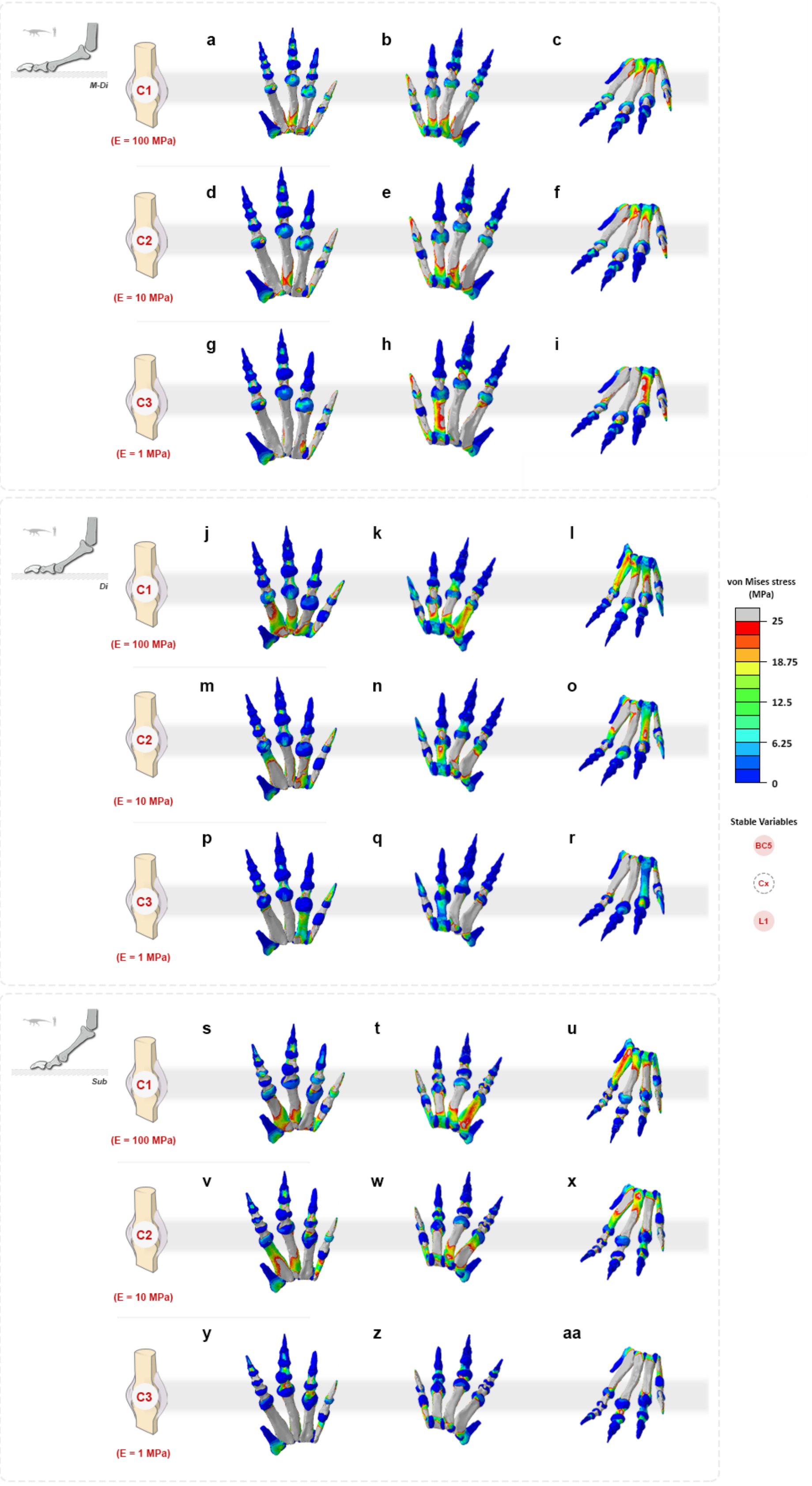

Fig. S13. Von Mises stress (MPa) distribution results for the sensitivity analysis of the cartilage properties (*E*) in *Plateosaurus engelhardti*. (a-i) Von Mises stress (MPa) distribution results for a mid-digitigrade morphotype, including: (a-c) cartilages with *E* = 100 MPa, in: (a) plantar view; (b) dorsal view; and (c) craniomedial view. (d-f) cartilages with *E* = 10 MPa, in: (d) plantar view; (e) dorsal view; and (f) craniomedial view. (g-i) cartilages with *E* = 1 MPa, in: (g) plantar view; (h) dorsal view; and (i) craniomedial view. (j-r) Von Mises stress (MPa) distribution results for a digitigrade morphotype, including: (j-l) cartilages with *E* = 100 MPa, in: (j) plantar view; (k) dorsal view; and (l) craniomedial view. (m-o) cartilages with *E* = 10 MPa, in: (m) plantar view; (n) dorsal view; and (o) craniomedial view. (p-r) cartilages with *E* = 1 MPa, in: (p) plantar view; (q) dorsal view; and (r) craniomedial view. (s-aa) Von Mises stress (MPa) distribution results for a subunguligrade morphotype, including: (s-u) cartilages with *E* = 100 MPa, in: (s) plantar view; (t) dorsal view; and (u) craniomedial view. (v-x) cartilages with *E* = 10 MPa, in: (v) plantar view; (w) dorsal view; and (x) craniomedial view. (y-aa) cartilages with *E* = 1 MPa, in: (y) plantar view; (z) dorsal view; and (aa) craniomedial view. Cold (blue) and warm (red) colours show lower and higher von Mises stresses respectively. Abbreviations: M-Di, Mid-Digitigrady; Di, Digitigrady; Sub, Subunguligrady; BC5, Boundary condition 5; C1–3, Cartilages conditions 1–3 (*E* values of 100 MPa, 10 MPa, and 1 Mpa, respectively); L1, Loading condition 1 (applied force of 10,000 N).

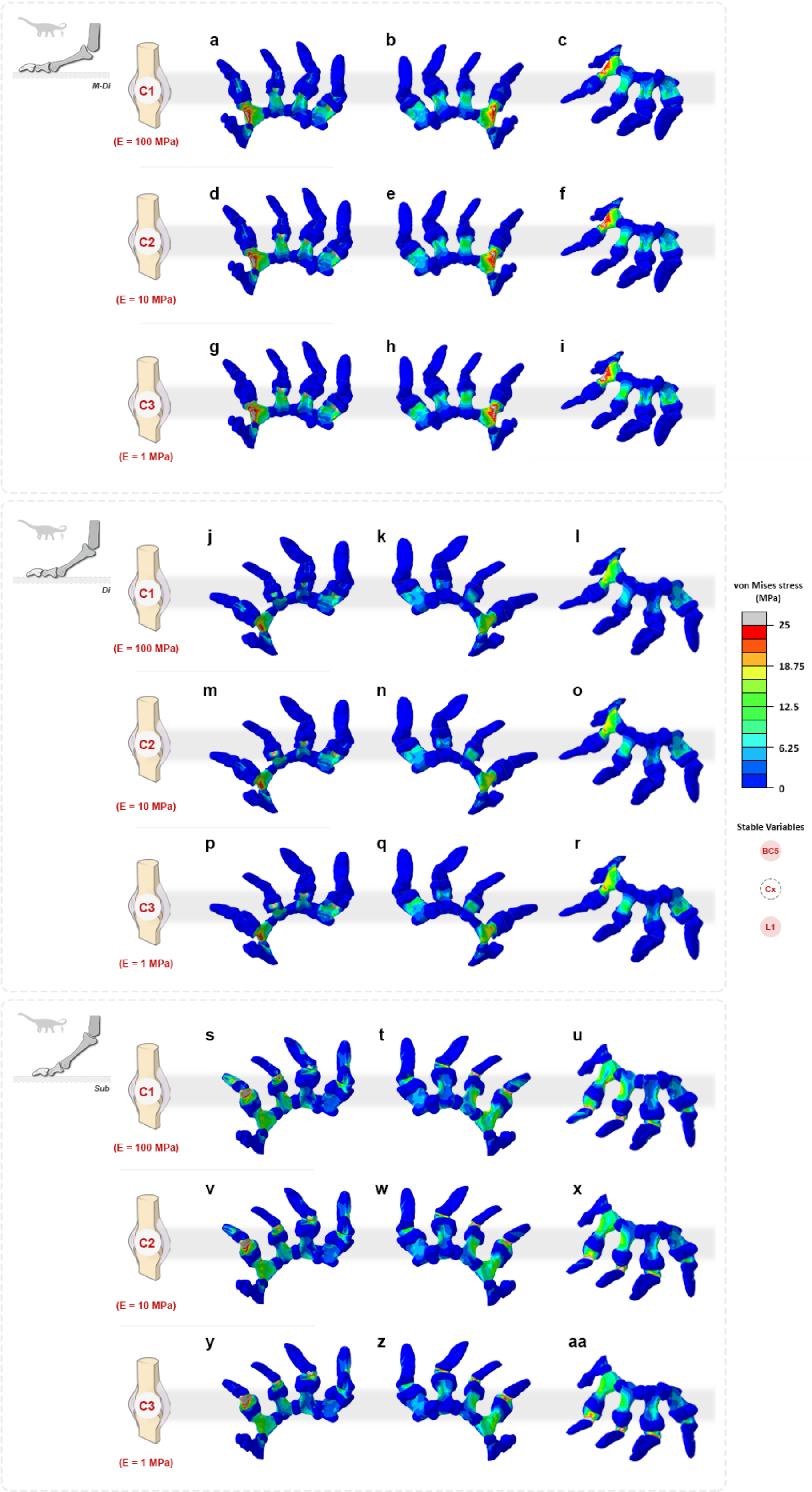

Fig. S14. Von Mises stress (MPa) distribution results for the sensitivity analysis of the cartilage properties (*E*) in *Rhoetosaurus brownei*. (a-i) Von Mises stress (MPa) distribution results for a mid-digitigrade morphotype, including: (a-c) cartilages with $E = 100$ MPa, in: (a) plantar view; (b) dorsal view; and (c) craniomedial view. (d-f) cartilages with $E = 10$ MPa, in: (d) plantar view; (e) dorsal view; and (f) craniomedial view. (g-i) cartilages with $E = 1$ MPa, in: (g) plantar view; (h) dorsal view; and (i) craniomedial view. (j-r) Von Mises stress (MPa) distribution results for a digitigrade morphotype, including: (j-l) cartilages with $E = 100$ MPa, in: (j) plantar view; (k) dorsal view; and (l) craniomedial view. (m-o) cartilages with $E = 10$ MPa, in: (m) plantar view; (n) dorsal view; and (o) craniomedial view. (p-r) cartilages with $E = 1$ MPa, in: (p) plantar view; (q) dorsal view; and (r) craniomedial view. (s-aa) Von Mises stress (MPa) distribution results for a subunguligrade morphotype, including: (s-u) cartilages with $E = 100$ MPa, in: (s) plantar view; (t) dorsal view; and (u) craniomedial view. (v-x) cartilages with $E = 10$ MPa, in: (v) plantar view; (w) dorsal view; and (x) craniomedial view. (y-aa) cartilages with $E = 1$ MPa, in: (y) plantar view; (z) dorsal view; and (aa) craniomedial view. Cold (blue) and warm (red) colours show lower and higher von Mises stresses respectively. Abbreviations: M-Di, Mid-Digitigrady; Di, Digitigrady; Sub, Subunguligrady; BC5, Boundary condition 5; C1–3, Cartilages conditions 1–3 (*E* values of 100 MPa, 10 MPa, and 1 Mpa, respectively); L1, Loading condition 1 (applied force of 10,000 N).

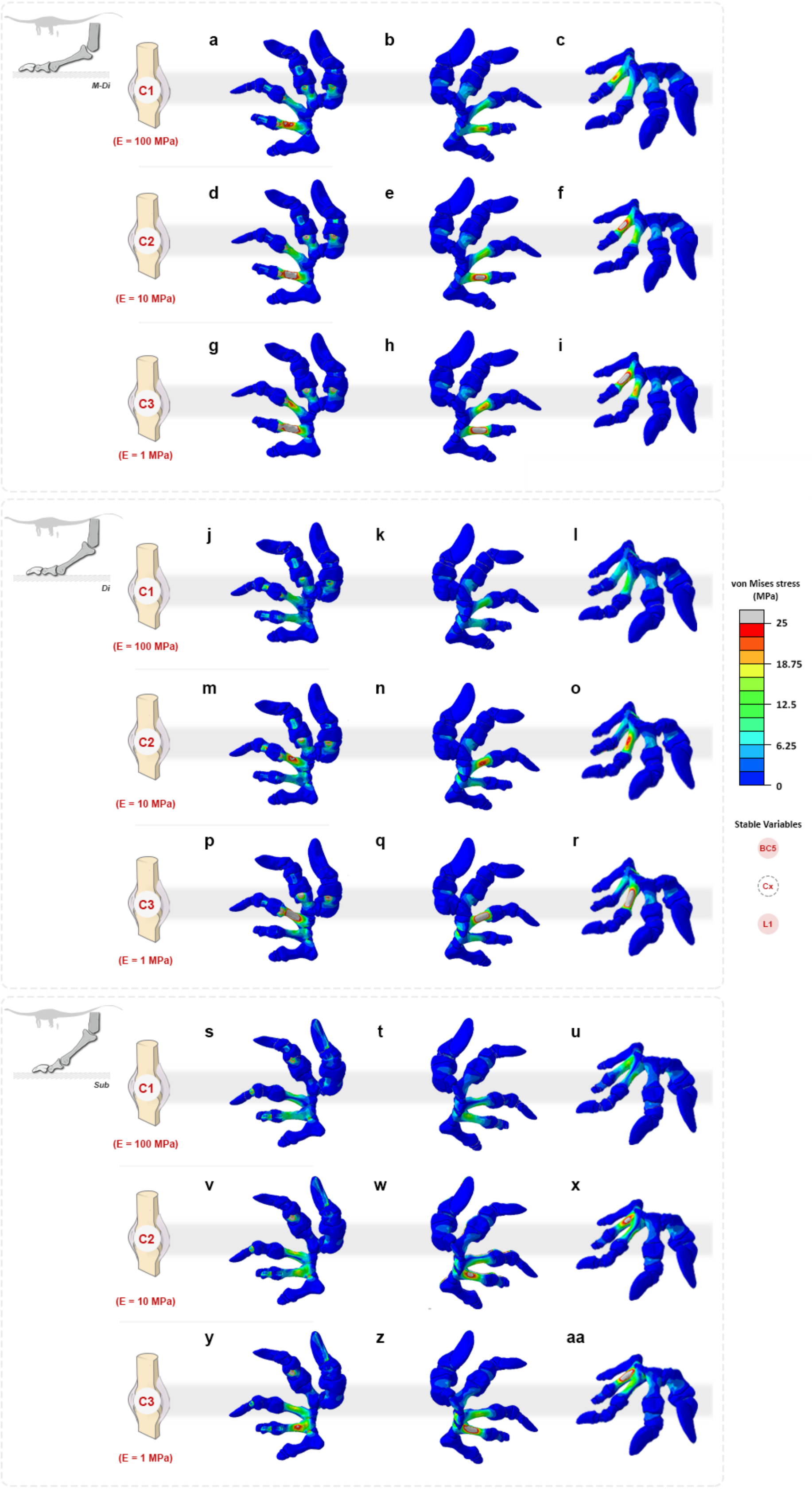

Fig. S15. Von Mises stress (MPa) distribution results for the sensitivity analysis of the cartilage properties (*E*) in *Diplodocus carnegii*. (a-i) Von Mises stress (MPa) distribution results for a mid-digitigrade morphotype, including: (a-c) cartilages with *E* = 100 MPa, in: (a) plantar view; (b) dorsal view; and (c) craniomedial view. (d-f) cartilages with *E* = 10 MPa, in: (d) plantar view; (e) dorsal view; and (f) craniomedial view. (g-i) cartilages with *E* = 1 MPa, in: (g) plantar view; (h) dorsal view; and (i) craniomedial view. (j-r) Von Mises stress (MPa) distribution results for a digitigrade morphotype, including: (j-l) cartilages with *E* = 100 MPa, in: (j) plantar view; (k) dorsal view; and (l) craniomedial view. (m-o) cartilages with *E* = 10 MPa, in: (m) plantar view; (n) dorsal view; and (o) craniomedial view. (p-r) cartilages with *E* = 1 MPa, in: (p) plantar view; (q) dorsal view; and (r) craniomedial view. (s-aa) Von Mises stress (MPa) distribution results for a subunguligrade morphotype, including: (s-u) cartilages with *E* = 100 MPa, in: (s) plantar view; (t) dorsal view; and (u) craniomedial view. (v-x) cartilages with *E* = 10 MPa, in: (v) plantar view; (w) dorsal view; and (x) craniomedial view. (y-aa) cartilages with *E* = 1 MPa, in: (y) plantar view; (z) dorsal view; and (aa) craniomedial view. Cold (blue) and warm (red) colours show lower and higher von Mises stresses respectively. Abbreviations: M-Di, Mid-Digitigrady; Di, Digitigrady; Sub, Subunguligrady; BC5, Boundary condition 5; C1–3, Cartilages conditions 1–3 (*E* values of 100 MPa, 10 MPa, and 1 Mpa, respectively); L1, Loading condition 1 (applied force of 10,000 N).

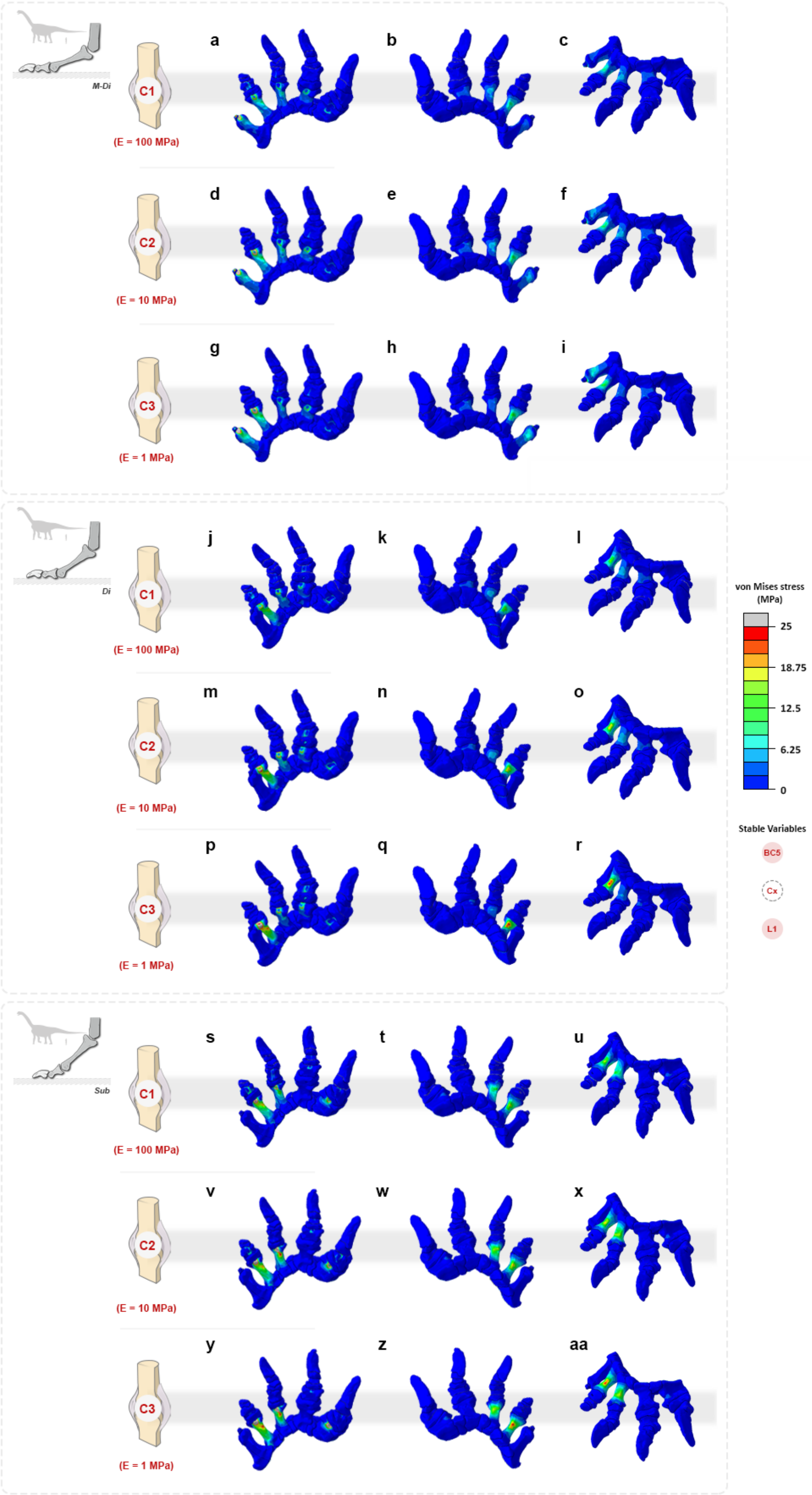

Fig. S16. Von Mises stress (MPa) distribution results for the sensitivity analysis of the cartilage properties (*E*) in *Camarasaurus* sp. (a-i) Von Mises stress (MPa) distribution results for a mid-digitigrade morphotype, including: (a-c) cartilages with *E* = 100 MPa, in: (a) plantar view; (b) dorsal view; and (c) craniomedial view. (d-f) cartilages with *E* = 10 MPa, in: (d) plantar view; (e) dorsal view; and (f) craniomedial view. (g-i) cartilages with *E* = 1 MPa, in: (g) plantar view; (h) dorsal view; and (i) craniomedial view. (j-r) Von Mises stress (MPa) distribution results for a digitigrade morphotype, including: (j-l) cartilages with *E* = 100 MPa, in: (j) plantar view; (k) dorsal view; and (l) craniomedial view. (m-o) cartilages with *E* = 10 MPa, in: (m) plantar view; (n) dorsal view; and (o) craniomedial view. (p-r) cartilages with *E* = 1 MPa, in: (p) plantar view; (q) dorsal view; and (r) craniomedial view. (s-aa) Von Mises stress (MPa) distribution results for a subunguligrade morphotype, including: (s-u) cartilages with *E* = 100 MPa, in: (s) plantar view; (t) dorsal view; and (u) craniomedial view. (v-x) cartilages with *E* = 10 MPa, in: (v) plantar view; (w) dorsal view; and (x) craniomedial view. (y-aa) cartilages with *E* = 1 MPa, in: (y) plantar view; (z) dorsal view; and (aa) craniomedial view. Cold (blue) and warm (red) colours show lower and higher von Mises stresses respectively. Abbreviations: M-Di, Mid-Digitigrady; Di, Digitigrady; Sub, Subunguligrady; BC5, Boundary condition 5; C1–3, Cartilages conditions 1–3 (*E* values of 100 MPa, 10 MPa, and 1 Mpa, respectively); L1, Loading condition 1 (applied force of 10,000 N).

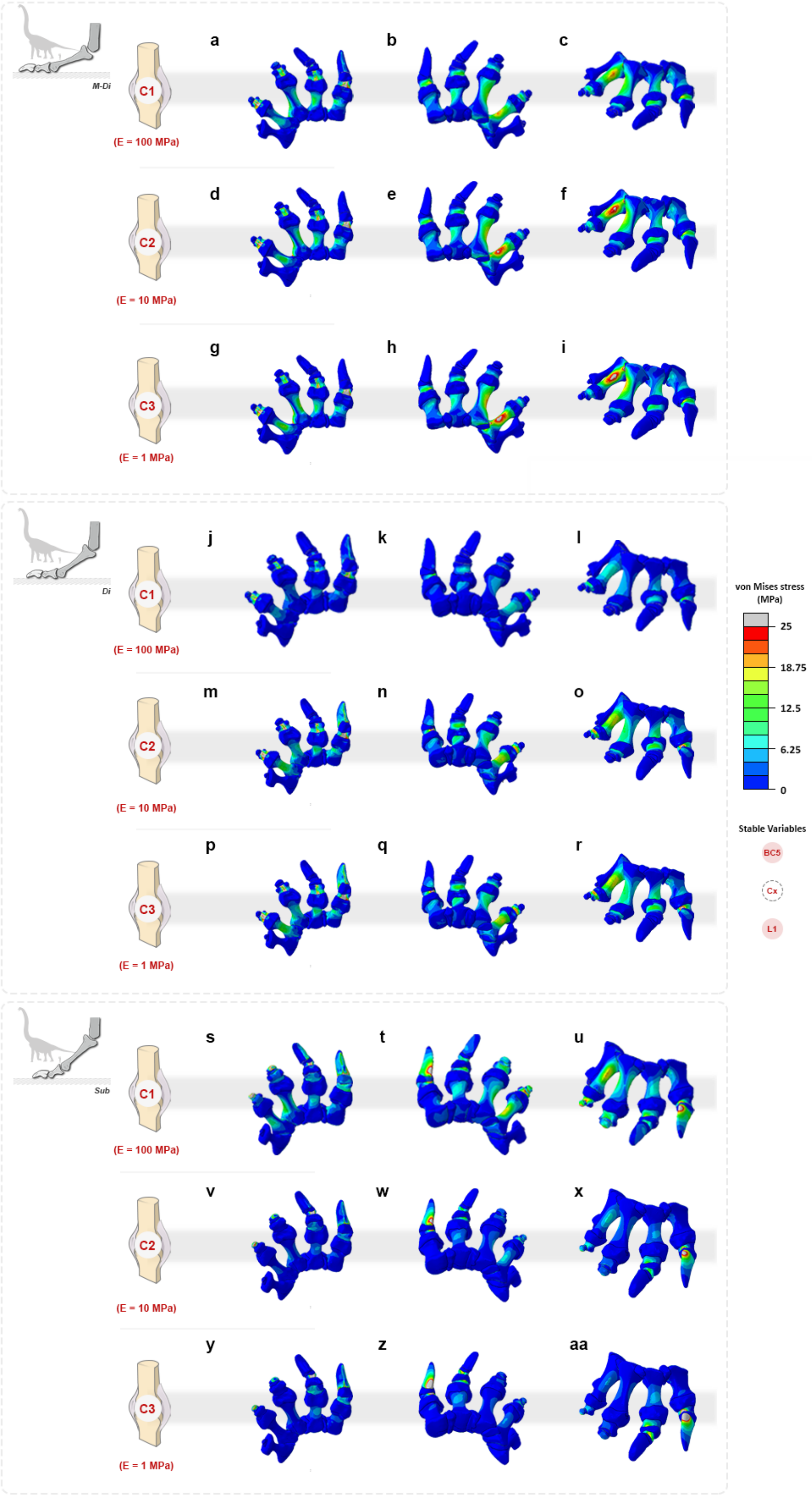

Fig. S17. Von Mises stress (MPa) distribution results for the sensitivity analysis of the cartilage properties (*E*) in *Giraffatitan brancai*. (a-i) Von Mises stress (MPa) distribution results for a mid-digitigrade morphotype, including: (a-c) cartilages with *E* = 100 MPa, in: (a) plantar view; (b) dorsal view; and (c) craniomedial view. (d-f) cartilages with *E* = 10 MPa, in: (d) plantar view; (e) dorsal view; and (f) craniomedial view. (g-i) cartilages with *E* = 1 MPa, in: (g) plantar view; (h) dorsal view; and (i) craniomedial view. (j-r) Von Mises stress (MPa) distribution results for a digitigrade morphotype, including: (j-l) cartilages with *E* = 100 MPa, in: (j) plantar view; (k) dorsal view; and (l) craniomedial view. (m-o) cartilages with *E* = 10 MPa, in: (m) plantar view; (n) dorsal view; and (o) craniomedial view. (p-r) cartilages with *E* = 1 MPa, in: (p) plantar view; (q) dorsal view; and (r) craniomedial view. (s-aa) Von Mises stress (MPa) distribution results for a subunguligrade morphotype, including: (s-u) cartilages with *E* = 100 MPa, in: (s) plantar view; (t) dorsal view; and (u) craniomedial view. (v-x) cartilages with *E* = 10 MPa, in: (v) plantar view; (w) dorsal view; and (x) craniomedial view. (y-aa) cartilages with *E* = 1 MPa, in: (y) plantar view; (z) dorsal view; and (aa) craniomedial view. Cold (blue) and warm (red) colours show lower and higher von Mises stresses respectively. Abbreviations: M-Di, Mid-Digitigrady; Di, Digitigrady; Sub, Subunguligrady; BC5, Boundary condition 5; C1–3, Cartilages conditions 1–3 (*E* values of 100 MPa, 10 MPa, and 1 Mpa, respectively); L1, Loading condition 1 (applied force of 10,000 N).

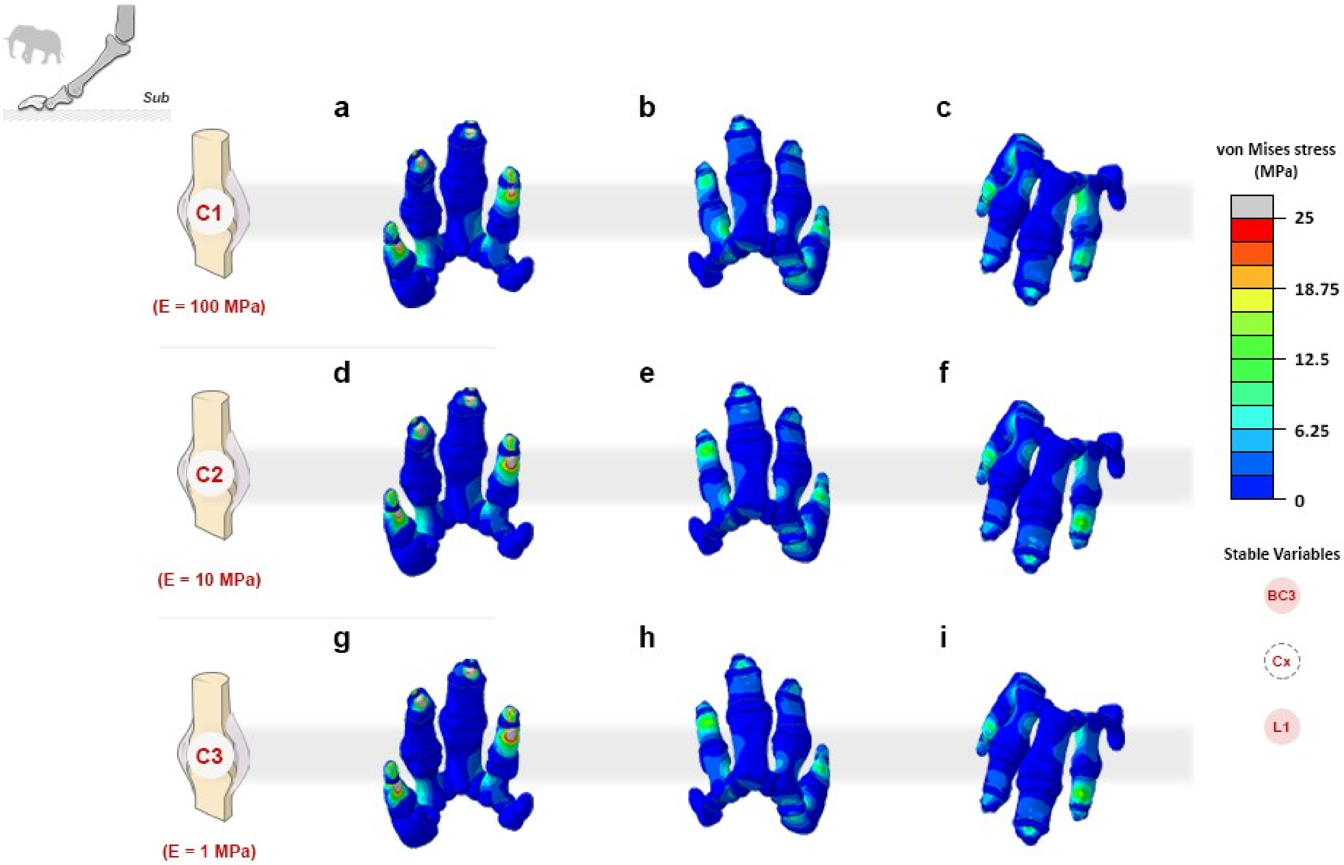

Fig. S18. Von Mises stress (MPa) distribution results for the sensitivity analysis of the cartilage properties (*E*) of the skeletal FEM without soft tissue pad for the simulated elephant pes. (a-c) Von Mises stress (MPa) distribution results for cartilages holding Young's modulus (*E*) value of 100 MPa, in: (a) plantar view; (b) dorsal view; and (c) craniomedial view. (d-f) Von Mises stress (MPa) distribution results for cartilages holding an *E* value of 10 MPa, in: (d) plantar view; (e) dorsal view; and (f) craniomedial view. (g-i) Von Mises stress (MPa) distribution results for cartilages holding an *E* value of 1 MPa, in: (g) plantar view; (h) dorsal view; and (i) craniomedial view. Cold (blue) and warm (red) colours show lower and higher von Mises stresses, respectively. Abbreviations: Sub, Subunguligrady, BC3, Boundary condition 3; C1–3, Cartilages conditions 1–3 (*E* values of 100 MPa, 10 MPa, and 1 MPa, respectively); L1, Loading condition 1 (applied force of 10,000 N).

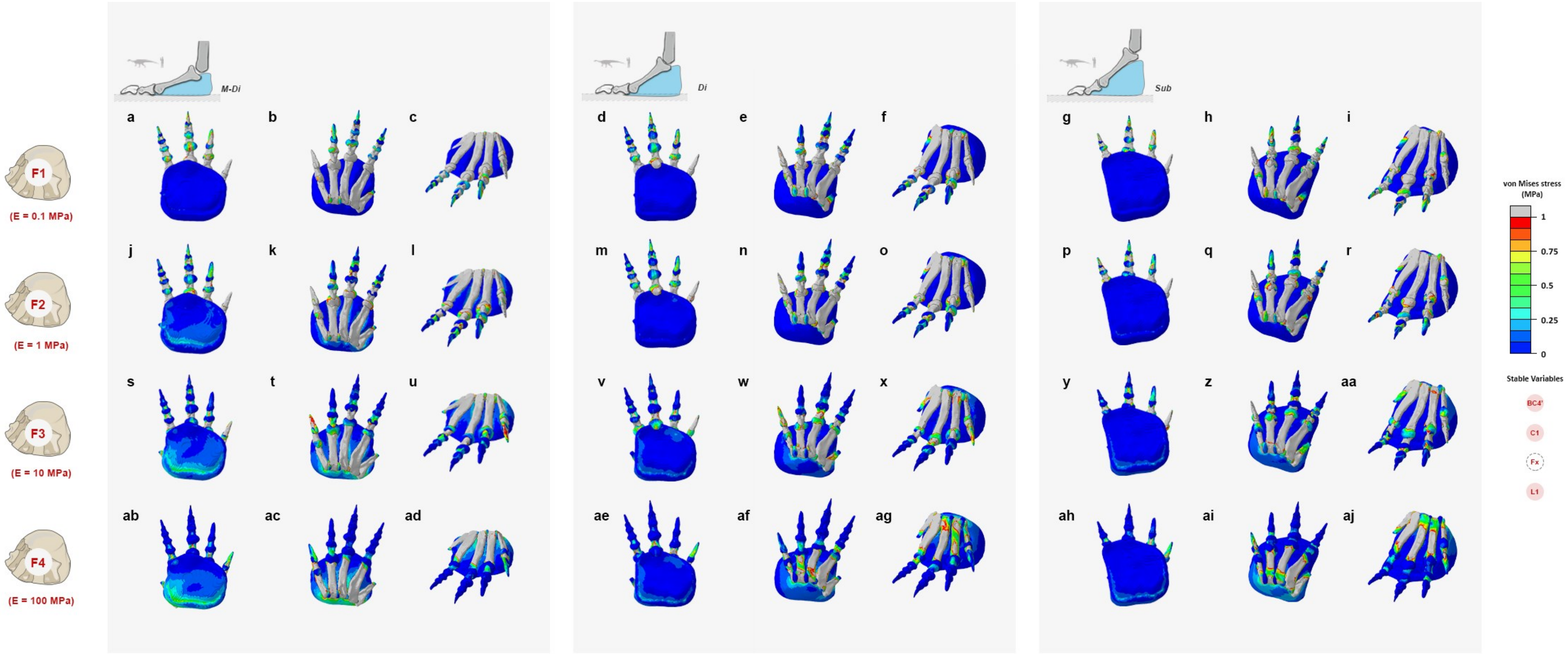

Fig. S19. Von Mises stress (MPa) distribution results for the sensitivity analysis of varying the Young's modulus € properties of the soft tissue pad to the mid-digitigrade (left), digitigrade (centre), and subunguligrade (right) pedal morphotypes in *Plateosaurus engelhardti*. (a-i) Von Mises stress (MPa) distribution results for all morphotypes for a soft tissue pad condition F1 involving an $E = 0.1$ MPa, in: (a, d, g) plantar view; (b, e, h) dorsal view; and (c, f, i) craniomedial view. (j-r) Von Mises stress (MPa) distribution results for all morphotypes for a soft tissue pad condition F2 involving an $E = 1$ MPa, in: (j, m, p) plantar view; (k, n, q) dorsal view; and (l, o, r) craniomedial view. (s-aa) Von Mises stress (MPa) distribution results for all morphotypes for a soft tissue pad condition F3 involving an $E = 10$ MPa, in: (s, v, y) plantar view; (t, w, z) dorsal view; and (u, x, aa) craniomedial view. (ab-aj) Von Mises stress (MPa) distribution results for all morphotypes for a soft tissue pad condition F4 involving an $E = 100$ MPa, in: (ab, ae, ah) plantar view; (ac, af, ai) dorsal view; and (ad, ag, aj) craniomedial view. Cold (blue) and warm (red) colours show lower and higher von Mises stresses, respectively. Abbreviations: M-Di, Mid-Digitigrady; Di, Digitigrady; Sub, Subunguligrady; BC4', Boundary condition 4'; C1, Cartilage condition 1 (E value of 100 Mpa); F1–4, Soft tissue pad conditions 1–4 (E values of 0.1 MPa, 1 MPa, 10 MPa, and 100 Mpa, respectively); L1, Loading condition 1 (applied forces of 10,000 N).

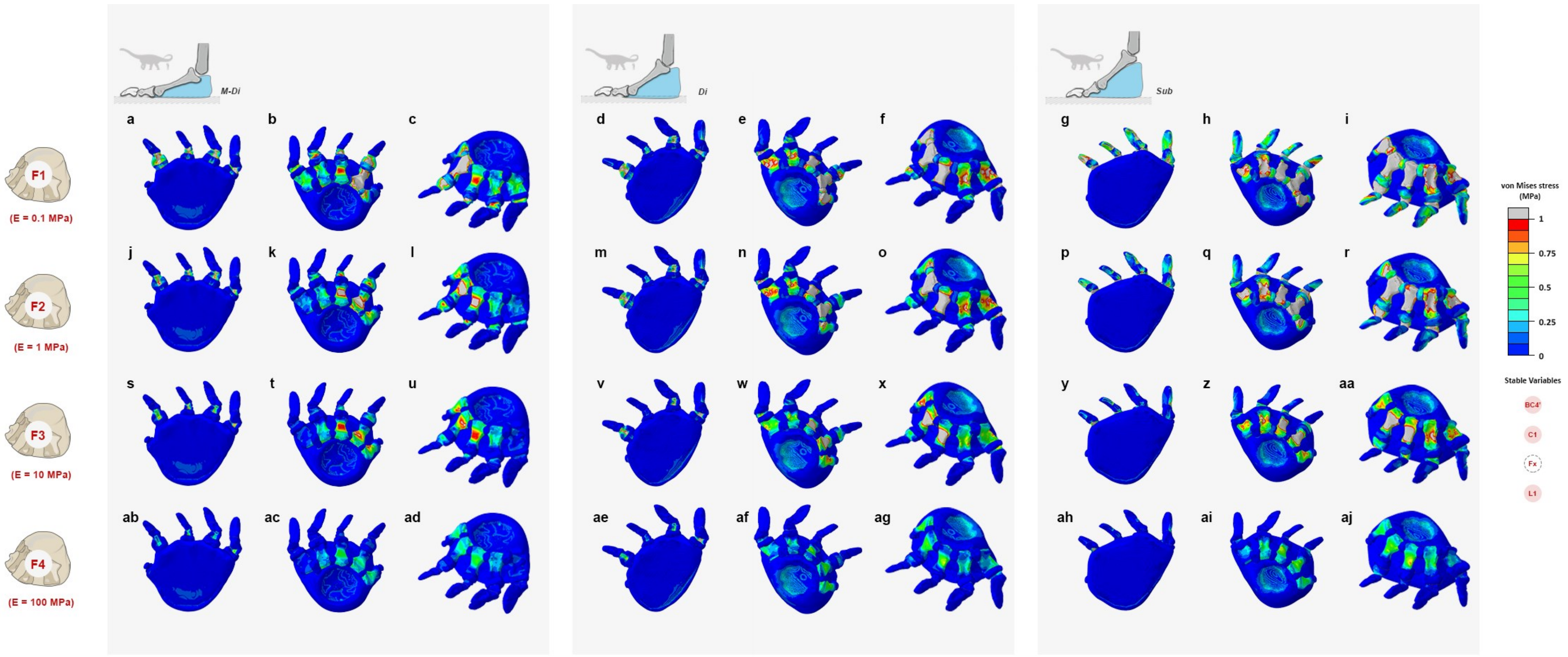

Fig. S20. Von Mises stress (MPa) distribution results for the sensitivity analysis of varying the Young's modulus (*E*) properties of the soft tissue pad to the mid-digitigrade (left), digitigrade (centre), and subunguligrade (right) pedal morphotypes in *Rhoetosaurus brownei*. (a-i) Von Mises stress (MPa) distribution results for all morphotypes for a soft tissue pad condition F1 involving an $E = 0.1$ MPa, in: (a, d, g) plantar view; (b, e, h) dorsal view; and (c, f, i) craniomedial view. (j-r) Von Mises stress (MPa) distribution results for all morphotypes for a soft tissue pad condition F2 involving an $E = 1$ MPa, in: (j, m, p) plantar view; (k, n, q) dorsal view; and (l, o, r) craniomedial view. (s-aa) Von Mises stress (MPa) distribution results for all morphotypes for a soft tissue pad condition F3 involving an $E = 10$ MPa, in: (s, v, y) plantar view; (t, w, z) dorsal view; and (u, x, aa) craniomedial view. (ab-aj) Von Mises stress (MPa) distribution results for all morphotypes for a soft tissue pad condition F4 involving an $E = 100$ MPa, in: (ab, ae, ah) plantar view; (ac, af, ai) dorsal view; and (ad, ag, aj) craniomedial view. Cold (blue) and warm (red) colours show lower and higher von Mises stresses, respectively. Abbreviations: M-Di, Mid-Digitigrady; Di, Digitigrady; Sub, Subunguligrady; BC4', Boundary condition 4'; C1, Cartilage condition 1 (*E* value of 100 Mpa); F1–4, Soft tissue pad conditions 1–4 (*E* values of 0.1 MPa, 1 MPa, 10 MPa, and 100 Mpa, respectively); L1, Loading condition 1 (applied forces of 10,000 N).

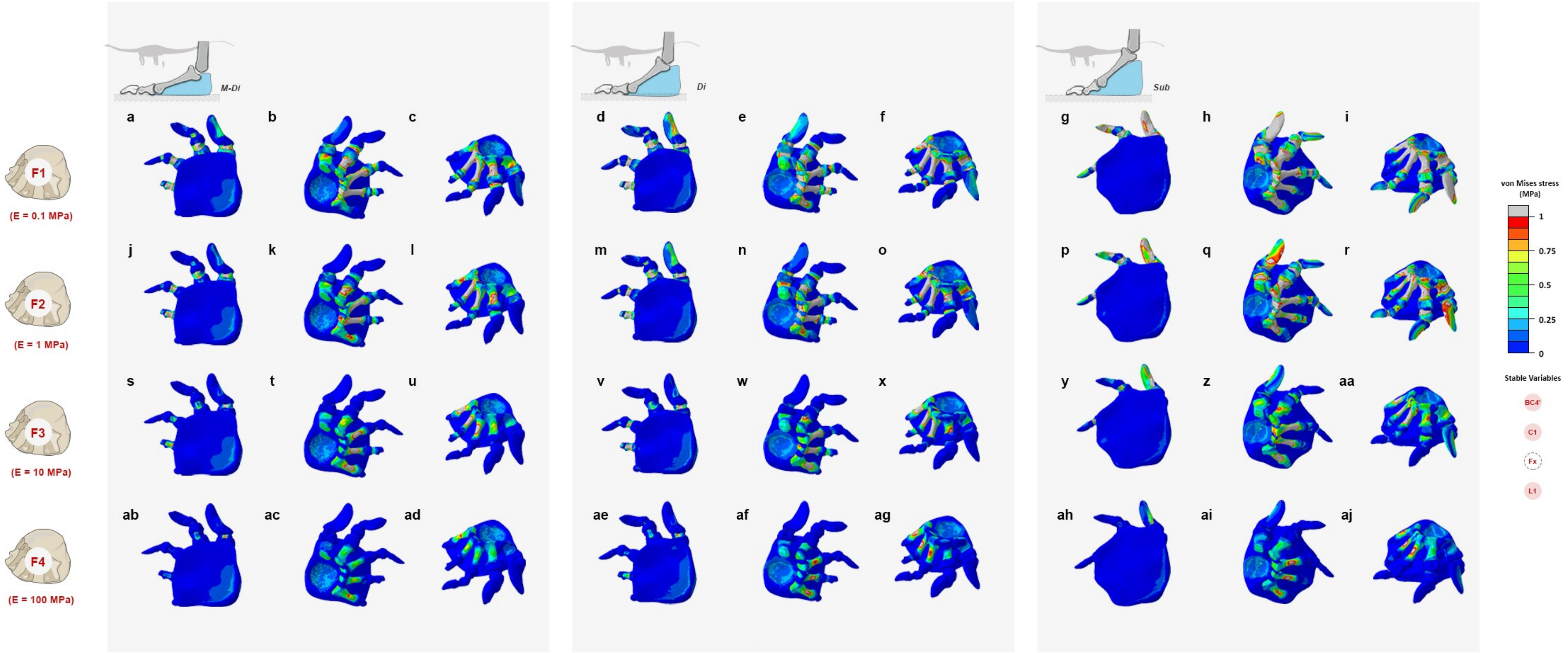

Fig. S21. Von Mises stress (MPa) distribution results for the sensitivity analysis of varying the Young's modulus (*E*) properties of the soft tissue pad to the mid-digitigrade (left), digitigrade (centre), and subunguligrade (right) pedal morphotypes in *Diplodocus carnegii*. (a-i) Von Mises stress (MPa) distribution results for all morphotypes for a soft tissue pad condition F1 involving an $E = 0.1$ MPa, in: (a, d, g) plantar view; (b, e, h) dorsal view; and (c, f, i) craniomedial view. (j-r) Von Mises stress (MPa) distribution results for all morphotypes for a soft tissue pad condition F2 involving an $E = 1$ MPa, in: (j, m, p) plantar view; (k, n, q) dorsal view; and (l, o, r) craniomedial view. (s-aa) Von Mises stress (MPa) distribution results for all morphotypes for a soft tissue pad condition F3 involving an $E = 10$ MPa, in: (s, v, y) plantar view; (t, w, z) dorsal view; and (u, x, aa) craniomedial view. (ab-aj) Von Mises stress (MPa) distribution results for all morphotypes for a soft tissue pad condition F4 involving an $E = 100$ MPa, in: (ab, ae, ah) plantar view; (ac, af, ai) dorsal view; and (ad, ag, aj) craniomedial view. Cold (blue) and warm (red) colours show lower and higher von Mises stresses, respectively. Abbreviations: M-Di, Mid-Digitigrady; Di, Digitigrady; Sub, Subunguligrady; BC4', Boundary condition 4'; C1, Cartilage condition 1 (*E* value of 100 Mpa); F1–4, Soft tissue pad conditions 1–4 (*E* values of 0.1 MPa, 1 MPa, 10 MPa, and 100 Mpa, respectively); L1, Loading condition 1 (applied forces of 10,000 N).

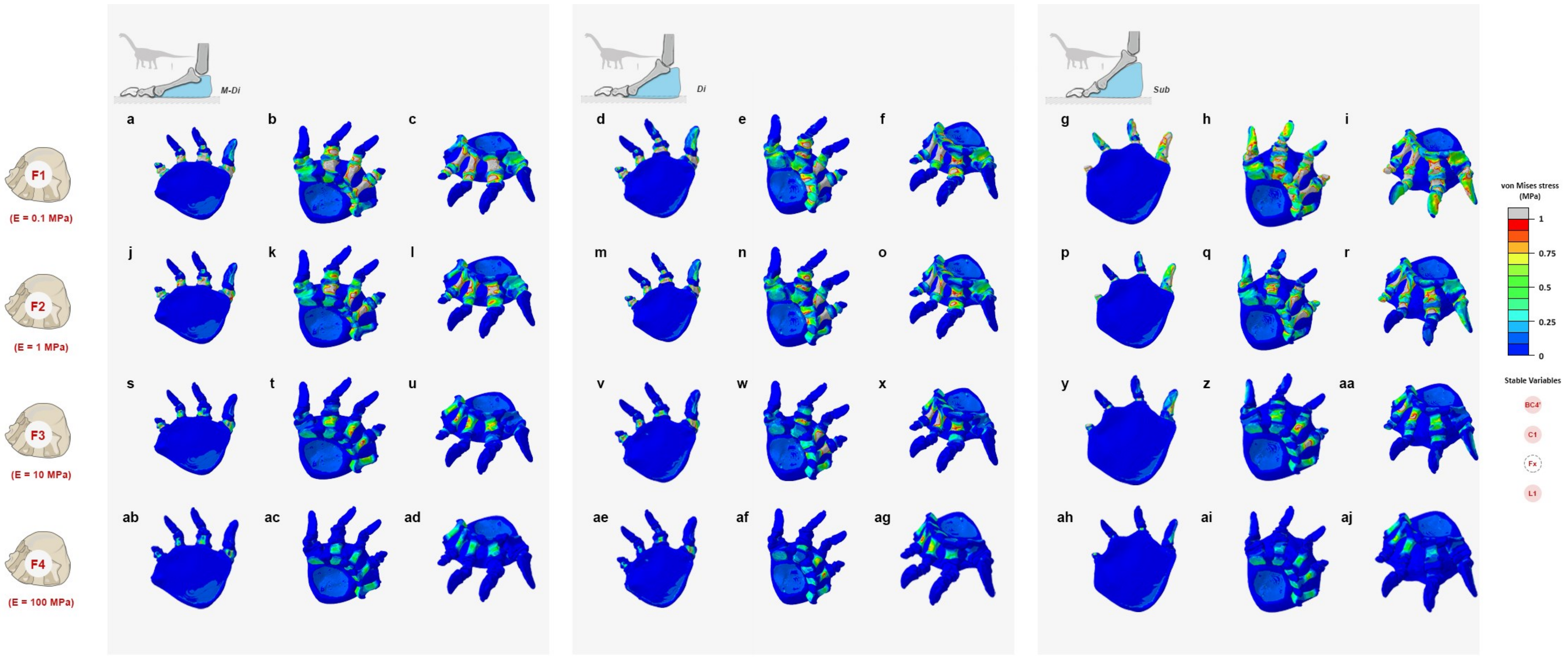

Fig. S22. Von Mises stress (MPa) distribution results for the sensitivity analysis of varying the Young's modulus (*E*) properties of the soft tissue pad to the mid-digitigrade (left), digitigrade (centre), and subunguligrade (right) pedal morphotypes in *Camarasaurus* sp. (a-i) Von Mises stress (MPa) distribution results for all morphotypes for a soft tissue pad condition F1 involving an *E* = 0.1 MPa, in: (a, d, g) plantar view; (b, e, h) dorsal view; and (c, f, i) craniomedial view. (j-r) Von Mises stress (MPa) distribution results for all morphotypes for a soft tissue pad condition F2 involving an *E* = 1 MPa, in: (j, m, p) plantar view; (k, n, q) dorsal view; and (l, o, r) craniomedial view. (s-aa) Von Mises stress (MPa) distribution results for all morphotypes for a soft tissue pad condition F3 involving an *E* = 10 MPa, in: (s, v, y) plantar view; (t, w, z) dorsal view; and (u, x, aa) craniomedial view. (ab-aj) Von Mises stress (MPa) distribution results for all morphotypes for a soft tissue pad condition F4 involving an *E* = 100 MPa, in: (ab, ae, ah) plantar view; (ac, af, ai) dorsal view; and (ad, ag, aj) craniomedial view. Cold (blue) and warm (red) colours show lower and higher von Mises stresses, respectively. Abbreviations: M-Di, Mid-Digitigrady; Di, Digitigrady; Sub, Subunguligrady; BC4', Boundary condition 4'; C1, Cartilage condition 1 (*E* value of 100 Mpa); F1–4, Soft tissue pad conditions 1–4 (*E* values of 0.1 MPa, 1 MPa, 10 MPa, and 100 Mpa, respectively); L1, Loading condition 1 (applied forces of 10,000 N).

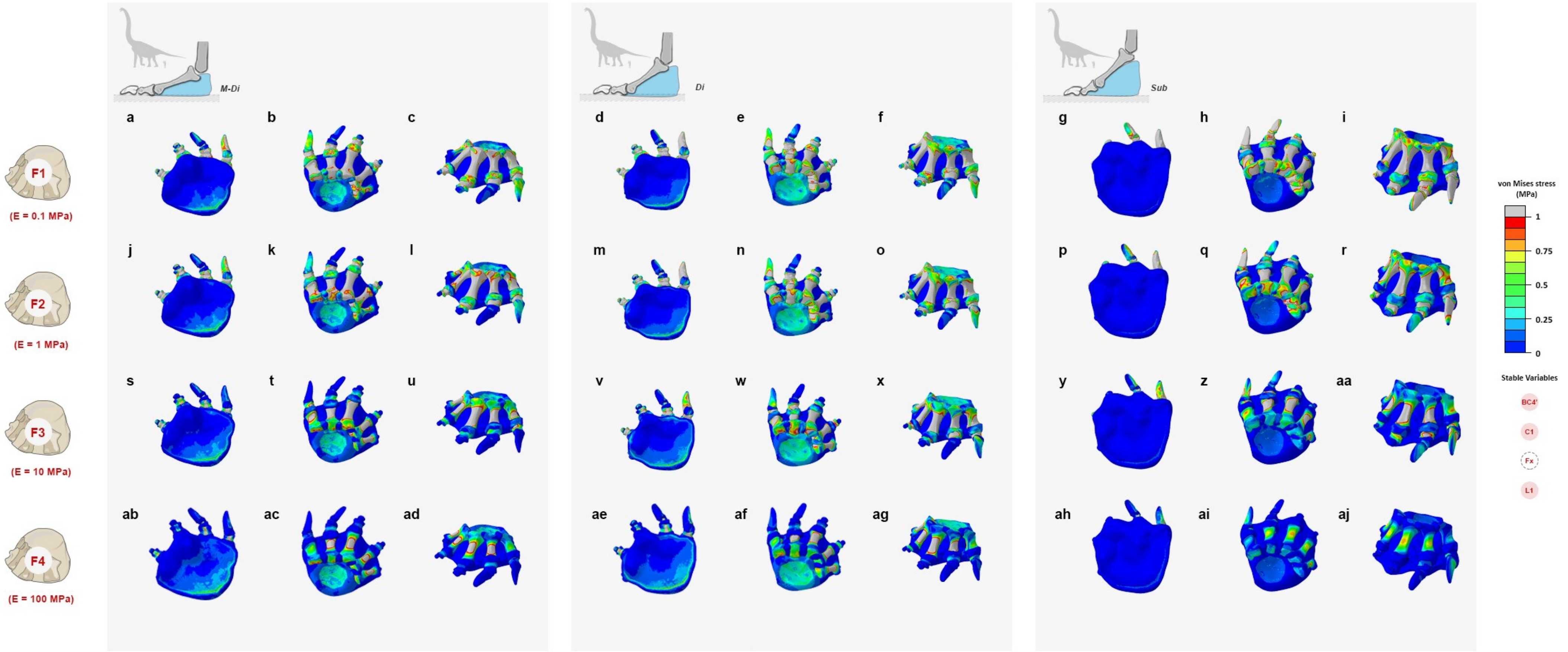

Fig. S23. Von Mises stress (MPa) distribution results for the sensitivity analysis of varying the Young's modulus (*E*) properties of the soft tissue pad to the mid-digitigrade (left), digitigrade (centre), and subunguligrade (right) pedal morphotypes in *Giraffatitan brancai*. (a-i) Von Mises stress (MPa) distribution results for all morphotypes for a soft tissue pad condition F1 involving an *E* = 0.1 MPa, in: (a, d, g) plantar view; (b, e, h) dorsal view; and (c, f, i) craniomedial view. (j-r) Von Mises stress (MPa) distribution results for all morphotypes for a soft tissue pad condition F2 involving an *E* = 1 MPa, in: (j, m, p) plantar view; (k, n, q) dorsal view; and (l, o, r) craniomedial view. (s-aa) Von Mises stress (MPa) distribution results for all morphotypes for a soft tissue pad condition F3 involving an *E* = 10 MPa, in: (s, v, y) plantar view; (t, w, z) dorsal view; and (u, x, aa) craniomedial view. (ab-aj) Von Mises stress (MPa) distribution results for all morphotypes for a soft tissue pad condition F4 involving an *E* = 100 MPa, in: (ab, ae, ah) plantar view; (ac, af, ai) dorsal view; and (ad, ag, aj) craniomedial view. Cold (blue) and warm (red) colours show lower and higher von Mises stresses, respectively. Abbreviations: M-Di, Mid-Digitigrady; Di, Digitigrady; Sub, Subunguligrady; BC4', Boundary condition 4'; C1, Cartilage condition 1 (*E* value of 100 Mpa); F1–4, Soft tissue pad conditions 1–4 (*E* values of 0.1 MPa, 1 MPa, 10 MPa, and 100 Mpa, respectively); L1, Loading condition 1 (applied forces of 10,000 N).

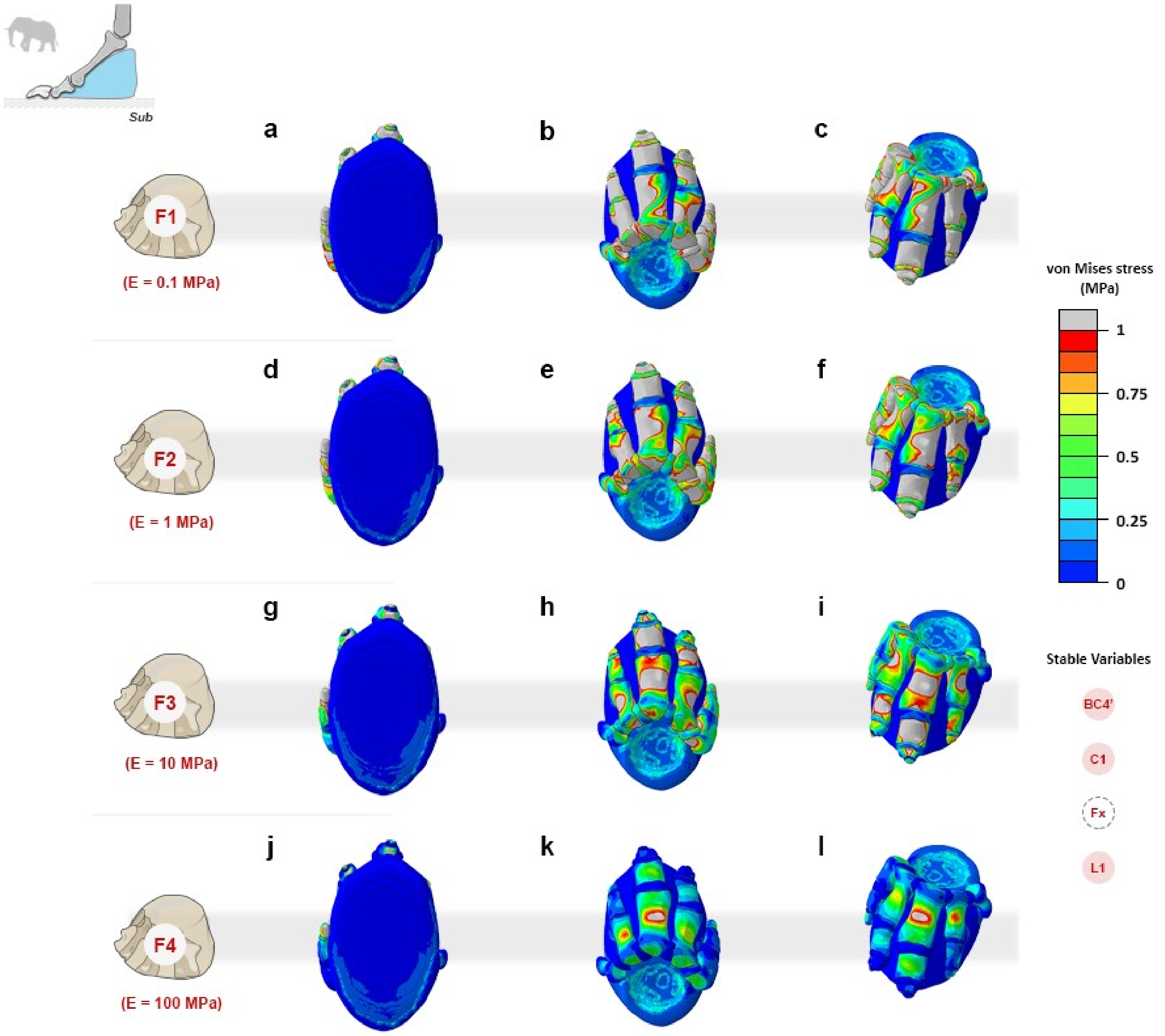

Fig. S24. Von Mises stress (MPa) distribution results the sensitivity analysis of varying the Young's modulus (*E*) properties of the soft tissue pad in the FEM of a simulated elephant pes. (a-c) Von Mises stress (MPa) distribution results for a soft tissue pad condition F1 involving an *E* = 0.1 MPa, in: (a) plantar view; (b) dorsal view; and (c) craniomedial view. (d-f) Von Mises stress (MPa) distribution results for a soft tissue pad condition F2 involving an *E* = 1 MPa, in: (d) plantar view; (e) dorsal view; and (f) craniomedial view. (g-i) Von Mises stress (MPa) distribution results for a soft tissue pad condition F3 involving an *E* = 10 MPa, in: (g) plantar view; (h) dorsal view; and (i) craniomedial view. (j-l) Von Mises stress (MPa) distribution results for a soft tissue pad condition F4 involving an *E* = 100 MPa, in: (j) plantar view; (k) dorsal view; and (l) craniomedial view. Cold (blue) and warm (red) colours show lower, and higher von Mises stresses, respectively. Abbreviations: Sub, Subunguligrady; BC4', Boundary condition 4'; C1, Cartilage condition 1 (*E* value of 100 Mpa); F1–4, Soft tissue pad conditions 1–4 (*E* values of 0.1 MPa, 1 MPa, 10 MPa, and 100 Mpa, respectively); L1, Loading condition 1 (applied forces of 10,000 N).

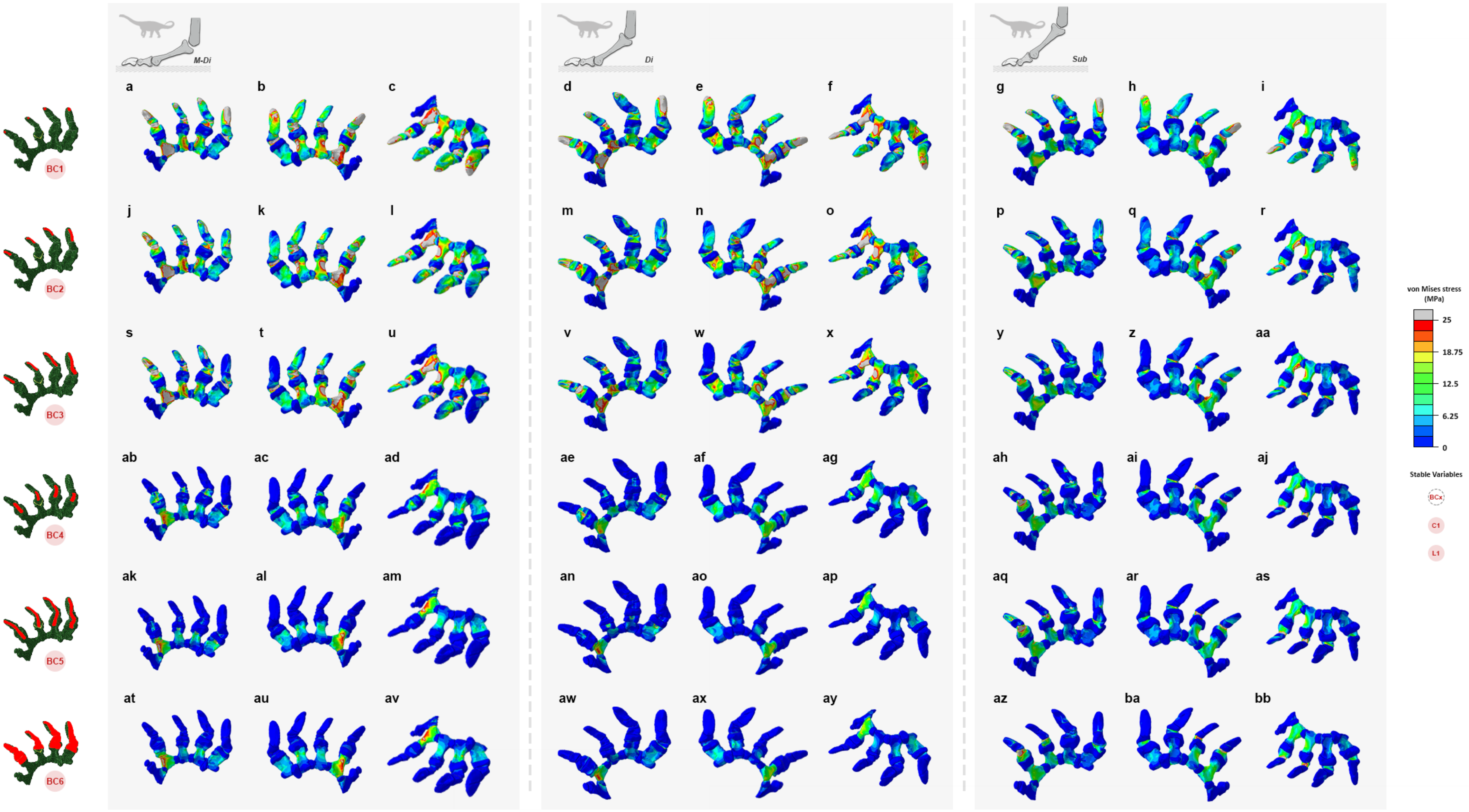

(legend on next page)

Fig. S25. Von Mises stress (MPa) distribution results for the sensitivity analysis of the six boundary conditions applied to the skeletally mid-digitigrade (left), digitigrade (centre), and subunguligrade (right) pedal morphotypes in *Rhoetosaurus brownei*. (a-i) Von Mises stress (MPa) distribution results for all morphotypes at the Boundary condition 1 (i.e., nodes constrained on a small portion at the tip of the distalmost phalanx), in: (a, d, g) plantar view; (b, e, h) dorsal view; and (c, f, i) craniomedial view. (j-r) Von Mises stress (MPa) distribution results for all morphotypes at the Boundary condition 2 (i.e., nodes constrained on the plantar surfaces of each distal phalanx only), in: (j, m, p) plantar view; (k, n, q) dorsal view; and (l, o, r) craniomedial view. (s-aa) Von Mises stress (MPa) distribution results for all morphotypes at the Boundary condition 3 (i.e., nodes constrained on the plantar surfaces of the IP joints), in: (s, v, y) plantar view; (t, w, z) dorsal view; and (u, x, aa) craniomedial view. (ab-aj) Von Mises stress (MPa) distribution results for all morphotypes at the Boundary condition 4 (i.e., nodes constrained on the plantar surfaces of the mid-portion of the pes only), in: (ab, ae, ah) plantar view; (ac, af, ai) dorsal view; and (ad, ag, aj) craniomedial view. (ak-as) Von Mises stress (MPa) distribution results for all morphotypes at the Boundary condition 5 (i.e., nodes constrained on a portion of the plantar surfaces of all bony elements in assumed contact with the ground), in: (ak, an, aq) plantar view; (al, ao, ar) dorsal view; and (am, ap, as) craniomedial view. (at-bb) Von Mises stress (MPa) distribution results for all morphotypes at the Boundary condition 6 (i.e., nodes constrained on the complete plantar surfaces of all bony elements in assumed contact with the ground), in: (at, aw, az) plantar view; (au, ax, ba) dorsal view; and (av, ay, bb) craniomedial view. Cold (blue) and warm (red) colours show lower, and higher von Mises stresses, respectively. Abbreviations: M-Di, Mid-Digitigrady; Di, Digitigrady; Sub, Subunguligrady; BC1–6, Boundary conditions 1 to 6, respectively; C1, Cartilage condition 1 (*E* value of 100 Mpa); L1, Loading condition 1 (applied forces of 10,000 N).

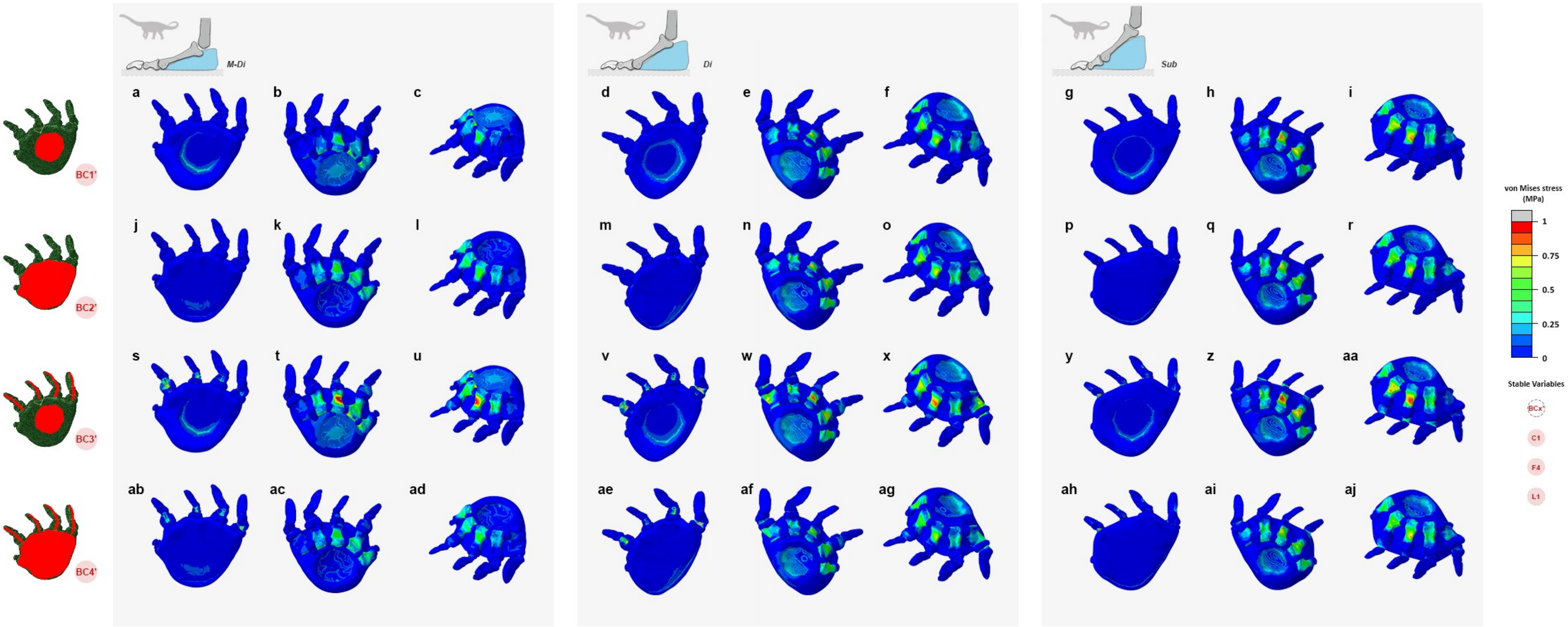

Fig. S26. Von Mises stress (MPa) distribution results for the sensitivity analysis of the four boundary conditions applied to the skeletally mid-digitigrade (left), digitigrade (centre), and subunguligrade (right) pedal morphotypes with a hypothetical soft tissue pad in *Rhoetosaurus brownei*. (a-i) Von Mises stress (MPa) distribution results for all morphotypes at the Boundary condition 1' (i.e., nodes constrained on the central portion of the plantar surface of the virtual pad in assumed contact with the ground), in: (a, d, g) plantar view; (b, e, h) dorsal view; and (c, f, i) craniomedial view. (j-r) Von Mises stress (MPa) distribution results for all morphotypes at the Boundary condition 2' (i.e., nodes constrained on the full plantar surface of the virtual pad in assumed contact with the ground), in: (j, m, p) plantar view; (k, n, q) dorsal view; and (l, o, r) craniomedial view. (s-aa) Von Mises stress (MPa) distribution results for all morphotypes at the Boundary condition 3' (i.e., nodes constrained on the central portion of the plantar surfaces of both the virtual pad and of all digits), in: (s, v, y) plantar view; (t, w, z) dorsal view; and (u, x, aa) craniomedial view. (ab-aj) Von Mises stress (MPa) distribution results for all morphotypes at the Boundary condition 4' (i.e., nodes constrained on the full plantar surfaces of both the virtual pad and of all digits), in: (ab, ae, ah) plantar view; (ac, af, ai) dorsal view; and (ad, ag, aj) craniomedial view. Cold (blue) and warm (red) colours show lower and higher von Mises stresses, respectively. Abbreviations: M-Di, Mid-Digitigrady; Di, Digitigrady; Sub, Subunguligrady; BC1'–4', Boundary conditions 1' to 4', respectively; C1, Cartilage condition 1 (*E* value of 100 Mpa); F4, Soft tissue pad conditions 4 (*E* values of 100 Mpa); L1, Loading condition 1 (applied forces of 10,000 N).

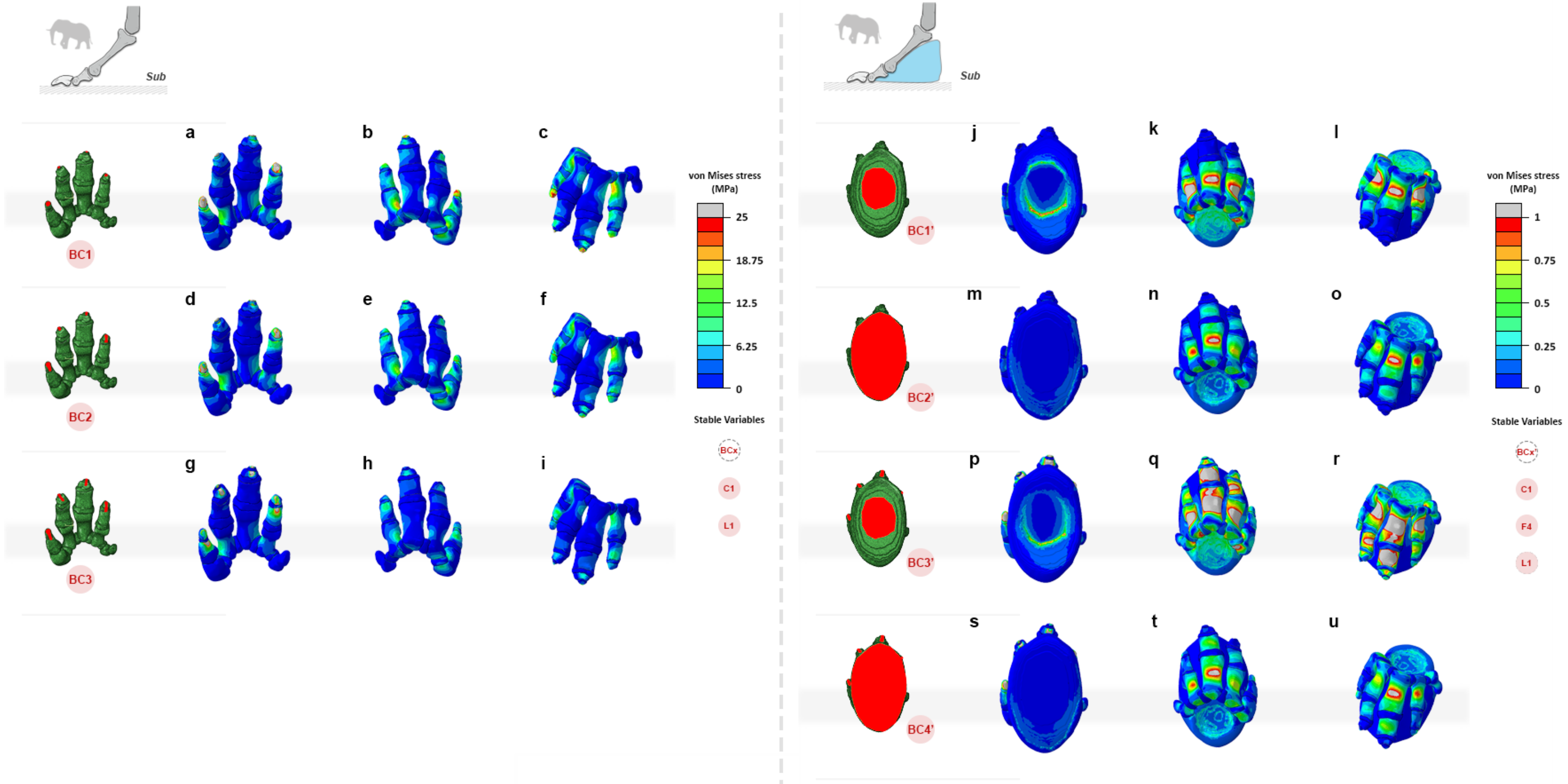

Fig. S27. Von Mises stress (MPa) distribution results for the sensitivity analysis of the boundary conditions applied to the skeletal FEM without (left) and with a hypothetical soft tissue pad (right) for a simulated elephant pes. (a-i) Von Mises stress (MPa) distribution results for the skeletal FEM without soft tissue pad at: (a-c) the Boundary condition 1 (i.e., nodes constrained on a small portion at the tip of the distalmost phalanx), in: (a) plantar view; (b) dorsal view; and (c) craniomedial view. (d-f) the Boundary condition 2 (i.e., nodes constrained on the plantar surfaces of each distal phalanx only), in: (d) plantar view; (e) dorsal view; and (f) craniomedial view. (g-i) the Boundary condition 3 (i.e., nodes constrained on the plantar surfaces of the IP joints), in: (g) plantar view; (h) dorsal view; and (i) craniomedial view. (j-u) Von Mises stress (MPa) distribution results for the skeletal FEM with soft tissue pad at: (j-l) the Boundary condition 1' (i.e., nodes constrained on the central portion of the plantar surface of the virtual pad in assumed contact with the ground), in: (j) plantar view; (k) dorsal view; and (l) craniomedial view. (m-o) the Boundary condition 2' (i.e., nodes constrained on the full plantar surface of the virtual pad in assumed contact with the ground), in: (m) plantar view; (n) dorsal view; and (o) craniomedial view. (p-r) the Boundary condition 3' (i.e., nodes constrained on the central portion of the plantar surfaces of both the virtual pad and of all digits), in: (p) plantar view; (q) dorsal view; and (r) craniomedial view. (s-u) the Boundary condition 4' (i.e., nodes constrained on the full plantar surfaces of both the virtual pad and of all digits), in: (s) plantar view; (t) dorsal view; and (u) craniomedial view. Cold (blue) and warm (red) colours show lower and higher von Mises stresses, respectively. Abbreviations: M-Di, Mid-Digitigrady; Di, Digitigrady; Sub, Subunguligrady; BCs1-3 and 1'–4', Boundary conditions 1 to 3 and 1' to 4', respectively; C1, Cartilage condition 1 (*E* value of 100 Mpa); F4, Soft tissue pad conditions 4 (*E* values of 100 Mpa); L1, Loading condition 1 (applied forces of 10,000 N).

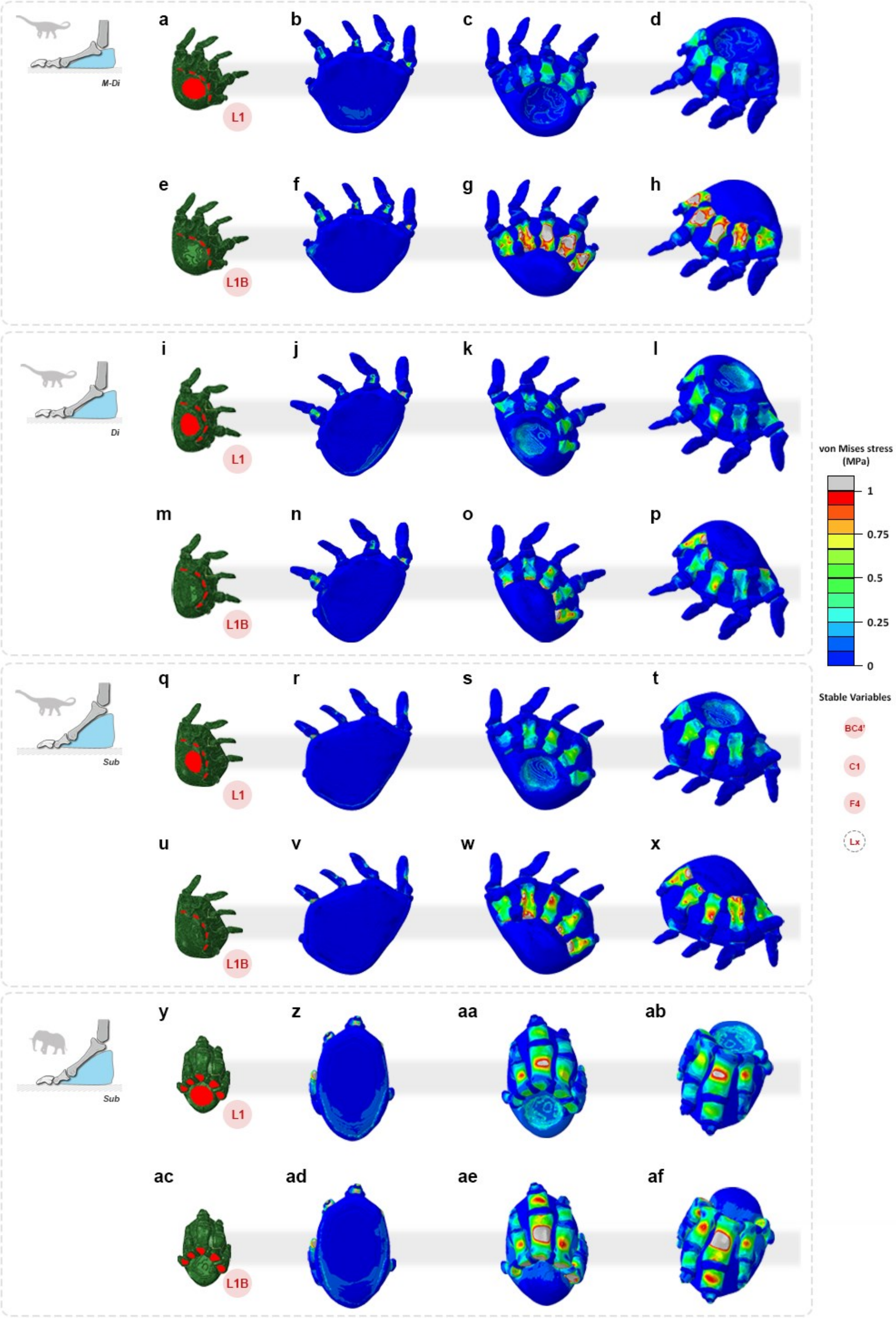

(legend on next page)

Fig. S28. Von Mises stress (MPa) distribution results for the sensitivity analysis of loading conditions in the FEMs with virtual pads of *Rhoetosaurus brownei* and a simulated elephant pes. (a-x) Von Mises stress (MPa) distribution results for the different postural morphotype of *Rhoetosaurus brownei*, with: (a-h) a mid-digitigrade morphotype, including (a-d) applied loads on the proximal end of the metatarsals and the soft tissue pad, in: (b) plantar view; (c) dorsal view; and (d) craniomedial view; (e-h) applied loads on the proximal end of the metatarsals only, in: (f) plantar view; (g) dorsal view; and (h) craniomedial view. (i-p) a digitigrade morphotype, including (i-l) applied loads on the proximal end of the metatarsals and the soft tissue pad, in: (j) plantar view; (k) dorsal view; and (l) craniomedial view; (m-p) applied loads on the proximal end of the metatarsals only, in: (n) plantar view; (o) dorsal view; and (p) craniomedial view. (q-x) a subunguligrade morphotype, including (q-t) applied loads on the proximal end of the metatarsals and the soft tissue pad, in: (r) plantar view; (s) dorsal view; and (t) craniomedial view; (u-x) applied loads on the proximal end of the metatarsals only, in: (v) plantar view; (w) dorsal view; and (x) craniomedial view. (z-af) Von Mises stress (MPa) distribution results for the subunguligrade morphotype of a simulated elephant pes, including: (y-ab) applied loads on the proximal end of the metatarsals and the soft tissue pad, in: (z) plantar view; (aa) dorsal view; and (ab) craniomedial view; (ac-af) applied loads on the proximal end of the metatarsals only, in: (ad) plantar view; (ae) dorsal view; and (af) craniomedial view. Cold (blue) and warm (red) colours show lower and higher von Mises stresses, respectively. Abbreviations: M-Di, Mid-Digitigrady; Di, Digitigrady; Sub, Subunguligrady; BC4', Boundary condition 4'; C1, Cartilage condition 1 (*E* value of 100 Mpa); F4, Soft tissue pad conditions 4 (*E* values of 100 Mpa); L1, Loading condition 1, involving an applied force of 10,000 N on the proximal end of the metatarsals and the soft tissue pad; L1B, Loading condition 1B, involving an applied force of 10,000 N on the proximal end of the metatarsals only.

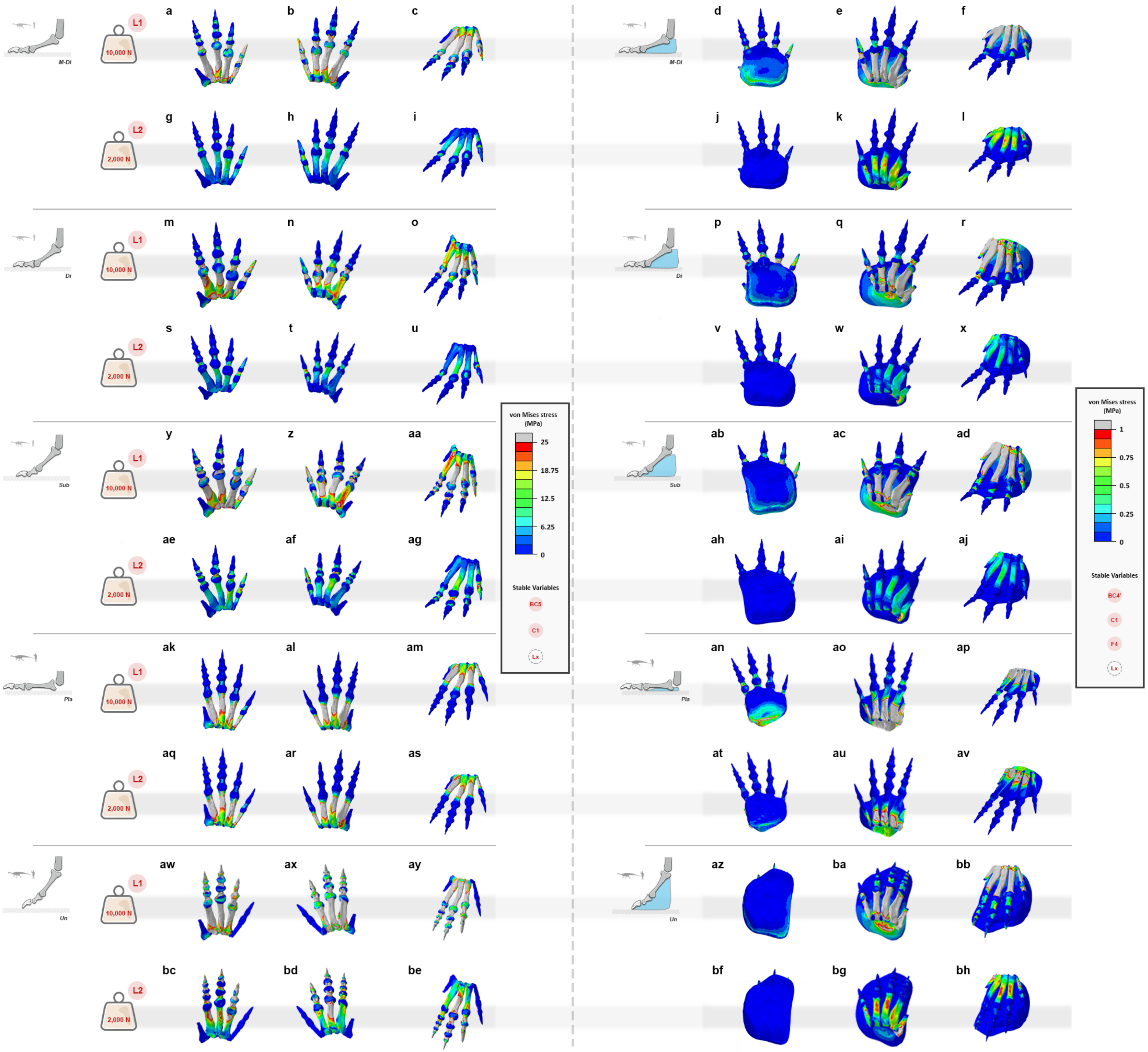

(legend on next page)

Fig. S29. Von Mises stress (MPa) distribution results for the sensitivity analysis between applied loads (proxy L1 vs physiologically realistic L2) in all skeletal morphotypes without (left) and with a hypothetical soft tissue pad (right) for *Plateosaurus engelhardti*. (a-l) Von Mises stress (MPa) distribution results for a mid-digitigrade morphotype under: (a-f) a proxy load L1, in: (a, d) plantar view; (b, e) dorsal view; and (c, f) craniomedial view. (g-l) a physiologically realistic load L2, in: (g, j) plantar view; (h, k) dorsal view; and (i, l) craniomedial view. (m-x) Von Mises stress (MPa) distribution results for a digitigrade morphotype under: (m-r) a proxy load L1, in: (m, p) plantar view; (n, q) dorsal view; and (o, r) craniomedial view. (s-x) a physiologically realistic load L2, in: (s, v) plantar view; (t, w) dorsal view; and (u, x) craniomedial view. (y-aj) Von Mises stress (MPa) distribution results for a subunguligrade morphotype under: (y-ad) a proxy load L1, in: (y, ab) plantar view; (z, ac) dorsal view; and (aa, ad) craniomedial view. (ae-aj) a physiologically realistic load L2, in: (ae, ah) plantar view; (af, ai) dorsal view; and (ag, aj) craniomedial view. (ak-av) Von Mises stress (MPa) distribution results for a plantigrade morphotype under: (ak-ap) a proxy load L1, in: (ak, an) plantar view; (al, ao) dorsal view; and (am, ap) craniomedial view. (aq-av) a physiologically realistic load L2, in: (aq, at) plantar view; (ar, au) dorsal view; and (as, av) craniomedial view. (aw-bh) Von Mises stress (MPa) distribution results for an unguligrade morphotype under: (aw-bb) a proxy load L1, in: (aw, az) plantar view; (ax, ba) dorsal view; and (ay, bb) craniomedial view. (bc-bh) a physiologically realistic load L2, in: (bc, bf) plantar view; (bd, bg) dorsal view; and (be, bh) craniomedial view. Cold (blue) and warm (red) colours show lower and higher von Mises stresses, respectively. Abbreviations: M-Di, Mid-Digitigrady; Di, Digitigrady; Sub, Subunguligrady; Pla, Plantigrady; Un, Unguligrady; BC5 & BC4', Boundary conditions 5 and 4', respectively; C1, Cartilage condition 1 (*E* value of 100 Mpa); F4, Soft tissue pad condition 4 (*E* value of 100 Mpa); L1, proxy load condition 1 (applied force of 10,000 N); L2, physiologically realistic load for *Plateosaurus engelhardti* (applied force of 2,000 N). Note: distinct scales of von Mises stresses used between left and right subdivisions.

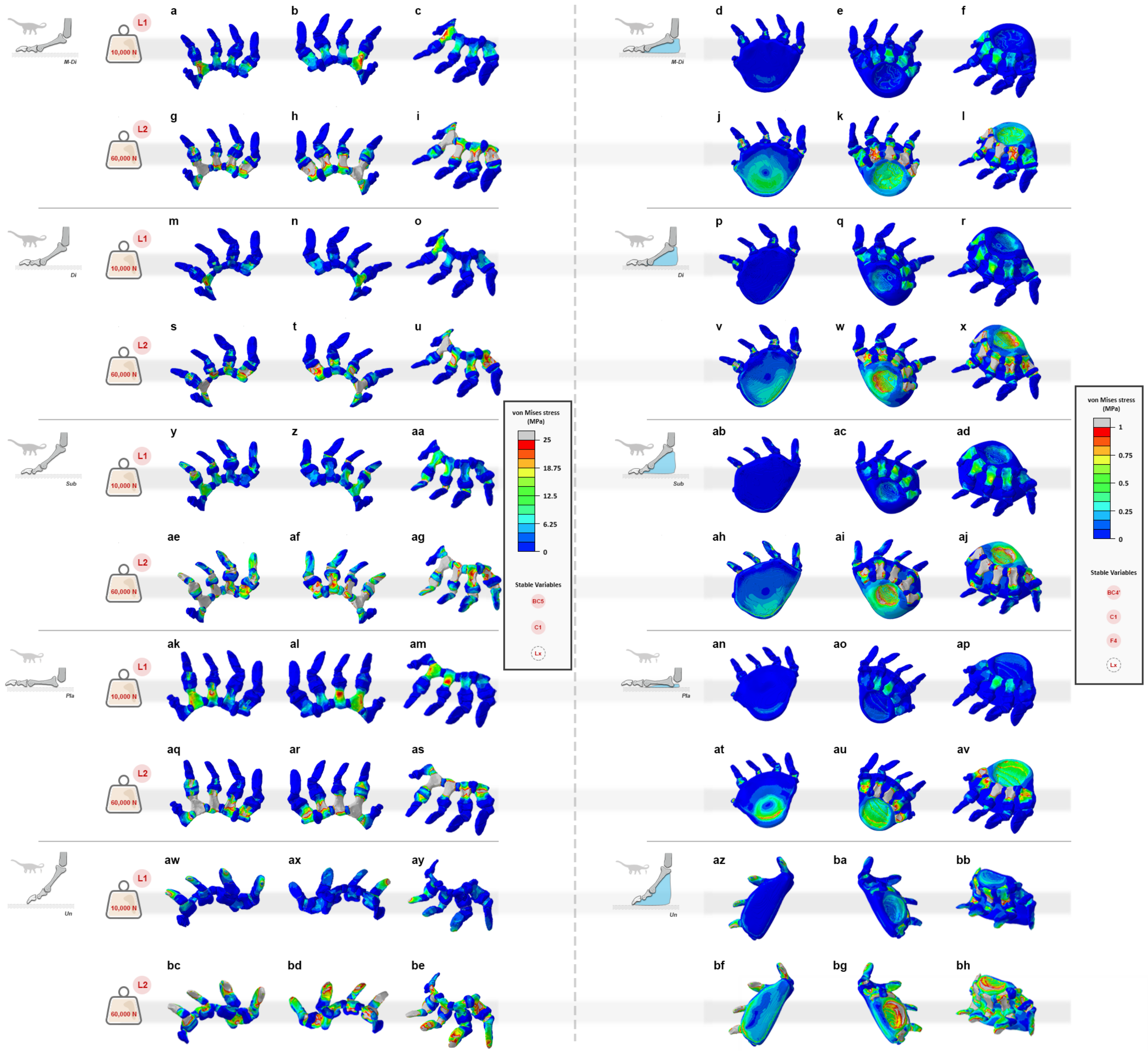

(legend on next page)

Fig. S30. Von Mises stress (MPa) distribution results for the sensitivity analysis between applied loads (proxy L1 vs physiologically realistic L2) in all skeletal morphotypes without (left) and with a hypothetical soft tissue pad (right) for *Rhoetosaurus brownei*. (a-l) Von Mises stress (MPa) distribution results for a mid-digitigrade morphotype under: (a-f) a proxy load L1, in: (a, d) plantar view; (b, e) dorsal view; and (c, f) craniomedial view. (g-l) a physiologically realistic load L2, in: (g, j) plantar view; (h, k) dorsal view; and (i, l) craniomedial view. (m-x) Von Mises stress (MPa) distribution results for a digitigrade morphotype under: (m-r) a proxy load L1, in: (m, p) plantar view; (n, q) dorsal view; and (o, r) craniomedial view. (s-x) a physiologically realistic load L2, in: (s, v) plantar view; (t, w) dorsal view; and (u, x) craniomedial view. (y-aj) Von Mises stress (MPa) distribution results for a subunguligrade morphotype under: (y-ad) a proxy load L1, in: (y, ab) plantar view; (z, ac) dorsal view; and (aa, ad) craniomedial view. (ae-aj) a physiologically realistic load L2, in: (ae, ah) plantar view; (af, ai) dorsal view; and (ag, aj) craniomedial view. (ak-av) Von Mises stress (MPa) distribution results for a plantigrade morphotype under: (ak-ap) a proxy load L1, in: (ak, an) plantar view; (al, ao) dorsal view; and (am, ap) craniomedial view. (aq-av) a physiologically realistic load L2, in: (aq, at) plantar view; (ar, au) dorsal view; and (as, av) craniomedial view. (aw-bh) Von Mises stress (MPa) distribution results for an unguligrade morphotype under: (aw-bb) a proxy load L1, in: (aw, az) plantar view; (ax, ba) dorsal view; and (ay, bb) craniomedial view. (bc-bh) a physiologically realistic load L2, in: (bc, bf) plantar view; (bd, bg) dorsal view; and (be, bh) craniomedial view. Cold (blue) and warm (red) colours show lower and higher von Mises stresses, respectively. Abbreviations: M-Di, Mid-Digitigrady; Di, Digitigrady; Sub, Subunguligrady; Pla, Plantigrady; Un, Unguligrady; BC5 & BC4', Boundary conditions 5 and 4', respectively; C1, Cartilage condition 1 (*E* value of 100 Mpa); F4, Soft tissue pad condition 4 (*E* value of 100 Mpa); L1, proxy load condition 1 (applied force of 10,000 N); L2, physiologically realistic load for *Rhoetosaurus brownei* (applied force of 60,000 N). Note: distinct scales of von Mises stresses used between left and right subdivisions.

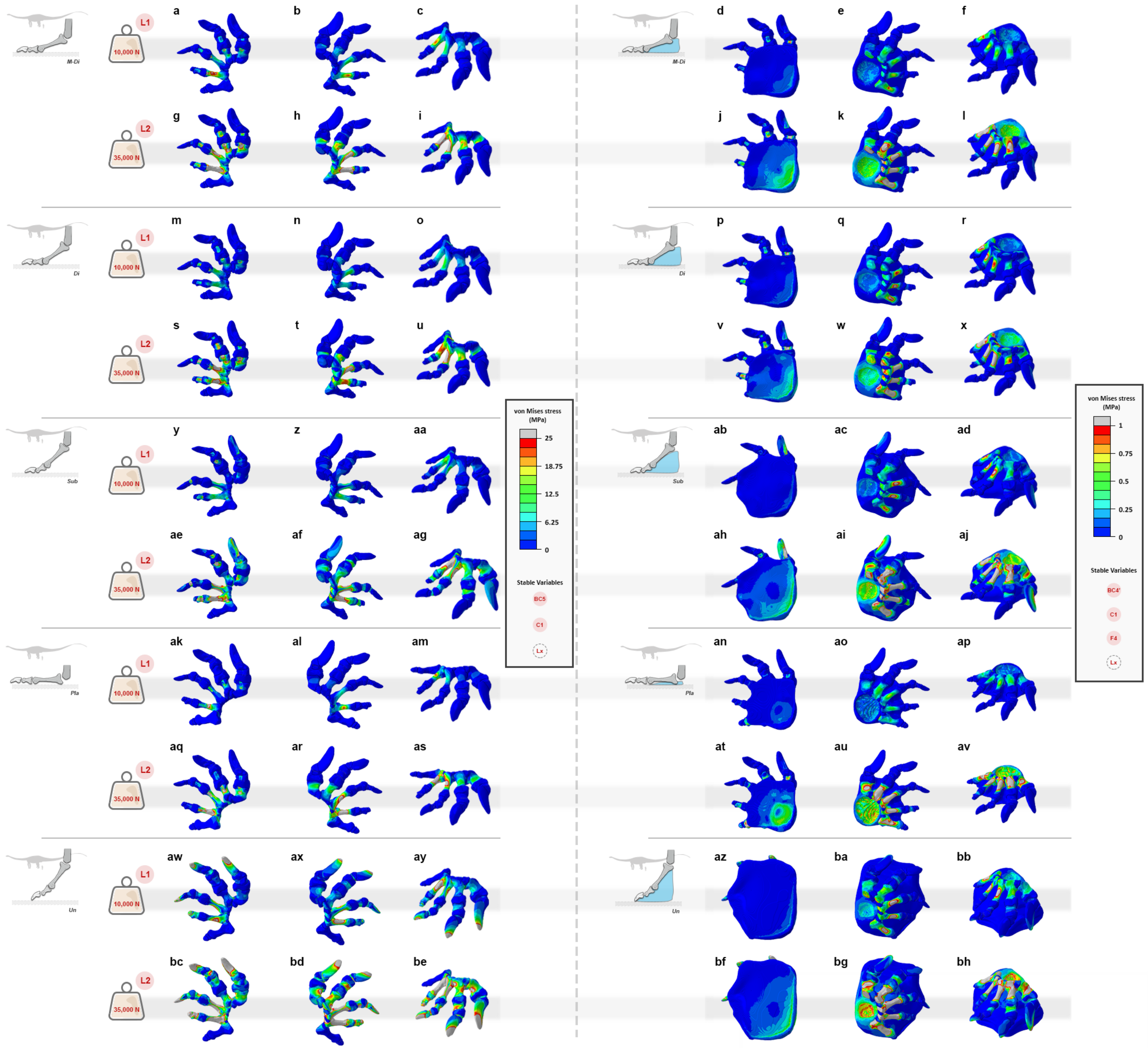

(legend on next page)

Fig. S31. Von Mises stress (MPa) distribution results for the sensitivity analysis between applied loads (proxy L1 vs physiologically realistic L2) in all skeletal morphotypes without (left) and with a hypothetical soft tissue pad (right) for *Diplodocus carnegii*. (a-l) Von Mises stress (MPa) distribution results for a mid-digitigrade morphotype under: (a-f) a proxy load L1, in: (a, d) plantar view; (b, e) dorsal view; and (c, f) craniomedial view. (g-l) a physiologically realistic load L2, in: (g, j) plantar view; (h, k) dorsal view; and (i, l) craniomedial view. (m-x) Von Mises stress (MPa) distribution results for a digitigrade morphotype under: (m-r) a proxy load L1, in: (m, p) plantar view; (n, q) dorsal view; and (o, r) craniomedial view. (s-x) a physiologically realistic load L2, in: (s, v) plantar view; (t, w) dorsal view; and (u, x) craniomedial view. (y-aj) Von Mises stress (MPa) distribution results for a subunguligrade morphotype under: (y-ad) a proxy load L1, in: (y, ab) plantar view; (z, ac) dorsal view; and (aa, ad) craniomedial view. (ae-aj) a physiologically realistic load L2, in: (ae, ah) plantar view; (af, ai) dorsal view; and (ag, aj) craniomedial view. (ak-av) Von Mises stress (MPa) distribution results for a plantigrade morphotype under: (ak-ap) a proxy load L1, in: (ak, an) plantar view; (al, ao) dorsal view; and (am, ap) craniomedial view. (aq-av) a physiologically realistic load L2, in: (aq, at) plantar view; (ar, au) dorsal view; and (as, av) craniomedial view. (aw-bh) Von Mises stress (MPa) distribution results for an unguligrade morphotype under: (aw-bb) a proxy load L1, in: (aw, az) plantar view; (ax, ba) dorsal view; and (ay, bb) craniomedial view. (bc-bh) a physiologically realistic load L2, in: (bc, bf) plantar view; (bd, bg) dorsal view; and (be, bh) craniomedial view. Cold (blue) and warm (red) colours show lower and higher von Mises stresses, respectively. Abbreviations: M-Di, Mid-Digitigrady; Di, Digitigrady; Sub, Subunguligrady; Pla, Plantigrady; Un, Unguligrady; BC5 & BC4', Boundary conditions 5 and 4', respectively; C1, Cartilage condition 1 (*E* value of 100 Mpa); F4, Soft tissue pad condition 4 (*E* value of 100 Mpa); L1, proxy load condition 1 (applied force of 10,000 N); L2, physiologically realistic load for *Diplodocus carnegii* (applied force of 35,000 N). Note: distinct scales of von Mises stresses used between left and right subdivisions.

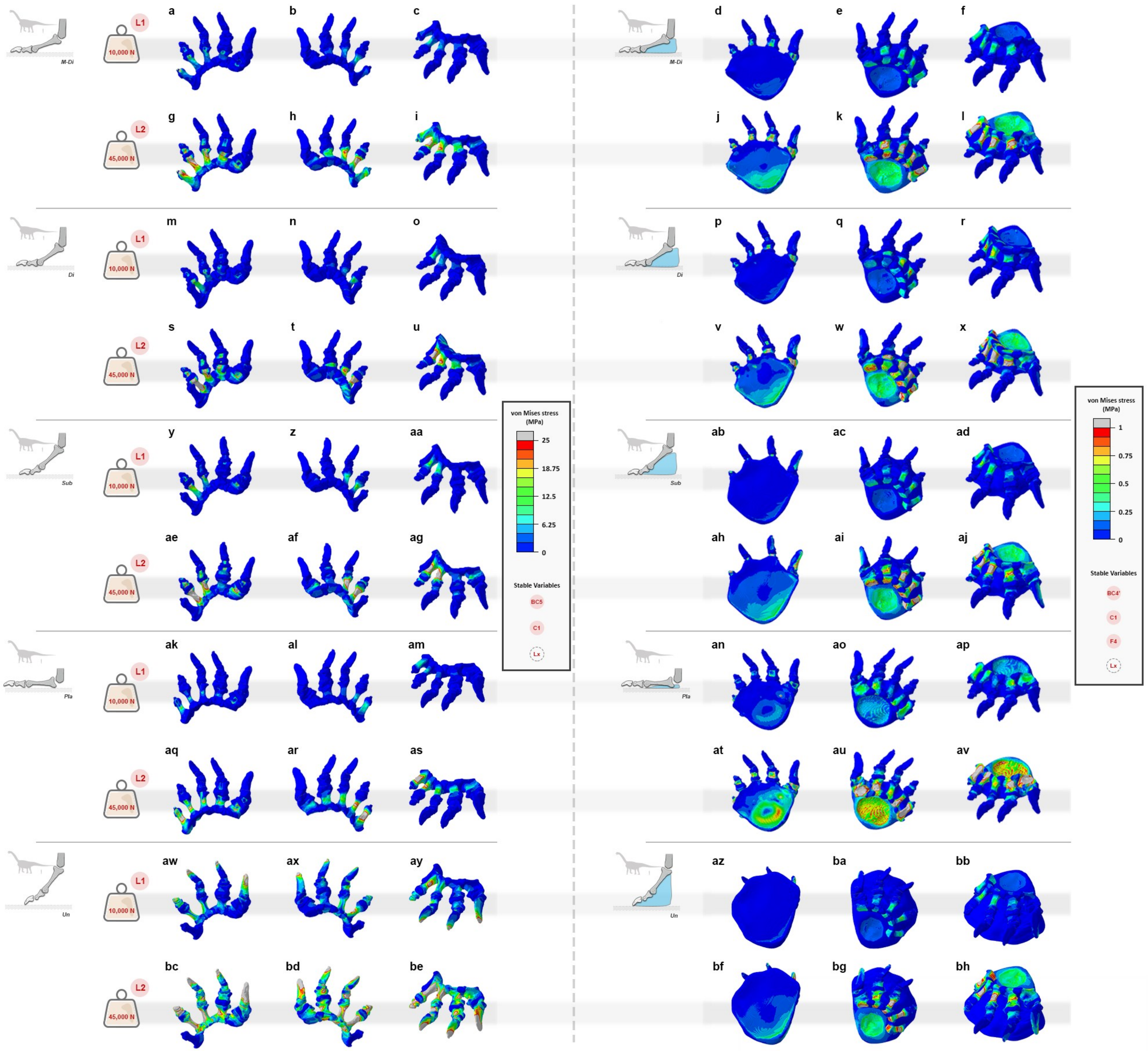

(legend on next page)

Fig. S32. Von Mises stress (MPa) distribution results for the sensitivity analysis between applied loads (proxy L1 vs physiologically realistic L2) in all skeletal morphotypes without (left) and with a hypothetical soft tissue pad (right) for *Camarasaurus* sp. (a-l) Von Mises stress (MPa) distribution results for a mid-digitigrade morphotype under: (a-f) a proxy load L1, in: (a, d) plantar view; (b, e) dorsal view; and (c, f) craniomedial view. (g-l) a physiologically realistic load L2, in: (g, j) plantar view; (h, k) dorsal view; and (i, l) craniomedial view. (m-x) Von Mises stress (MPa) distribution results for a digitigrade morphotype under: (m-r) a proxy load L1, in: (m, p) plantar view; (n, q) dorsal view; and (o, r) craniomedial view. (s-x) a physiologically realistic load L2, in: (s, v) plantar view; (t, w) dorsal view; and (u, x) craniomedial view. (y-aj) Von Mises stress (MPa) distribution results for a subunguligrade morphotype under: (y-ad) a proxy load L1, in: (y, ab) plantar view; (z, ac) dorsal view; and (aa, ad) craniomedial view. (ae-aj) a physiologically realistic load L2, in: (ae, ah) plantar view; (af, ai) dorsal view; and (ag, aj) craniomedial view. (ak-av) Von Mises stress (MPa) distribution results for a plantigrade morphotype under: (ak-ap) a proxy load L1, in: (ak, an) plantar view; (al, ao) dorsal view; and (am, ap) craniomedial view. (aq-av) a physiologically realistic load L2, in: (aq, at) plantar view; (ar, au) dorsal view; and (as, av) craniomedial view. (aw-bh) Von Mises stress (MPa) distribution results for an unguligrade morphotype under: (aw-bb) a proxy load L1, in: (aw, az) plantar view; (ax, ba) dorsal view; and (ay, bb) craniomedial view. (bc-bh) a physiologically realistic load L2, in: (bc, bf) plantar view; (bd, bg) dorsal view; and (be, bh) craniomedial view. Cold (blue) and warm (red) colours show lower and higher von Mises stresses, respectively. Abbreviations: M-Di, Mid-Digitigrady; Di, Digitigrady; Sub, Subunguligrady; Pla, Plantigrady; Un, Unguligrady; BC5 & BC4’, Boundary conditions 5 and 4’, respectively; C1, Cartilage condition 1 (*E* value of 100 Mpa); F4, Soft tissue pad condition 4 (*E* value of 100 Mpa); L1, proxy load condition 1 (applied force of 10,000 N); L2, physiologically realistic load for *Camarasaurus* sp. (applied force of 45,000 N). Note: distinct scales of von Mises stresses used between left and right subdivisions.

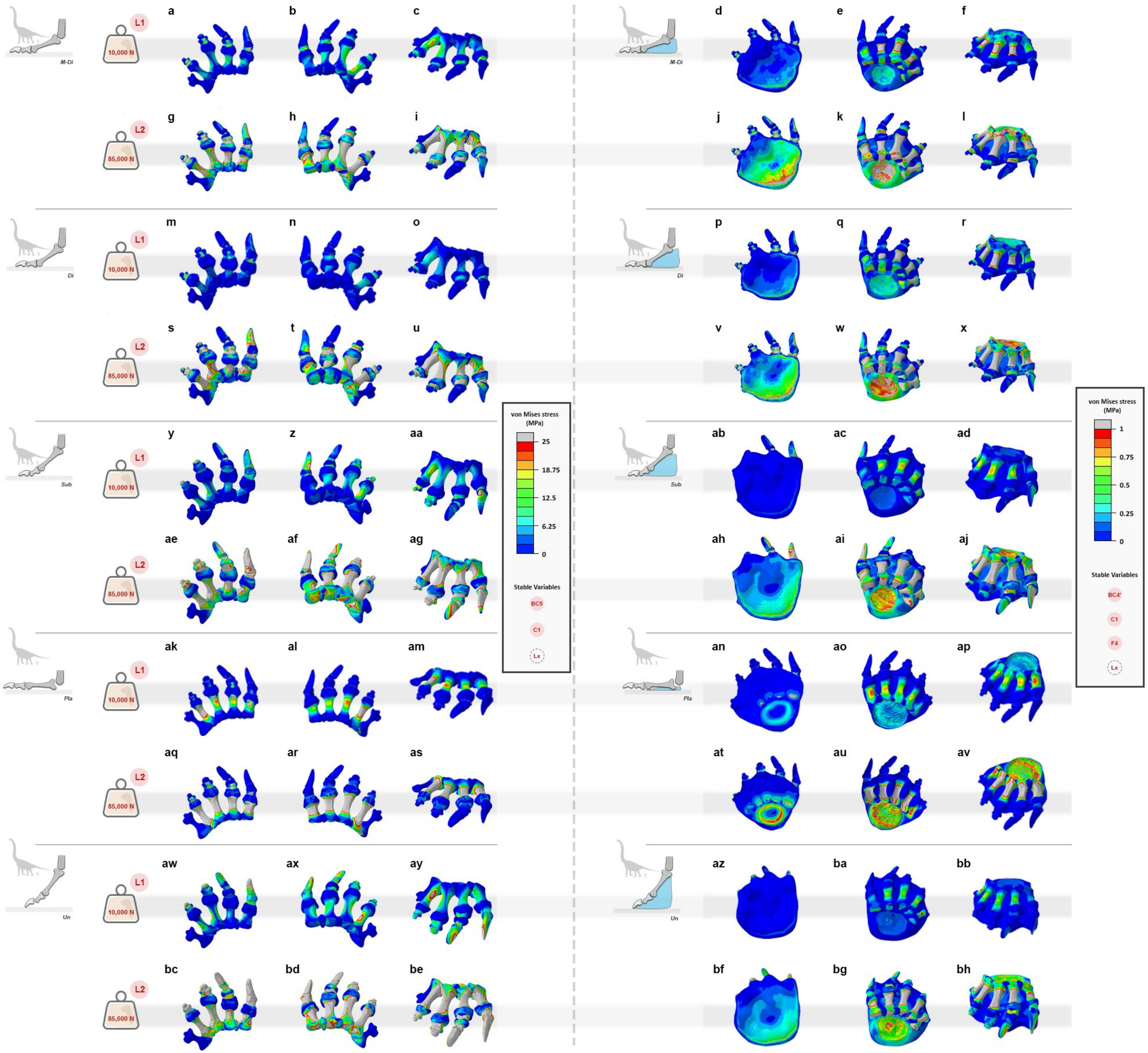

(legend on next page)

Fig. S33. Von Mises stress (MPa) distribution results for the sensitivity analysis between applied loads (proxy L1 vs physiologically realistic L2) in all skeletal morphotypes without (left) and with a hypothetical soft tissue pad (right) for *Giraffatitan brancai*. (a-l) Von Mises stress (MPa) distribution results for a mid-digitigrade morphotype under: (a-f) a proxy load L1, in: (a, d) plantar view; (b, e) dorsal view; and (c, f) craniomedial view. (g-l) a physiologically realistic load L2, in: (g, j) plantar view; (h, k) dorsal view; and (i, l) craniomedial view. (m-x) Von Mises stress (MPa) distribution results for a digitigrade morphotype under: (m-r) a proxy load L1, in: (m, p) plantar view; (n, q) dorsal view; and (o, r) craniomedial view. (s-x) a physiologically realistic load L2, in: (s, v) plantar view; (t, w) dorsal view; and (u, x) craniomedial view. (y-aj) Von Mises stress (MPa) distribution results for a subunguligrade morphotype under: (y-ad) a proxy load L1, in: (y, ab) plantar view; (z, ac) dorsal view; and (aa, ad) craniomedial view. (ae-aj) a physiologically realistic load L2, in: (ae, ah) plantar view; (af, ai) dorsal view; and (ag, aj) craniomedial view. (ak-av) Von Mises stress (MPa) distribution results for a plantigrade morphotype under: (ak-ap) a proxy load L1, in: (ak, an) plantar view; (al, ao) dorsal view; and (am, ap) craniomedial view. (aq-av) a physiologically realistic load L2, in: (aq, at) plantar view; (ar, au) dorsal view; and (as, av) craniomedial view. (aw-bh) Von Mises stress (MPa) distribution results for an unguligrade morphotype under: (aw-bb) a proxy load L1, in: (aw, az) plantar view; (ax, ba) dorsal view; and (ay, bb) craniomedial view. (bc-bh) a physiologically realistic load L2, in: (bc, bf) plantar view; (bd, bg) dorsal view; and (be, bh) craniomedial view. Cold (blue) and warm (red) colours show lower and higher von Mises stresses, respectively. Abbreviations: M-Di, Mid-Digitigrady; Di, Digitigrady; Sub, Subunguligrady; Pla, Plantigrady; Un, Unguligrady; BC5 & BC4', Boundary conditions 5 and 4', respectively; C1, Cartilage condition 1 (*E* value of 100 Mpa); F4, Soft tissue pad condition 4 (*E* value of 100 Mpa); L1, proxy load condition 1 (applied force of 10,000 N); L2, physiologically realistic load for *Giraffatitan brancai* (applied force of 85,000 N). Note: distinct scales of von Mises stresses used between left and right subdivisions.

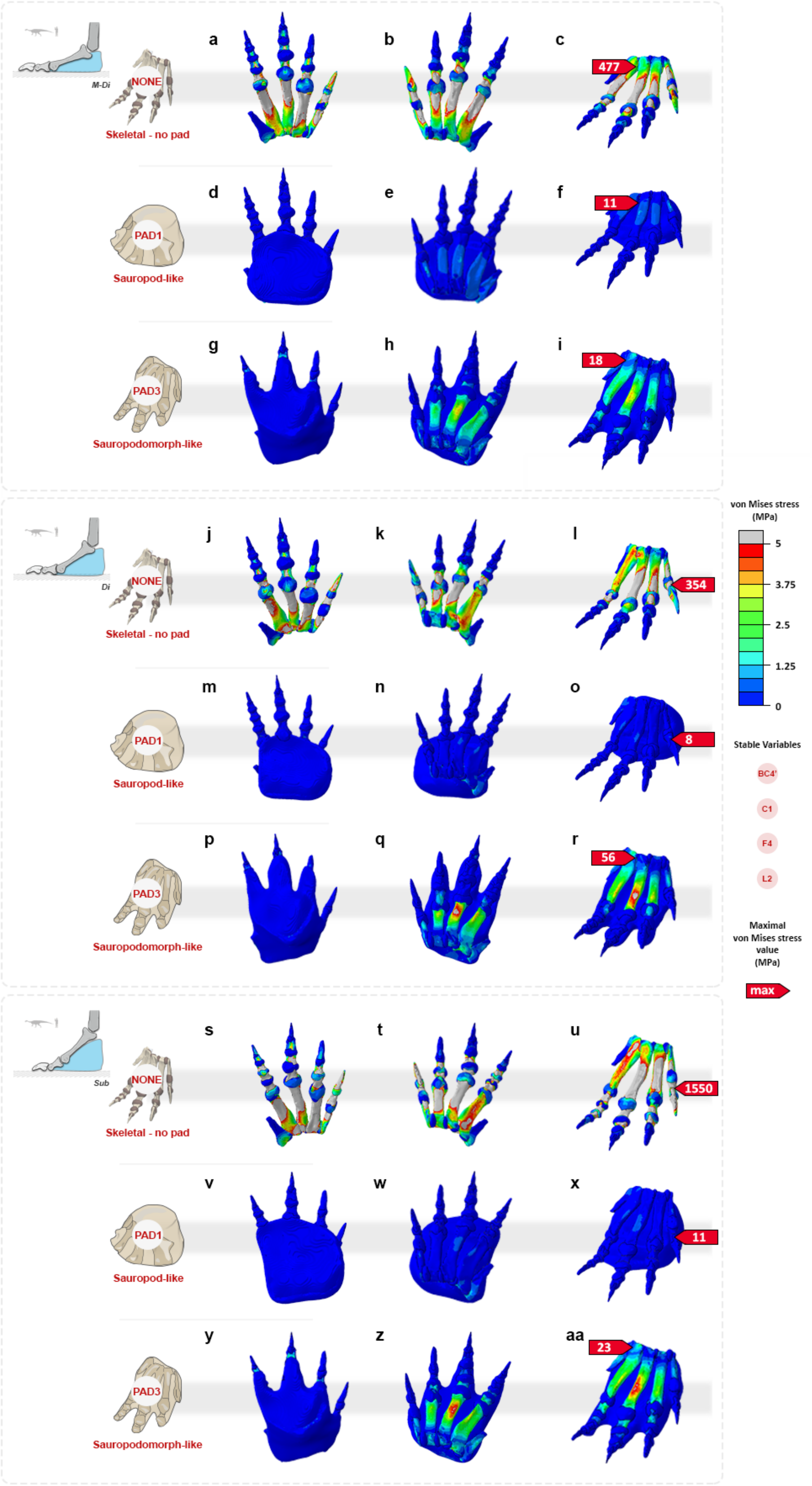

(legend on next page)

Fig. S34. Von Mises stress (MPa) distribution results for the sensitivity analysis of the incipient soft tissue pad outlines in the mid-digitigrade, digitigrade, and subunguligrade postural morphotypes of *Plateosaurus engelhardti*. (a-i) FEM of a mid-digitigrade morphotype, with: (a-c) Von Mises stress (MPa) distribution results for a skeletal pes without soft tissue pad, in: (a) plantar view; (b) dorsal view; and (c) craniomedial view; (d-f) Von Mises stress (MPa) distribution results for a soft tissue pad extended from the tarsus-crus complex to the plantar surfaces of the first joints in contact with the substrate (PAD1), in: (d) plantar view; (e) dorsal view; and (f) craniomedial view. (g-i) Von Mises stress (MPa) distribution results for an incipient soft tissue pad constrained caudally below the tarsus and crus complex, and extended below the plantar surfaces of each digit in contact with the substrate but not connected between the digits (PAD3); in: (g) plantar view; (h) dorsal view; and (i) craniomedial view. (j-r) FEM of a digitigrade morphotype, with: (j-l) Von Mises stress (MPa) distribution results for a skeletal pes without soft tissue pad, in: (j) plantar view; (k) dorsal view; and (l) craniomedial view; (m-o) Von Mises stress (MPa) distribution results for a soft tissue pad extended from the tarsus-crus complex to the plantar surfaces of the first joints in contact with the substrate (PAD1), in: (m) plantar view; (n) dorsal view; and (o) craniomedial view. (p-r) Von Mises stress (MPa) distribution results for an incipient soft tissue pad constrained caudally below the tarsus and crus complex, and extended below the plantar surfaces of each digit in contact with the substrate but not connected between the digits (PAD3); in: (p) plantar view; (q) dorsal view; and (r) craniomedial view. (s-aa) FEM of a mid-digitigrade morphotype, with: (s-u) Von Mises stress (MPa) distribution results for a skeletal pes without soft tissue pad, in: (s) plantar view; (t) dorsal view; and (u) craniomedial view; (v-x) Von Mises stress (MPa) distribution results for a soft tissue pad extended from the tarsus-crus complex to the plantar surfaces of the first joints in contact with the substrate (PAD1), in: (v) plantar view; (w) dorsal view; and (x) craniomedial view. (y-aa) Von Mises stress (MPa) distribution results for an incipient soft tissue pad constrained caudally below the tarsus and crus complex, and extended below the plantar surfaces of each digit in contact with the substrate but not connected between the digits (PAD3); in: (y) plantar view; (z) dorsal view; and (aa) craniomedial view. Cold (blue) and warm (red) colours show lower, and higher von Mises stresses, respectively. In each case, the maximum von Mises stress value is indicated in a red rectangle pointing at its location in each respective FEM. Abbreviations: M-Di, Mid-Digitigrady; Di, Digitigrady; Sub, Subunguligrady; BC4', Boundary condition 4'; C1, Cartilage condition 1 (*E* value of 100 Mpa); F4, Soft tissue pad condition 4 (*E* value of 100 Mpa); L2, Loading condition 2 (applied force of 2,000 N); PAD1, Sauropod-like soft tissue pad outline; PAD3, incipient soft tissue pad outline.

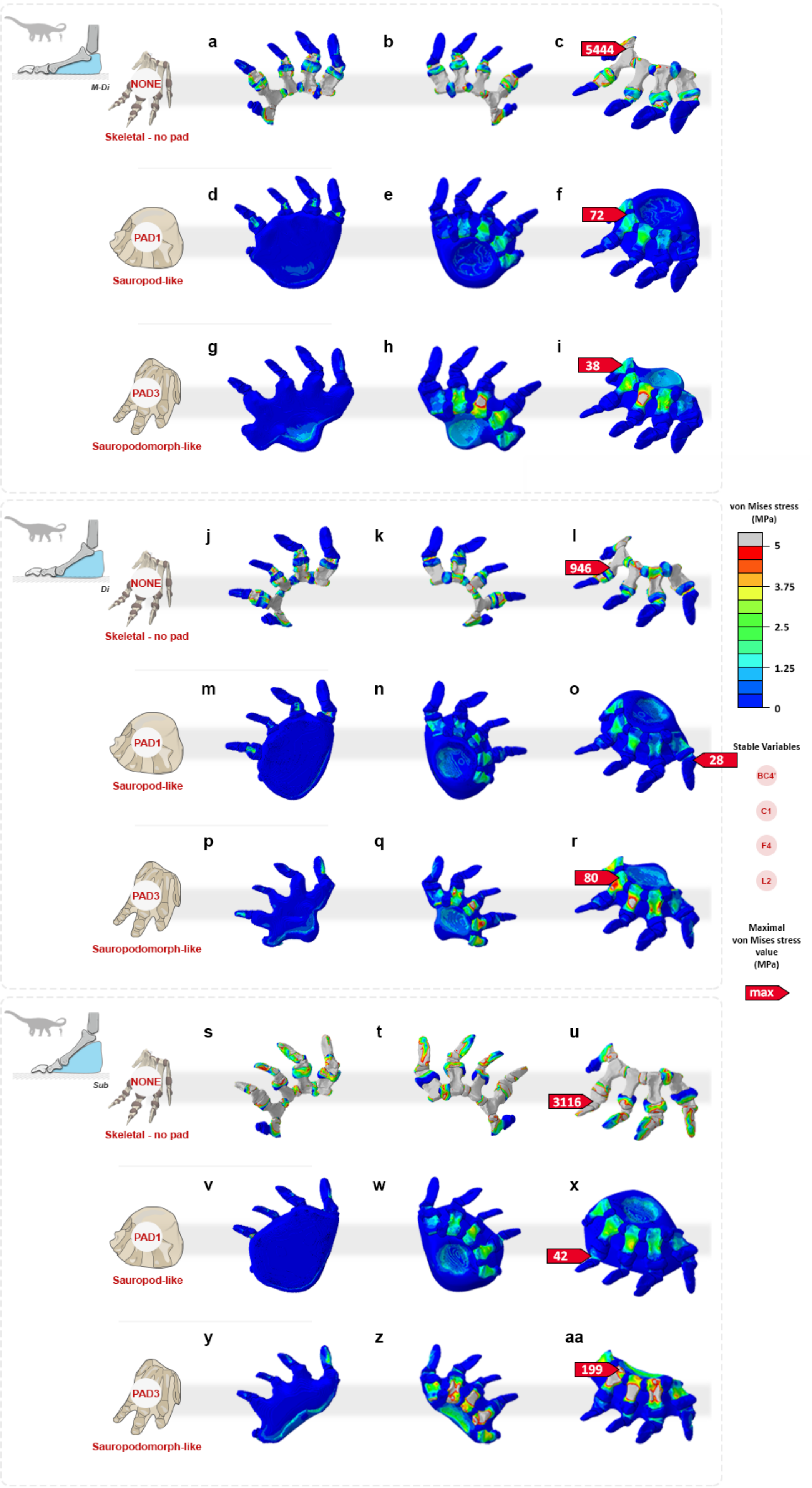

(legend on next page)

Fig. S35. Von Mises stress (MPa) distribution results for the sensitivity analysis of the incipient soft tissue pad outlines in the mid-digitigrade, digitigrade, and subunguligrade postural morphotypes of *Rhoetosaurus brownei*. (a-i) FEM of a mid-digitigrade morphotype, with: (a-c) Von Mises stress (MPa) distribution results for a skeletal pes without soft tissue pad, in: (a) plantar view; (b) dorsal view; and (c) craniomedial view; (d-f) Von Mises stress (MPa) distribution results for a soft tissue pad extended from the tarsus-crus complex to the plantar surfaces of the first joints in contact with the substrate (PAD1), in: (d) plantar view; (e) dorsal view; and (f) craniomedial view. (g-i) Von Mises stress (MPa) distribution results for an incipient soft tissue pad constrained caudally below the tarsus and crus complex, and extended below the plantar surfaces of each digit in contact with the substrate but not connected between the digits (PAD3); in: (g) plantar view; (h) dorsal view; and (i) craniomedial view. (j-r) FEM of a digitigrade morphotype, with: (j-l) Von Mises stress (MPa) distribution results for a skeletal pes without soft tissue pad, in: (j) plantar view; (k) dorsal view; and (l) craniomedial view; (m-o) Von Mises stress (MPa) distribution results for a soft tissue pad extended from the tarsus-crus complex to the plantar surfaces of the first joints in contact with the substrate (PAD1), in: (m) plantar view; (n) dorsal view; and (o) craniomedial view. (p-r) Von Mises stress (MPa) distribution results for an incipient soft tissue pad constrained caudally below the tarsus and crus complex, and extended below the plantar surfaces of each digit in contact with the substrate but not connected between the digits (PAD3); in: (p) plantar view; (q) dorsal view; and (r) craniomedial view. (s-aa) FEM of a mid-digitigrade morphotype, with: (s-u) Von Mises stress (MPa) distribution results for a skeletal pes without soft tissue pad, in: (s) plantar view; (t) dorsal view; and (u) craniomedial view; (v-x) Von Mises stress (MPa) distribution results for a soft tissue pad extended from the tarsus-crus complex to the plantar surfaces of the first joints in contact with the substrate (PAD1), in: (v) plantar view; (w) dorsal view; and (x) craniomedial view. (y-aa) Von Mises stress (MPa) distribution results for an incipient soft tissue pad constrained caudally below the tarsus and crus complex, and extended below the plantar surfaces of each digit in contact with the substrate but not connected between the digits (PAD3); in: (y) plantar view; (z) dorsal view; and (aa) craniomedial view. Cold (blue) and warm (red) colours show lower, and higher von Mises stresses, respectively. In each case, the maximum von Mises stress value is indicated in a red rectangle pointing at its location in each respective FEM. Abbreviations: M-Di, Mid-Digitigrady; Di, Digitigrady; Sub, Subunguligrady; BC4', Boundary condition 4'; C1, Cartilage condition 1 (*E* value of 100 Mpa); F4, Soft tissue pad condition 4 (*E* value of 100 Mpa); L2, Loading condition 2 (applied force of 2,000 N); PAD1, Sauropod-like soft tissue pad outline; PAD3, an incipient soft tissue pad outline.

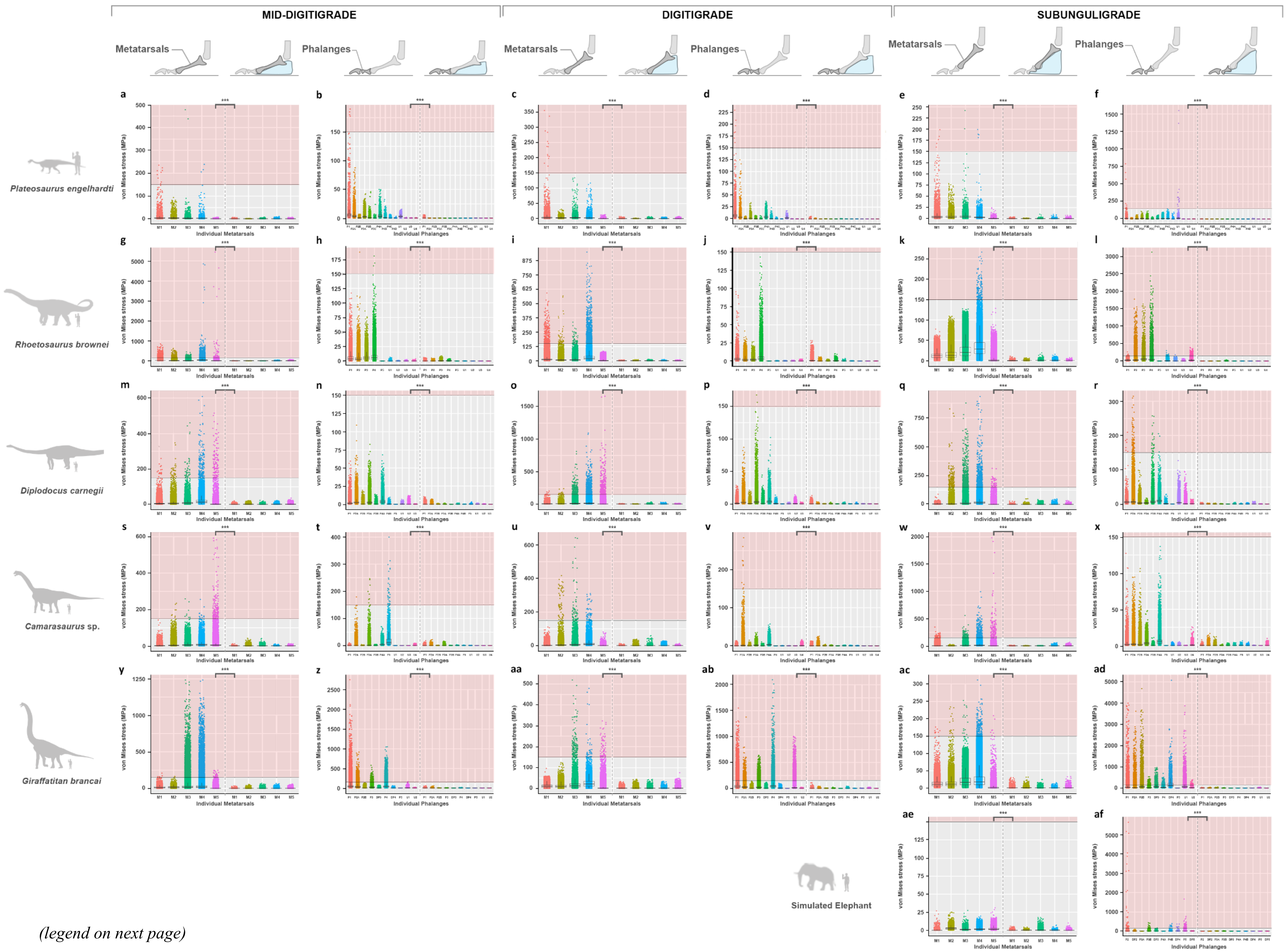

(legend on next page)

Fig. S36. Comparisons in von Mises stress values (MPa) recorded on the nodes of each metatarsal and each phalanx for all pedal postural morphotypes between the skeletal FEMs without and with the presence of a hypothetical soft tissue pad for all studied specimens. (a-f) Von Mises stress values (MPa) of all the nodes in the FEMs of *Plateosaurus engelhardti,* within the: (a-b) mid-digitigrade posture, for: (a) each metatarsal, and (b) each phalanx. (c-d) digitigrade posture, for: (c) each metatarsal, and (d) each phalanx. (e-f) subunguligrade posture, for: (e) each metatarsal, and (f) each phalanx. (g-l) Von Mises stress values (MPa) of all the nodes in the FEMs of *Rhoetosaurus brownei,* within the: (g-h) mid-digitigrade posture, for: (g) each metatarsal, and (h) each phalanx. (i-j) digitigrade posture, for: (i) each metatarsal, and (j) each phalanx. (k-l) subunguligrade posture, for: (k) each metatarsal, and (l) each phalanx. (m-r) Von Mises stress values (MPa) of all the nodes in the FEMs of *Diplodocus carnegii,* within the: (m-n) mid-digitigrade posture, for: (m) each metatarsal, and (n) each phalanx. (o-p) digitigrade posture, for: (o) each metatarsal, and (p) each phalanx. (q-r) subunguligrade posture, for: (q) each metatarsal, and (r) each phalanx. (s-x) Von Mises stress values (MPa) of all the nodes in the FEMs of *Camarasaurus* sp.*,* within the: (s-t) mid-digitigrade posture, for: (s) each metatarsal, and (t) each phalanx. (u-v) digitigrade posture, for: (u) each metatarsal, and (v) each phalanx. (w-x) subunguligrade posture, for: (w) each metatarsal, and (x) each phalanx. (y-ad) Von Mises stress values (MPa) of all the nodes in the FEMs of *Giraffatitan brancai,* within the: (y-z) mid-digitigrade posture, for: (y) each metatarsal, and (z) each phalanx. (aa-ab) digitigrade posture, for: (aa) each metatarsal, and (ab) each phalanx. (ac-ad) subunguligrade posture, for: (ac) each metatarsal, and (ad) each phalanx. (ae-af) Von Mises stress values (MPa) of all the nodes in the FEMs of the simulated elephant pes in the subunguligrade posture*,* for: (ae) each metatarsal, and (af) each phalanx. Individual data points (dot) represent von Mises stress value for each individual node for each component (i.e., metatarsal and phalanx). Red backgrounds denote the theoretical limit of 150 MPa, representing the safety margin of a bone (following the value reported in the literature of what the bone of a human and bovid femur is capable of withstanding before physical damage; *44*, *45*). ***, p<0.001. Detailed statistical results provided in Table S8. Note: We purposely varied the von Mises stress scale to illustrate better the individual details of each graphic.

(*44, 45*)

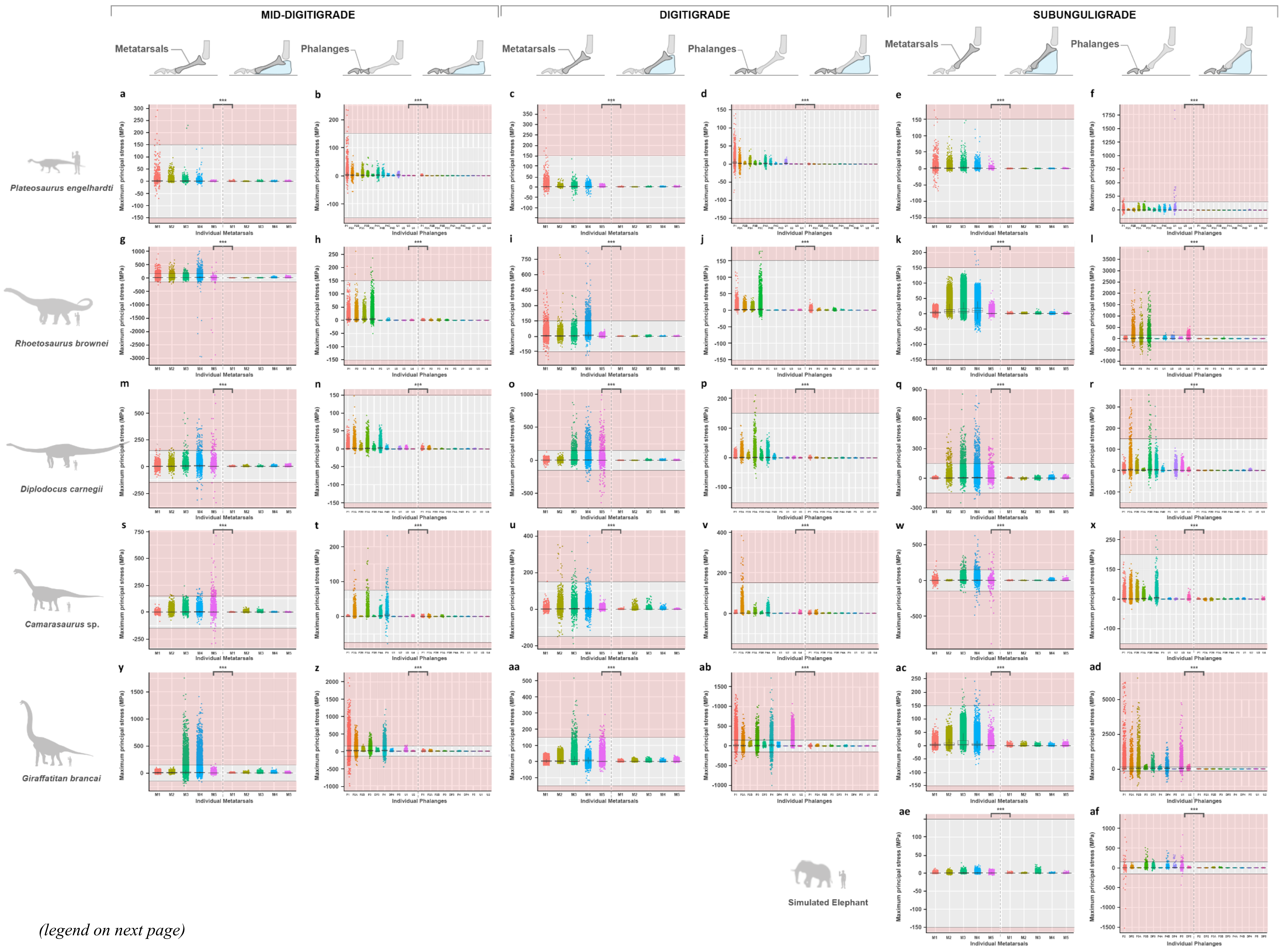

(legend on next page)

Fig. S37. Comparisons in Maximum Principal stress values (MPa) recorded on the nodes of each metatarsal and each phalanx for all pedal postural morphotypes between the skeletal FEMs without and with the presence of a hypothetical soft tissue pad for all studied specimens. (a-f) Maximum Principal stress values (MPa) of all the nodes in the FEMs of *Plateosaurus engelhardti,* within the: (a-b) mid-digitigrade posture, for: (a) each metatarsal, and (b) each phalanx. (c-d) digitigrade posture, for: (c) each metatarsal, and (d) each phalanx. (e-f) subunguligrade posture, for: (e) each metatarsal, and (f) each phalanx. (g-l) Maximum Principal stress values (MPa) of all the nodes in the FEMs of *Rhoetosaurus brownei,* within the: (g-h) mid-digitigrade posture, for: (g) each metatarsal, and (h) each phalanx. (i-j) digitigrade posture, for: (i) each metatarsal, and (j) each phalanx. (k-l) subunguligrade posture, for: (k) each metatarsal, and (l) each phalanx. (m-r) Maximum Principal stress values (MPa) of all the nodes in the FEMs of *Diplodocus carnegii,* within the: (m-n) mid-digitigrade posture, for: (m) each metatarsal, and (n) each phalanx. (o-p) digitigrade posture, for: (o) each metatarsal, and (p) each phalanx. (q-r) subunguligrade posture, for: (q) each metatarsal, and (r) each phalanx. (s-x) Maximum Principal stress values (MPa) of all the nodes in the FEMs of *Camarasaurus* sp.*,* within the: (s-t) mid-digitigrade posture, for: (s) each metatarsal, and (t) each phalanx. (u-v) digitigrade posture, for: (u) each metatarsal, and (v) each phalanx. (w-x) subunguligrade posture, for: (w) each metatarsal, and (x) each phalanx. (y-ad) Maximum Principal stress values (MPa) of all the nodes in the FEMs of *Giraffatitan brancai,* within the: (y-z) mid-digitigrade posture, for: (y) each metatarsal, and (z) each phalanx. (aa-ab) digitigrade posture, for: (aa) each metatarsal, and (ab) each phalanx. (ac-ad) subunguligrade posture, for: (ac) each metatarsal, and (ad) each phalanx. (ae-af) Maximum Principal stress values (MPa) of all the nodes in the FEMs of the simulated elephant pes in the subunguligrade posture*,* for: (ae) each metatarsal, and (af) each phalanx. Individual data points (dot) represent Maximum Principal stress value for each individual node for each component (i.e., metatarsal and phalanx). Red backgrounds denote the theoretical limit of 150 MPa, representing the safety margin of a bone (following the value reported in the literature of what the bone of a human and bovid femur is capable of withstanding before physical damage; *44*, *45*). ***, p<0.001. Detailed statistical results provided in Table S8. Note: We purposely varied the Maximum Principal stress scale to illustrate better the individual details of each graphic

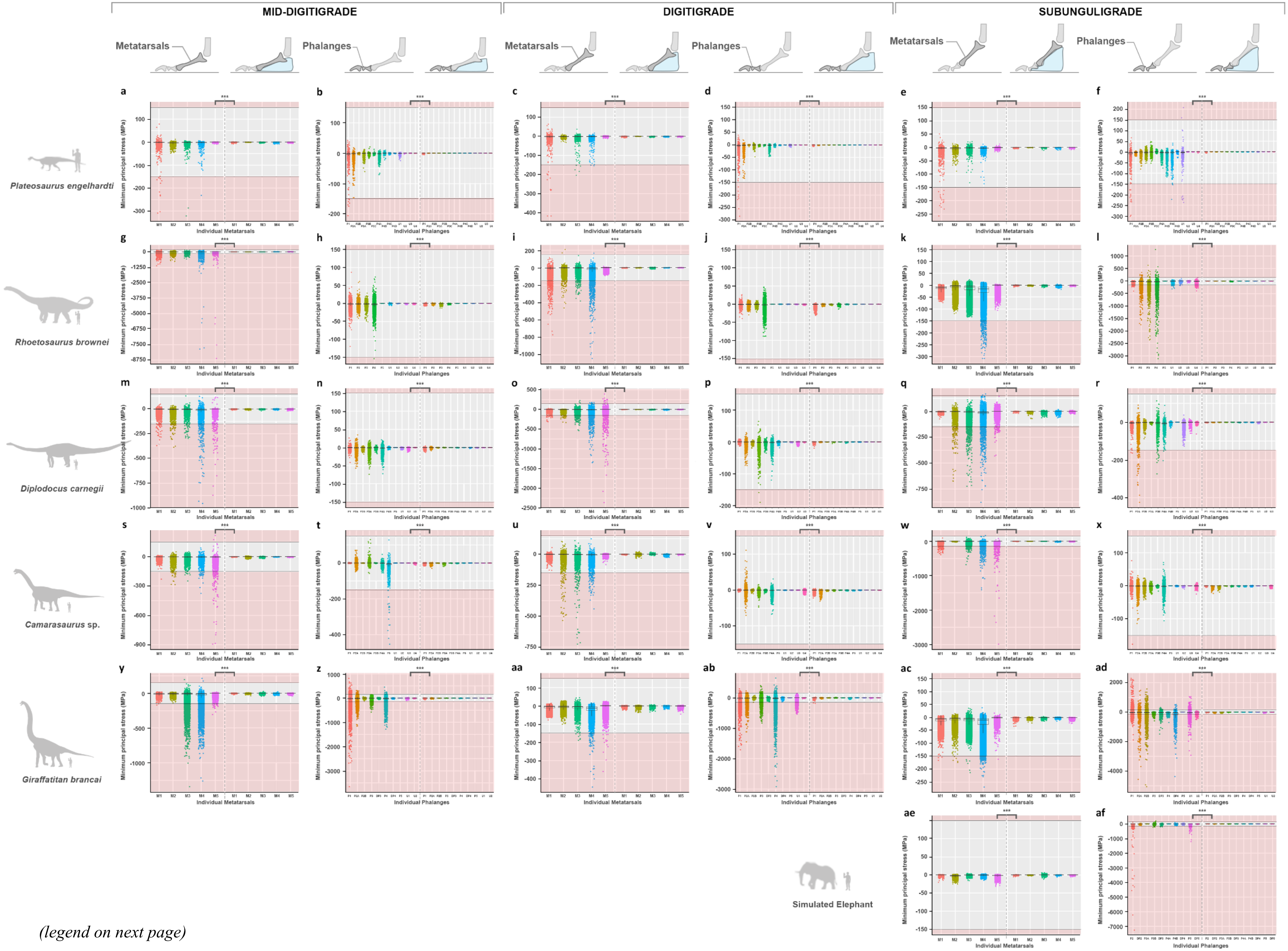

(legend on next page)

Fig. S38. Comparisons in Minimum Principal stress values (MPa) recorded on the nodes of each metatarsal and each phalanx for all pedal postural morphotypes between the skeletal FEMs without and with the presence of a hypothetical soft tissue pad for all studied specimens. (a-f) Minimum Principal stress values (MPa) of all the nodes in the FEMs of *Plateosaurus engelhardti,* within the: (a-b) mid-digitigrade posture, for: (a) each metatarsal, and (b) each phalanx. (c-d) digitigrade posture, for: (c) each metatarsal, and (d) each phalanx. (e-f) subunguligrade posture, for: (e) each metatarsal, and (f) each phalanx. (g-l) Minimum Principal stress values (MPa) of all the nodes in the FEMs of *Rhoetosaurus brownei,* within the: (g-h) mid-digitigrade posture, for: (g) each metatarsal, and (h) each phalanx. (i-j) digitigrade posture, for: (i) each metatarsal, and (j) each phalanx. (k-l) subunguligrade posture, for: (k) each metatarsal, and (l) each phalanx. (m-r) Minimum Principal stress values (MPa) of all the nodes in the FEMs of *Diplodocus carnegii,* within the: (m-n) mid-digitigrade posture, for: (m) each metatarsal, and (n) each phalanx. (o-p) digitigrade posture, for: (o) each metatarsal, and (p) each phalanx. (q-r) subunguligrade posture, for: (q) each metatarsal, and (r) each phalanx. (s-x) Minimum Principal stress values (MPa) of all the nodes in the FEMs of *Camarasaurus* sp.*,* within the: (s-t) mid-digitigrade posture, for: (s) each metatarsal, and (t) each phalanx. (u-v) digitigrade posture, for: (u) each metatarsal, and (v) each phalanx. (w-x) subunguligrade posture, for: (w) each metatarsal, and (x) each phalanx. (y-ad) Minimum Principal stress values (MPa) of all the nodes in the FEMs of *Giraffatitan brancai,* within the: (y-z) mid-digitigrade posture, for: (y) each metatarsal, and (z) each phalanx. (aa-ab) digitigrade posture, for: (aa) each metatarsal, and (ab) each phalanx. (ac-ad) subunguligrade posture, for: (ac) each metatarsal, and (ad) each phalanx. (ae-af) Minimum Principal stress values (MPa) of all the nodes in the FEMs of the simulated elephant pes in the subunguligrade posture*,* for: (ae) each metatarsal, and (af) each phalanx. Individual data points (dot) represent Minimum Principal stress value for each individual node for each component (i.e., metatarsal and phalanx). Red backgrounds denote the theoretical limit of 150 MPa, representing the safety margin of a bone (following the value reported in the literature of what the bone of a human and bovid femur is capable of withstanding before physical damage; *44*, *45*). ***, p<0.001. Detailed statistical results provided in Table S8. Note: We purposely varied the Minimum Principal stress scale to illustrate better the individual details of each graphic

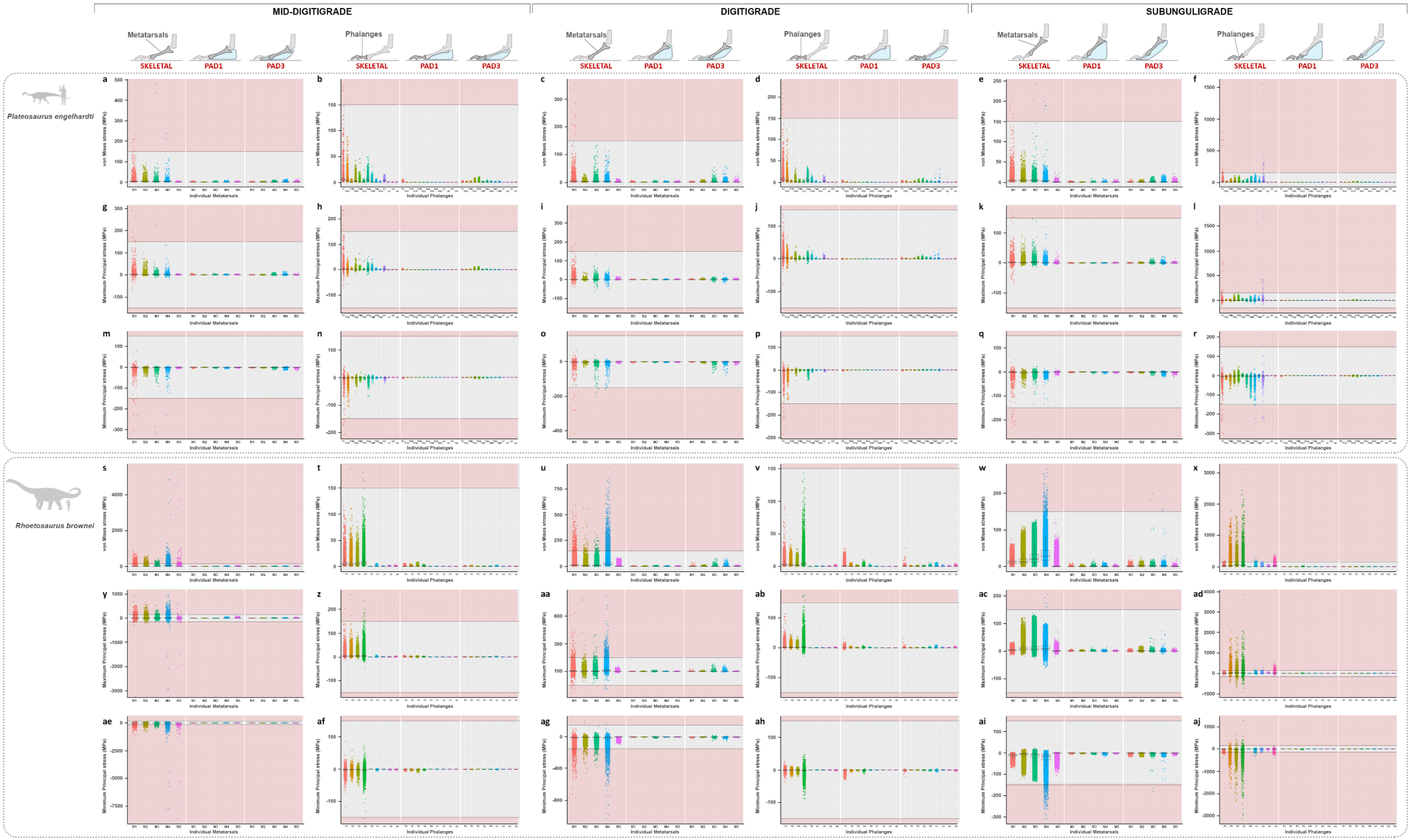

(legend on next page)

Fig. S39. Comparisons in von Mises stress, Maximum Principal and Minimum Principal stress values (MPa) recorded in each metatarsal and each phalanx for all pedal postural morphotypes between the skeletal FEMs without and with the presence of a soft tissue pad (PAD1) and an incipient soft tissue pad (PAD3) for *Plateosaurus engelhardti* and *Rhoetosaurus brownei*. (a-f) von Mises stress values (MPa) of all the elements in the FEMs of *Plateosaurus engelhardti,* within the: (a-b) mid-digitigrade posture, for: (a) each metatarsal, and (b) each phalanx. (c-d) digitigrade posture, for: (c) each metatarsal, and (d) each phalanx. (e-f) subunguligrade posture, for: (e) each metatarsal, and (f) each phalanx. (g-l) Maximum Principal stress values (MPa) of all the elements in the FEMs of *Plateosaurus engelhardti,* within the: (g-h) mid-digitigrade posture, for: (g) each metatarsal, and (h) each phalanx. (i-j) digitigrade posture, for: (i) each metatarsal, and (j) each phalanx. (k-l) subunguligrade posture, for: (k) each metatarsal, and (l) each phalanx. (m-r) Minimum Principal stress values (MPa) of all the elements in the FEMs of *Plateosaurus engelhardti,* within the: (m-n) mid-digitigrade posture, for: (m) each metatarsal, and (n) each phalanx. (o-p) digitigrade posture, for: (o) each metatarsal, and (p) each phalanx. (q-r) subunguligrade posture, for: (q) each metatarsal, and (r) each phalanx. (s-x) von Mises stress values (MPa) of all the elements in the FEMs of *Rhoetosaurus brownei,* within the: (s-t) mid-digitigrade posture, for: (s) each metatarsal, and (t) each phalanx. (u-v) digitigrade posture, for: (u) each metatarsal, and (v) each phalanx. (w-x) subunguligrade posture, for: (w) each metatarsal, and (x) each phalanx. (y-ad) Maximum Principal stress values (MPa) of all the elements in the FEMs of *Rhoetosaurus brownei,* within the: (y-z) mid-digitigrade posture, for: (y) each metatarsal, and (z) each phalanx. (aa-ab) digitigrade posture, for: (aa) each metatarsal, and (ab) each phalanx. (ac-ad) subunguligrade posture, for: (ac) each metatarsal, and (ad) each phalanx. (ae-af) Minimum Principal stress values (MPa) of all the elements in the FEMs of *Rhoetosaurus brownei,* within the: (ae-af) mid-digitigrade posture, for: (ae) each metatarsal, and (af) each phalanx. (ag-ah) digitigrade posture, for: (ag) each metatarsal, and (ah) each phalanx. (ai-aj) subunguligrade posture, for: (ai) each metatarsal, and (aj) each phalanx. Individual data points (dot) represent von Mises stress, Maximum Principal and Minimum Principal stress value for each individual element for each component (i.e., metatarsal and phalanx), respectively. Red backgrounds denote the theoretical limit of 150 MPa, representing the safety margin of a bone (following the value reported in the literature of what the bone of a human and bovid femur is capable of withstanding before physical damage; *44*, *45*). Note: We purposely varied the stress scale to illustrate better the individual details of each graphic.

Table S1. Proportions of cartilage gaps for each digital segment in the pes of *Plateosaurus engelhardti* (POL70), following measurements taken by AJ and the proportions measured from (*92*). *Abbreviations*: *L*, Length; *SL*, Segment Length.

Digits	*Bones*	*L (mm)*	*SL (mm)*	*Alligator (10.8%)*	*Struthio (6.8%)*	*Coturnix (1.8%)*
**DI**	MtI	135	198	21.38	13.46	3.56
	PhI-1	63	148	15.98	10.06	2.66
	PhI-2	85				
	Total		***346***	***37.37***	***23.528***	***6.228***
**DII**	MtII	229	301	32.51	20.47	5.42
	PhII-1	72	131	14.15	8.91	2.36
	PhII-2	59	136	14.69	9.25	2.45
	PhII-3	77				
	Total		***568***	***61.34***	***38.62***	***10.22***
**DIII**	MtIII	272	354	38.23	24.07	6.37
	PhIII-1	82	150	16.20	10.20	2.70
	PhIII-2	68	116	12.53	7.89	2.09
	PhIII-3	48	125	13.50	8.50	2.25
	PhIII-4	77				
	Total		***745***	***80.46***	***50.66***	***13.41***
**DIV**	MtIV	236	303	32.72	20.60	5.45
	PhIV-1	67	130	14.04	8.84	2.34
	PhIV-2	63	108	11.66	7.34	1.94
	PhIV-3	45	88	9.50	5.98	1.58
	PhIV-4	43	95	10.26	6.46	1.71
	PhIV-5	52				
	Total		***724***	***78.19***	***49.23***	***13.03***
**DV**	MtV	126				
			**AVERAGE**	**18.38**	**11.57**	**3.06**
			**SD**	**9.27**	**5.84**	**1.55**

Table S2. Proportions of cartilage gaps for each digital segment in the pes of *Rhoetosaurus brownei* (QM F1659), following measurements from (*17*) and taken by AJ, and the proportions measured from (*92*). *Abbreviations*: *L*, Length; *SL*, Segment Length.

Digits	*Bones*	*L (mm)*	*SL (mm)*	*Alligator (10.8%)*	*Struthio (6.8%)*	*Coturnix (1.8%)*
**DI**	MtI	230	318	34.34	21.62	5.72
	PhI-1	88	338	36.50	22.98	6.08
	PhI-2	250				
	Total		***568***	***61.34***	***38.62***	***10.22***
**DII**	MtII	270	356	38.45	24.21	6.41
	PhII-1	86	142	15.34	9.66	2.56
	PhII-2	56	261	28.19	17.75	4.70
	PhII-3	205				
	Total		***617***	***66.64***	***41.96***	***11.11***
**DIII**	MtIII	289	371	34.34	21.62	5.72
	PhIII-1	82	114	36.50	22.98	6.08
	PhIII-2	32	74	38.66	24.34	6.44
	PhIII-3	42	201	40.82	25.70	6.80
	PhIII-4	159				
	Total		***604***	***65.23***	***41.07***	***10.87***
**DIV**	MtIV	269	368	34.34	21.62	5.72
	PhIV-1	99	149	36.50	22.98	6.08
	PhIV-2	50	194	38.66	24.34	6.44
	PhIV-3	144				
	Total		***562***	***60.70***	***38.22***	***10.12***
**DV**	MtV	NA	NA	NA	NA	NA
	PhV-1	NA	NA	NA	NA	NA
			**AVERAGE**	**34.39**	**21.65**	**5.73**
			**SD**	**6.80**	**4.28**	**1.13**

Table S3. Proportions of cartilage gaps for each digital segment in the pes of *Diplodocus carnegii* (CM[NH] 94), following measurements taken by AJ and the proportions measured from (*92*). *Abbreviations*: *L*, Length; *SL*, Segment Length

Digits	*Bones*	*L (mm)*	*SL (mm)*	*Alligator (10.8%)*	*Struthio (6.8%)*	*Coturnix (1.8%)*
**DI**	MtI	155	215	23.22	14.62	3.87
	PhI-1	60	270	29.16	18.36	4.86
	PhI-2	210				
	Total		***485***	***52.38***	***32.98***	***8.73***
**DII**	MtII	145	220	23.76	14.96	3.96
	PhII-1	75	100	10.80	6.80	1.80
	PhII-2	25	175	18.90	11.90	3.15
	PhII-3	150				
	Total		***495***	***53.46***	***33.66***	***8.91***
**DIII**	MtIII	195	260	28.08	17.68	4.68
	PhIII-1	65	90	9.72	6.12	1.62
	PhIII-2	25	145	15.66	9.86	2.61
	PhIII-3	120				
	Total		***495***	***53.46***	***33.66***	***8.91***
**DIV**	MtIV	195	265	28.62	18.02	4.77
	PhIV-1	70	90	9.72	6.12	1.62
	PhIV-2	20				
	Total		***355***	***38.34***	***24.14***	***6.39***
**DV**	MtV	200	240	25.92	16.32	4.32
	PhV-1	40				
	Total			***25.92***	***16.32***	***4.32***
			**AVERAGE**	**20.32**	**12.80**	**3.39**
			**SD**	**7.73**	**4.87**	**1.29**

Table S4. Proportions of cartilage gaps for each digital segment in the pes of *Camarasaurus sp.* (?YPM 1901), following measurements taken by AJ and the proportions measured from (*92*). *Abbreviations*: *L*, Length; *SL*, Segment Length.

Digits	*Bones*	*L (mm)*	*SL (mm)*	*Alligator (10.8%)*	*Struthio (6.8%)*	*Coturnix (1.8%)*
**DI**	MtI	180	278	30.02	18.90	5.00
	PhI-1	98	378	40.82	25.70	6.80
	PhI-2	280				
	Total		***656***	***70.85***	***44.608***	***11.808***
**DII**	MtII	220	340	36.72	23.12	6.12
	PhII-1	120	162	17.50	11.02	2.92
	PhII-2	42	252	27.22	17.14	4.54
	PhII-3	210				
	Total		***754***	***81.43***	***51.27***	***13.57***
**DIII**	MtIII	260	350	37.80	23.80	6.30
	PhIII-1	90	135	14.58	9.18	2.43
	PhIII-2	45	225	24.30	15.30	4.05
	PhIII-3	180				
	Total		***485***	***52.38***	***32.98***	***8.73***
**DIV**	MtIV	290	378	40.82	25.70	6.80
	PhIV-1	88	137	14.80	9.32	2.47
	PhIV-2	49				
	Total		***515***	***55.62***	***35.02***	***9.27***
**DV**	MtV	290	315	34.02	21.42	5.67
	PhV-1	25				
	Total			***34.02***	***21.42***	***5.67***
			**AVERAGE**	**28.96**	**18.24**	**4.83**
			**SD**	**10.05**	**6.33**	**1.68**

Table S5. Proportions of cartilage gaps for each digital segment in the pes of *Giraffatitan brancai* (?MB.R.5023), following measurements taken by AJ and the proportions measured from (*92*). *Abbreviations*: *L*, Length; *SL*, Segment Length.

Digits	*Bones*	*L (mm)*	*SL (mm)*	*Alligator (10.8%)*	*Struthio (6.8%)*	*Coturnix (1.8%)*
**DI**	MtI	200	290	31.32	19.72	5.22
	PhI-1	90	275	29.70	18.70	4.95
	PhI-2	185				
	Total		***565***	***61.02***	***38.42***	***10.17***
**DII**	MtII	250	340	36.72	23.12	6.12
	PhII-1	90	130	14.04	8.84	2.34
	PhII-2	40	185	19.98	12.58	3.33
	PhII-3	145				
	Total		***655***	***70.74***	***44.54***	***11.79***
**DIII**	MtIII	320	395	42.66	26.86	7.11
	PhIII-1	75	110	11.88	7.48	1.98
	PhIII-2	35				
	Total		***505***	***54.54***	***34.34***	***9.09***
**DIV**	MtIV	295	355	38.34	24.14	6.39
	PhIV-1	60	85	9.18	5.78	1.53
	PhIV-2	25				
	Total		***440***	***47.52***	***29.92***	***7.92***
**DV**	MtV	245	265	28.62	18.02	4.77
	PhV-1	20				
	Total			***28.62***	***18.02***	***4.77***
			**AVERAGE**	**26.24**	**16.52**	**4.37**
			**SD**	**11.81**	**7.43**	**1.97**

Table S6. Size and number of elements for the sensitivity analysis of varying mesh density, using metatarsal III of *Giraffatitan brancai* as an exemplar.

**Sensitivity models**	**Element size (mm)**	**Volume element size (mm)**	**Mid-digitigrade**	**Digitigrade**	**Subunguligrade**
			Number elements	Number elements	Number elements
1	2.5	2.5	1012	1012	974
2	2	2	1496	1494	1488
3	1.5	1.5	2456	2458	2466
4	1	1	5182	5232	5226
5	0.5	0.5	19804	19792	19766
6	0.25	0.25	78348	78244	78254
7	0.1	0.1	488688	487746	488942

Table S7. Body mass estimations for the specimens used in this study following the '*body mass scaling method*' from (*41*). Abbreviations: FC, Femur Circumference; HC, Humerus Circumference; W, Weight. Note that the body mass estimation for *Rhoetosaurus brownei* was based on the mean value of four distinct estimates of its humerus circumference (ranging from 450 to 600 mm; see Fig. S5).

**Specimens**		**FC** (mm)	**HC** (mm)	**FC+HC** (mm)	**logMass**	**Mass** (kg)	**Mass simplified** (kg)	**Weight** (N)	**¼ Weight** (N)	**¼ Weight simplified** (N)
Plateosaurus engelhardti		263.00	156.00	419.00	5.96	916.69	900.00	8825.99	2206.50	**2000.00**
Camarasaurus grandis		630.00	472.00	1102.00	7.26	18153.37	18000.00	176519.70	44129.93	**45000.00**
Diplodocus carnegii		591.00	406.00	997.00	7.14	13785.20	14000.00	137293.10	34323.28	**35000.00**
Giraffatitan brancai		730.00	654.00	1384.00	7.53	33961.21	34000.00	333426.10	83356.53	**85000.00**
Elephant		-	-	-	-	-	4000.00	39226.60	9806.65	**10000.00**
Rhoetosaurus brownei	*Estimate 1*	696.00	450.00	1146.00	7.31	20216.15	20000.00	196133.00	49033.25	
	Estimate 2	696.00	500.00	1196.00	7.36	22734.37	23000.00	225552.95	56388.24	
	Estimate 3	696.00	550.00	1246.00	7.41	25443.63	25000.00	245166.25	61291.56	
	Estimate 4	696.00	600.00	1296.00	7.45	28349.88	28000.00	274586.20	68646.55	
	Mean	696.00	525.00	1221.00	7.38	24064.75	24000.00	235359.60	58839.90	**60000.00**

Table S8. Results of generalized linear models (*glm*) using Tukey's HSD investigating whether a clear difference occurs between skeletal postures versus postures that include a hypothetical pad. (Significance codes: 0 '***' 0.001 '**' 0.01 '*' 0.05 '.' 0.1 ' ' 1). **Abbreviations: Px**, postural morphotypes of *Plateosaurus engelhardti*, including mid-digitigrady (P2), digitigrady (P3), and subunguligrady (P4); **Rx**, postural morphotypes of *Rhoetosaurus brownei,* including mid-digitigrady (R2), digitigrady (R3), and subunguligrady (R4); **Dx**, postural morphotypes of *Diplodocus carnegii,* including mid-digitigrady (D2), digitigrady (D3), and subunguligrady (D4); **Cx**, postural morphotypes of *Camarasaurus* sp., including mid-digitigrady (C2), digitigrady (C3), and subunguligrady (C4); **Rx**, postural morphotypes of *Giraffatitan brancai,* including mid-digitigrady (G2), digitigrady (G3), and subunguligrady (G4); **E**, the simulated elephant pes. **MT**, Metatarsals. **PH**, Phalanges.

SKELETAL-PAD		S.Mises					E.Max					E.Min				
		Estimate	Std. Error	z value	Pr(>|z|)		Estimate	Std. Error	z value	Pr(>|z|)		Estimate	Std. Error	z value	Pr(>|z|)	
P2	MT	-2.9870	0.00989	-301.9	<2e-16	***	-1.5571	0.00774	-201.1	<2e-16	***	1.6791	0.00800	209.9	<2e-16	***
	PH	-2.2858	0.00955	-239.4	<2e-16	***	-1.4213	0.00821	-173.1	<2e-16	***	1.0791	0.00740	145.8	<2e-16	***
P3	MT	-2.8844	0.00965	-298.9	<2e-16	***	-1.4456	0.00745	-194.2	<2e-16	***	1.6939	0.00916	184.9	<2e-16	***
	PH	-2.1317	0.01085	-196.4	<2e-16	***	-1.3166	0.00801	-164.4	<2e-16	***	1.0174	0.00928	109.6	<2e-16	***
P4	MT	-4.1046	0.01052	-390.2	<2e-16	***	-2.1108	0.00817	-258.3	<2e-16	***	2.3623	0.00930	254.0	<2e-16	***
	PH	-5.8442	0.02366	-247.0	<2e-16	***	-3.2177	0.02371	-135.7	<2e-16	***	3.1549	0.01511	208.7	<2e-16	***
R2	MT	-16.4736	0.01264	-1303.0	<2e-16	***	-8.3277	0.00908	-917.0	<2e-16	***	9.5371	0.01252	761.9	<2e-16	***
	PH	-1.9895	0.00278	-714.6	<2e-16	***	-1.5296	0.00242	-632.8	<2e-16	***	0.6977	0.00131	530.8	<2e-16	***
R3	MT	-11.7242	0.01195	-980.8	<2e-16	***	-5.5530	0.00827	-671.7	<2e-16	***	7.1330	0.01026	695.5	<2e-16	***
	PH	-0.8127	0.00212	-382.6	<2e-16	***	-0.6377	0.00186	-342.5	<2e-16	***	0.2670	0.00114	233.8	<2e-16	***
R4	MT	-17.8026	0.01279	-1392.0	<2e-16	***	-8.2701	0.00966	-856.0	<2e-16	***	11.0885	0.01127	983.9	<2e-16	***
	PH	-20.8828	0.03435	-607.9	<2e-16	***	-11.6394	0.02503	-465.0	<2e-16	***	11.2115	0.02803	399.9	<2e-16	***
D2	MT	-6.6165	0.01296	-510.7	<2e-16	***	-3.3161	0.00844	-393.0	<2e-16	***	3.8918	0.01193	326.2	<2e-16	***
	PH	-1.1705	0.00301	-389.2	<2e-16	***	-0.9187	0.00261	-351.8	<2e-16	***	0.3718	0.00154	241.9	<2e-16	***
D3	MT	-6.7328	0.01658	-406.0	<2e-16	***	-3.1465	0.01265	-248.7	<2e-16	***	4.2351	0.01599	264.9	<2e-16	***
	PH	-1.1930	0.00356	-335.5	<2e-16	***	-0.8524	0.00296	-287.7	<2e-16	***	0.4664	0.00221	210.9	<2e-16	***
D4	MT	-7.1406	0.01730	-412.7	<2e-16	***	-3.2336	0.01284	-251.8	<2e-16	***	4.5248	0.01426	317.2	<2e-16	***
	PH	-4.5947	0.00780	-589.3	<2e-16	***	-2.6588	0.00607	-437.7	<2e-16	***	2.4047	0.00618	389.2	<2e-16	***
C2	MT	-4.3806	0.00654	-669.7	<2e-16	***	-2.1262	0.00474	-448.2	<2e-16	***	2.6596	0.00578	460.5	<2e-16	***
	PH	-0.6554	0.00413	-158.8	<2e-16	***	-0.5582	0.00380	-146.8	<2e-16	***	0.1675	0.00260	64.5	<2e-16	***
C3	MT	-4.2183	0.00744	-567.0	<2e-16	***	-1.9428	0.00456	-426.4	<2e-16	***	2.6796	0.00684	391.6	<2e-16	***
	PH	-0.6485	0.00277	-234.5	<2e-16	***	-0.5690	0.00279	-204.1	<2e-16	***	0.1470	0.00113	130.7	<2e-16	***
C4	MT	-4.8820	0.01100	-443.9	<2e-16	***	-2.2796	0.00629	-362.2	<2e-16	***	3.0460	0.01097	277.6	<2e-16	***
	PH	-1.3127	0.00343	-382.8	<2e-16	***	-1.0355	0.00325	-319.0	<2e-16	***	0.3980	0.00177	225.2	<2e-16	***
G2	MT	-17.6810	0.02566	-689.0	<2e-16	***	-8.3016	0.01865	-445.0	<2e-16	***	10.6197	0.01845	575.5	<2e-16	***
	PH	-23.2211	0.05155	-450.5	<2e-16	***	-12.6419	0.03653	-346.1	<2e-16	***	12.7845	0.05029	254.2	<2e-16	***
G3	MT	-12.3613	0.01083	-1141.0	<2e-16	***	-5.5077	0.00807	-682.4	<2e-16	***	7.8732	0.00944	833.8	<2e-16	***
	PH	-24.1032	0.05403	-446.1	<2e-16	***	-14.1870	0.04300	-329.9	<2e-16	***	12.1496	0.04576	265.5	<2e-16	***
G4	MT	-14.9485	0.01213	-1233.0	<2e-16	***	-7.0691	0.00937	-754.3	<2e-16	***	8.8677	0.01078	822.8	<2e-16	***
	PH	-48.5002	0.11330	-428.0	<2e-16	***	-28.9720	0.12200	-237.4	<2e-16	***	24.0210	0.07889	304.5	<2e-16	***
E	MT	-1.5837	0.00182	-872.2	<2e-16	***	-0.7789	0.00139	-560.5	<2e-16	***	0.9243	0.00177	521.6	<2e-16	***
	PH	-3.7186	0.03988	-93.3	<2e-16	***	-2.0989	0.01301	-161.4	<2e-16	***	1.9522	0.04402	44.4	<2e-16	***

Table S9. Information sources for all taxa illustrated in the article.

Taxon	References	Notes
Alamosaurus sanjuanensis	(*114*)	
Antarctosaurus wichmannianus	(*115*)	
Apatosaurus louisae	(*116*); (*117*)	↔
Barosaurus "africanus"	(*118*)	↔
Blikanasaurus cromptoni	(*119*)	↔
Camarasaurus lentus	(*120*)	↔
Cedarosaurus weiskopfae	(*121*); (*122*)	↔
Cetiosauriscus stewarti	(*123*)	↔
Diplodocus carnegii	(*1*)	
Dyslocosaurus polyonychius	(*124*)	↔
Eosauropus	(*68*)	
Epachthosaurus sciuttoi	(*125*)	*
Euhelopus zdanskyi	(*126*)	
Galeamopus pabsti	(*127*)	*, ↔
Giraffatitan brancai	(*118*)	
Gobititan shenzouensis	(*128*)	↔
Gongxianosaurus shibeiensis	(*129*)	
Haplocanthosaurus sp.	(*130*)	
Janenschia robusta	(*131*)	
Kunmingosaurus wudingensis	(*132*)	
La Invernada	(*133*)	↔
Mamenchisaurus hochuanensis	(*134*)	
Mendozasaurus neguyelap	(*135*)	
Mierasaurus bobyoungi	(*136*)	↔
Notocolossus gonzalezparejasi	(*18*)	
Omeisaurus tianfuensis	(*137*)	↔
Opisthocoelicaudia skarzynskii	(*138*)	↔
Plateosaurus engelhardti	(*139*)	↔
Rhoetosaurus brownei	(*17*)	
Shunosaurus lii	(*87*); (*40*)	↔
Sonorasaurus thompsoni	(*140*)	↔
Tangvayosaurus hoffeti	(*141*) ; The Savannakhet Dinosaur Museum website	*
Tastavinsaurus sanzi	(*142*)	↔
Tazoudasaurus naimi	(*64*)	
Turiasaurus riodevensis	(*143*)	
Vulcanodon karibaensis	(*144*)	↔
Evazoum siriguii	(*58*)	
Pseudotetrasauropus bipedoida	(*63*)	
Lavinipes cheminii	(*145*)	
Kalosauropus pollex	(*62*)	
Liujianpus shunan	(*69*)	↔
Polyonyx gomesi	(*146*)	
Parabrontopodus mcintoshi	(*70*)	
Brontopodus birdi	(*36*)	
Titanopodus mendozensis	(*147*)	
Unnamed Asian track	(*148*)	

↔ Mirrored outlines originally left pes (for ease of comparisons); * Pes rearticulated in this study (for comparative purposes)

Data S1. Material properties attributed to Finite element models of living and extinct taxa in the literature.

See the separate file "***Data S1 Material properties attributed to FEM in the literature***" in the spreadsheet format ***.xlsx**

Data S2. Review of the Sauropodomorpha pedal body and trace fossils from the Late Triassic-Early Jurassic.

See the separate file "***Data S2 Sauropodomorpha Pedal Body Trace Fossil Record Late Triassic Early Jurassic***" in the spreadsheet format ***.xlsx**

Movie S1. Animated FEA results for *Plateosaurus engelhardti*.

See the separate file "***Jannel et al Softening the steps to gigantism in sauropod dinosaurs through the evolution of a pedal pad Movie 1 - Plateosaurus engelhardti"*** in the ***.mp4** format.

Movie S2. Animated FEA results for *Diplodocus carnegii*.

See the separate file "***Jannel et al Softening the steps to gigantism in sauropod dinosaurs through the evolution of a pedal pad Movie 2 - Diplodocus carnegii"*** in the ***.mp4** format.

Movie S3. Animated FEA results for *Rhoetosaurus brownei*.

See the separate file "***Jannel et al Softening the steps to gigantism in sauropod dinosaurs through the evolution of a pedal pad Movie 3 - Rhoetosaurus brownei"*** in the ***.mp4** format.

Movie S4. Animated FEA results for *Camarasaurus* sp.

See the separate file "***Jannel et al Softening the steps to gigantism in sauropod dinosaurs through the evolution of a pedal pad Movie 4 - Camarasaurus sp"*** in the ***.mp4** format.

Movie S5. Animated FEA results for *Giraffatitan brancai*.

See the separate file "***Jannel et al Softening the steps to gigantism in sauropod dinosaurs through the evolution of a pedal pad Movie 5 - Giraffatitan brancai"*** in the ***.mp4** format.

Movie S6. Animated FEA results for the simulated elephant pes.

See the separate file "***Jannel et al Softening the steps to gigantism in sauropod dinosaurs through the evolution of a pedal pad Movie 6 - Simulated Elephant pes"*** in the ***.mp4** format.

REFERENCES AND NOTES

1. J. B. Hatcher, *Diplodocus* (Marsh): Its osteology, taxonomy, and probable habits, with a restoration of the skeleton. *Mem. Carnegie Mus.* **1**, 1–63 (1901).

2. F. von Huene, Ueber einen sauropoden im obern Malm des Berner Jura. *Swiss J. Geosci.* **17**, 80–94 (1922).

3. R. T. Bird, We captured a 'live' brontosaur. *Nat. Geo.* **105**, 707–722 (1954).

4. R. T. Bakker, "Dinosaur heresy—Dinosaur renaissance: Why we need endothermic archosaurs for a comprehensive theory of bioenergetic evolution," in *A Cold Look at the Warm-Blooded Dinosaurs,* R. D. K. Thomas, E. C. Olson, Eds. (American Association for the Advancement of Science, Selected Symposia Series, Westview Press, 1980), vol. 28, pp. 351–462.

5. P. M. Sander, A. Christian, M. Clauss, R. Fechner, C. T. Gee, E.-M. Griebeler, H.-C. Gunga, J. Hummel, H. Mallison, S. F. Perry, H. Preuschoft, O. W. M. Rauhut, K. Remes, T. Tütken, O. Wings, U. Witzel, Biology of the sauropod dinosaurs: The evolution of gigantism. *Biol. Rev.* **86**, 117–155 (2011).

6. N. Klein, K. Remes, C. T. Gee, P. M. Sander, *Biology of the Sauropod Dinosaurs: Understanding the Life of Giants* (Life of the Past, Indiana Univ. Press, 2011), 331 pp.

7. K. A. Curry Rogers, J. A. Wilson, Eds., *The Sauropods: Evolution and Paleobiology* (University of California Press, 2005), 349 pp.

8. R. M. Alexander, "Body support, scaling, and allometry," in *Functional Vertebrate Morphology,* M. Hildebrand, D. M. Bramble, K. F. Liem, D. B. Wake, Eds. (The Belknap Press of Harvard Univ. Press, 1985), chap. 2, pp. 26–37.

9. A. A. Biewener, Scaling body support in mammals: Limb posture and muscle mechanics. *Science* **245**, 45–48 (1989).

10. A. A. Biewener, Mammalian terrestrial locomotion and size. *Bioscience* **39**, 776–783 (1989).

11. P. Upchurch, The evolutionary history of sauropod dinosaurs. *Philos. Trans. R. Soc. Lond. B Biol. Sci.* **349**, 365–390 (1995).

12. H. Preuschoft, B. Hohn-Schulte, S. Stoinski, U. Witzel, "Why so huge? Biomechanical reasons for the acquisition of large size in sauropod and theropod dinosaurs," in *Biology of the Sauropod Dinosaurs: Understanding the Life of Giants*, N. Klein, K. Remes, C. T. Gee, P. M. Sander, Eds. (Life of the Past, Indiana Univ. Press, 2011), chap. 12, pp. 197–218.

13. P. M. Sander, An evolutionary cascade model for sauropod dinosaur gigantism—Overview, update and tests. *PLOS One* **8**, e78573 (2013).

14. P. Christiansen, Locomotion in sauropod dinosaurs. *Gaia* **14**, 45–75 (1997).

15. J. A. Wilson, M. T. Carrano, Titanosaurs and the origin of "wide-gauge" trackways: A biomechanical and systematic perspective on sauropod locomotion. *Paleobiology* **25**, 252–267 (1999).

16. M. T. Carrano, "The evolution of sauropod locomotion: Morphological diversity of a secondarily quadrupedal radiation," in *The Sauropods: Evolution and Paleobiology*, K. A. Curry Rogers, J. A. Wilson, Eds. (University of California Press, 2005), chap. 8, pp. 229–251.

17. J. P. Nair, S. W. Salisbury, New anatomical information on *Rhoetosaurus brownei* Longman, 1926, a gravisaurian sauropodomorph dinosaur from the Middle Jurassic of Queensland, Australia. *J. Vertebr. Paleontol.* **32**, 369–394 (2012).

18. B. J. G. Riga, M. C. Lamanna, L. D. Ortíz David, J. P. Coria, A gigantic new dinosaur from Argentina and the evolution of the sauropod hind foot. *Sci. Rep.* **6**, 19165 (2016).

19. M. Clauss, P. Steuer, D. W. H. Müller, D. Codron, J. Hummel, Herbivory and body size: Allometries of diet quality and gastrointestinal physiology, and implications for herbivore ecology and dinosaur gigantism. *PLOS ONE* **8**, e68714 (2013).

20. S. F. Perry, A. Christian, T. Breuer, N. Pajor, J. R. Codd, Implications of an avian-style respiratory system for gigantism in sauropod dinosaurs. *J. Exp. Zool. A Ecol. Genet. Physiol.* **311A**, 600–610 (2009).

21. P. D. Mannion, P. Upchurch, Completeness metrics and the quality of the sauropodomorph fossil record through geological and historical time. *Paleobiology* **36**, 283–302 (2010).

22. J. R. Hutchinson, C. Delmer, C. E. Miller, T. Hildebrandt, A. A. Pitsillides, A. Boyde, From flat foot to fat foot: Structure, ontogeny, function, and evolution of Elephant "sixth toes". *Science* **334**, 1699–1703 (2011).

23. O. Panagiotopoulou, T. C. Pataky, M. Day, M. C. Hensman, S. Hensman, J. R. Hutchinson, C. J. Clemente, Foot pressure distributions during walking in African elephants (*Loxodonta africana*). *R. Soc. Open Sci.* **3**, 160203 (2016).

24. O. Panagiotopoulou, T. C. Pataky, J. R. Hutchinson, Foot pressure distribution in White Rhinoceroses (*Ceratotherium simum*) during walking. *PeerJ* **7**, e6881 (2019).

25. C. J. Clemente, T. J. M. Dick, C. L. Glen, O. Panagiotopoulou, Biomechanical insights into the role of foot pads during locomotion in camelid species. *Sci. Rep.* **10**, 3856 (2020).

26. O. Panagiotopoulou, T. C. Pataky, Z. Hill, J. R. Hutchinson, Statistical parametric mapping of the regional distribution and ontogenetic scaling of foot pressures during walking in Asian elephants (*Elephas maximus*). *J. Exp. Biol.* **215**, 1584–1593 (2012).

27. O. Panagiotopoulou, J. W. Rankin, S. M. Gatesy, J. R. Hutchinson, A preliminary case study of the effect of shoe-wearing on the biomechanics of a horse's foot. *PeerJ* **4**, e2164 (2016).

28. M. F. Bonnan, "Pes anatomy in sauropod dinosaurs: Implications for functional morphology, evolution, and phylogeny," in *Thunder-Lizards: The Sauropodomorph Dinosaurs*, V. Tidwell, K. Carpenter, Eds. (Life of the Past, Indiana Univ. Press, 2005), chap. 16, pp. 346–380.

29. A. Jannel, J. P. Nair, O. Panagiotopoulou, A. Romilio, S. W. Salisbury, "Keep your feet on the ground": Simulated range of motion and hind foot posture of the Middle Jurassic sauropod *Rhoetosaurus brownei* and its implications for sauropod biology. *J. Morphol.* **280**, 849–878 (2019).

30. J. A. Wilson, P. C. Sereno, Early evolution and higher-level phylogeny of sauropod dinosaurs. *J. Vertebr. Paleontol.* **5**, 1–79 (1998).

31. B. G. Lovegrove, M. O. Mowoe, The evolution of mammal body sizes: Responses to Cenozoic climate change in North American mammals. *J. Evol. Biol.* **26**, 1317–1329 (2013).

32. S. M. Reilly, E. J. McElroy, A. R. Biknevicius, Posture, gait and the ecological relevance of locomotor costs and energy-saving mechanisms in tetrapods. *Zoology* **110**, 271–289 (2007).

33. S. C. Wearing, J. E. Smeathers, The heel fat pad: Mechanical properties and clinical applications. *J. Foot Ankle Res.* **4**, 114 (2011).

34. G. E. Weissengruber, G. F. Egger, J. R. Hutchinson, H. B. Groenewald, L. Elasser, D. J. Famini, G. Forstenpointner, The structure of the cushions in the feet of African elephants (*Loxodonta africana*). *J. Anat.* **209**, 781–792 (2006).

35. K. Moreno, M. T. Carrano, R. Snyder, Morphological changes in pedal phalanges through ornithopod dinosaur evolution: A biomechanical approach. *J. Morphol.* **268**, 50–63 (2007).

36. J. O. Farlow, J. G. Pittman, J. M. Hawthorne, "*Brontopodus birdi*, Lower Cretaceous sauropod footprints from the U.S. Gulf Coastal Plain," in *Dinosaur Tracks and Traces*, D. D. Gillette, M. G. Lockley, Eds. (Cambridge Univ. Press, 1989), chap. 42, pp. 371–394.

37. T. Thulborn, *Dinosaur Tracks* (Chapman and Hall, 1990), 410 pp.

38. L. A. Parry, F. M. Smithwick, K. K. Norden, E. T. Saitta, J. Lozano-Fernandez, A. R. Tanner, J.-B. Caron, G. D. Edgecombe, D. E. G. Briggs, J. Vinther, Soft-bodied fossils are not simply rotten carcasses – Toward a holistic understanding of exceptional fossil preservation. *Bioessays* **40**, 1700167 (2018).

39. D. Marty, "Sedimentology, taphonomy, and ichnology of Late Jurassic dinosaur tracks from the Jura carbonate platform (Chevenez—Combe Ronde tracksite, NW Switzerland): Insights into the tidal-flat palaeoenvironment and dinosaur diversity, locomotion, and palaeoecology," thesis, Université de Fribourg (2008).

40. P. Upchurch, P. M. Barrett, P. Dodson, "Sauropoda," in *The Dinosauria (2nd Edition)*, D. B. Weishampel, P. Dodson, H. Osmólska, Eds. (University of California Press, 2004), pp. 259–322.

41. R. B. J. Benson, N. E. Campione, M. T. Carrano, P. D. Mannion, C. S. Sullivan, P. Upchurch, D. C. Evans, Rates of dinosaur body mass evolution indicate 170 million years of sustained ecological innovation on the avian stem lineage. *PLOS Biol.* **12**, e1001853 (2014).

42. J. P. Bilezikian, L. G. Raisz, T. J. Martin, *Principles of Bone Biology, Third Edition: Two-Volume Set* (Academic Press, 2008), vol. 1 and 2.

43. A. J. de Ricqlès, "Tissue structures of dinosaur bone—Functional significance and possible relation to dinosaur physiology," in *A Cold Look at the Warm-Blooded Dinosaurs*, R. D. K. Thomas, E. C. Olson, Eds. (American Association for the Advancement of Science, Selected Symposia Series, Westview Press, 1980), vol. 28, pp. 103–139.

44. K. N. Chethan, S. N. Bhat, M. Zuber, S. B. Shenoy, Patient-specific static structural analysis of femur bone of different lengths. *Open Biomed. Eng. J.* **12**, 108–114 (2018).

45. J. D. Currey, Mechanical properties of vertebrate hard tissues. *Proc. Inst. Mech. Eng. H* **212**, 399–411 (1998).

46. R. M. Alexander, M. R. Bennett, R. F. Ker, Mechanical properties and function of the paw pads of some mammals. *J. Zool.* **209**, 405–419 (1986).

47. J. E. Miller-Young, N. A. Duncan, G. Baroud, Material properties of the human calcaneal fat pad in compression: Experiment and theory. *J. Biomech.* **35**, 1523–1531 (2002).

48. M. Räber, C. J. Lischer, H. Geyer, P. Ossent, The bovine digital cushion—A descriptive anatomical study. *Vet. J.* **167**, 258–264 (2004).

49. M. Egerbacher, M. Helmreich, A. Probst, P. Böck, Digital cushions in horses comprise coarse connective tissue, myxoid tissue, and cartilage but only little unilocular fat tissue. *Anat. Histol. Embryol.* **34**, 112–116 (2005).

50. B. M. Rothschild, R. E. Molnar, "Sauropod stress fractures as clues to activity," in *Thunder-Lizards: The Sauropodomorph Dinosaurs,* V. Tidwell, K. Carpenter, Eds. (Life of the Past, Indiana Univ. Press, 2005), chap. 17, pp. 381–392.

51. E. Tschopp, O. Wings, T. Frauenfelder, B. M. Rothschild, Pathological phalanges in a camarasaurid sauropod dinosaur and implications on behaviour. *Acta Palaeontol. Pol.* **61**, 125–134 (2016).

52. G. H. Evans, *Elephants and Their Diseases* (Superintendent, Government Printing, 1910).

53. M. E. Fowler, "An overview of foot conditions in Asian and African elephants," in *The Elephant's Foot: Prevention and Care of Foot Conditions in Captive Asian and African Elephants,* B. Csuti, E. L. Sargent, U. S. Bechert, Eds. (Iowa State Univ. Press, 2001), chap. 1, pp. 3–7.

54. R. J. Dudley, S. P. Wood, J. R. Hutchinson, R. Weller, Radiographic protocol and normal anatomy of the hind feet in the white rhinoceros (*Ceratotherium simum*). *Vet. Radiol. Ultrasound* **56**, 124–132 (2015).

55. K. T. Bates, R. Savage, T. C. Pataky, S. A. Morse, E. Webster, P. L. Falkingham, L. Ren, Z. Qian, D. Collins, M. R. Bennett, J. McClymont, R. H. Crompton, Does footprint depth correlate with foot motion and pressure? *J. R. Soc. Interface* **10**, 20130009 (2013).

56. R. N. Martínez, O. A. Alcober, A basal sauropodomorph (Dinosauria: Saurischia) from the ischigualasto formation (Triassic, Carnian) and the early evolution of Sauropodomorpha. *PLOS ONE* **4**, e4397 (2009).

57. H. Mallison, The digital *Plateosaurus* II: An assessment of the range of motion of the limbs and vertebral column and of previous reconstructions using a digital skeletal mount. *Acta Palaeontol. Pol.* **55**, 433–458 (2010).

58. U. Nicosia, M. Loi, Triassic footprints from Lerici (La Spezia, Northern Italy). *Ichnos* **10**, 127–140 (2003).

59. M. G. Lockley, S. G. Lucas, A. P. Hunt, *Evazoum* and the renaming of northern hemisphere "*Pseudotetrasauropus*": Implications for the tetrapod ichnotaxonomy at the Triassic-Jurassic boundary. *N. M. Mus. Nat. Hist. Sci. Bull.* **37**, 199–206 (2006).

60. A. Romilio, H. Klein, A. Jannel, S. W. Salisbury, Saurischian dinosaur tracks from the Upper Triassic of southern Queensland: Possible evidence for Australia's earliest sauropodomorph trackmaker. *Hist. Biol.*, 1–10 (2021).

61. E. C. Rainforth, Revision and re-evaluation of the Early Jurassic dinosaurian ichnogenus *Otozoum*. *Palaeontology* **46**, 803–838 (2003).

62. R. Mukaddam, E. M. Bordy, M. G. Lockley, K. E. J. Chapelle, Reviving *Kalosauropus*, an Early Jurassic sauropodomorph track from southern Africa (Lesotho). *Hist. Biol.* **33**, 2908–2930 (2021).

63. S. D'Orazi Porchetti, U. Nicosia, Re-examination of some large early Mesozoic tetrapod footprints from the African collection of Paul Ellenberger. *Ichnos* **14**, 219–245 (2007).

64. R. Allain, N. Aquesbi, Anatomy and phylogenetic relationships of *Tazoudasaurus naimi* (Dinosauria, Sauropoda) from the late Early Jurassic of Morocco. *Geodiversitas* **30**, 345–424 (2008).

65. M. R. Cooper, A reassessment of *Vulcanodon karibaensis* Raath (Dinosauria: Saurischia) and the origin of the Sauropoda. *Palaeontol. Afr.* **25**, 203–231 (1984).

66. B. McPhee, R. B. J. Benson, J. Botha-Brink, E. M. Bordy, J. N. Choiniere, A giant dinosaur from the earliest Jurassic of South Africa and the transition to quadrupedality in early sauropodomorphs. *Curr. Biol.* **28**, 3143–3151.e7 (2018).

67. C. Apaldetti, R. N. Martínez, I. A. Cerda, D. Pol, O. Alcober, An early trend towards gigantism in Triassic sauropodomorph dinosaurs. *Nat. Ecol. Evol.* **2**, 1227–1232 (2018).

68. J. N. Lallensack, H. Klein, J. Milàn, O. Wings, O. Mateus, L. B. Clemmensen, Sauropodomorph dinosaur trackways from the fleming fjord formation of East Greenland: Evidence for Late Triassic sauropods. *Acta Palaeontol. Pol.* **62**, 833–843 (2017).

69. L. Xing, M. G. Lockley, J. Zhang, H. Klein, D. Li, T. Miyashita, Z. Li, S. B. Kümmell, A new sauropodomorph ichnogenus from the Lower Jurassic of Sichuan, China fills a gap in the track record. *Hist. Biol.* **28**, 881–895 (2016).

70. M. G. Lockley, J. O. Farlow, C. A. Meyer, *Brontopodus* and *Parabrontopodus* ichnogen. nov. and the significance of wide- and narrow-gauge sauropod trackways. *Gaia* **10**, 135–146 (1994).

71. C. Brassey, L. Margetts, A. C. Kitchener, P. J. Withers, P. L. Manning, W. I. Sellers, Finite element modelling versus classic beam theory: Comparing methods for stress estimation in a morphologically diverse sample of vertebrate long bones. *J. R. Soc. Interface* **10**, 20120823 (2013).

72. N. Court, Limb posture and gait in *Numidotherium koholense*, a primitive proboscidean from the Eocene of Algeria. *Zool. J. Linn. Soc.* **111**, 297–338 (1994).

73. P. L. Falkingham, Acquisition of high resolution three-dimensional models using free, open-source, photogrammetric software. *Palaeontol. Electron.* **15**, 15 (2012).

74. H. Mallison, O. Wings, Photogrammetry in paleontology—A practical guide. *J. Paleontol. Tech.* **12**, 1–31 (2012).

75. K. A. Curry Rogers, Ontogenetic histology of Apatosaurus (Dinosauria: Sauropoda): New insights on growth rates and longevity. *J. Vertebr. Paleontol.* **19**, 654–665 (1999).

76. R. E. H. Reid, Bone histology of the Cleveland-Lloyd dinosaurs and of dinosaurs in general, part 1: Introduction: Introduction to bone tissues. *BYU Geol. Stud.* **41**, 25–71 (1996).

77. E. J. Rayfield, D. B. Norman, C. C. Horner, J. R. Horner, P. M. Smith, J. J. Thomason, P. Upchurch, Cranial design and function in a large theropod dinosaur. *Nature* **409**, 1033–1037 (2001).

78. B. K. Hall, *Bones and Cartilage: Developmental and Evolutionary Skeletal Biology* (Elsevier, 2005), 760 pp.

79. G. Zhang, B. F. Eames, M. J. Cohn, Evolution of vertebrate cartilage development. *Curr. Top. Dev. Biol.* **86**, 15–42 (2009).

80. R. F. Ker, The design of soft collagenous load-bearing tissues. *J. Exp. Biol.* **202**, 3315–3324 (1999).

81. P. M. Galton, The prosauropod dinosaur *Plateosaurus* (*Dimodosaurus*) *poligniensis* (Pidancet & Chopard, 1862) (Upper Triassic, Poligny, France). *Neues Jahrb. Geol. Palaontol. Abh.* **207**, 255–288 (1998).

82. H. A. Longman, A giant dinosaur from Durham Downs, Queensland. *Mem. Queensl. Mus.* **8**, 183–194 (1926).

83. J. H. Ostrom, J. S. McIntosh, *Marsh's Dinosaurs: The Collections from Como Bluff* (Yale Univ. Press, 1966), 388 pp.

84. G. S. Paul, The brachiosaur giants of the Morrison and Tendaguru with a description of a new subgenus, *Giraffatitan*, and a comparison of the world's largest dinosaurs. *Hunteria* **2**, 1–14 (1988).

85. M. P. Taylor, A re-evaluation of *Brachiosaurus altithorax* Riggs 1903 (Dinosauria, Sauropoda) and its generic separation from *Giraffatitan brancai* (Janensch 1914). *J. Vertebr. Paleontol.* **29**, 787–806 (2009).

86. X. He, K. Li, K. Cai, Y. Gao, *Omeisaurus tianfuensis*—A new species of *Omeisaurus* from Dashanpu, Zigong, Sichuan. *J. Chengdu College Geol.* **2**, 15–32 (1984).

87. Y. H. Zhang, *The Middle Jurassic Dinosaurian Fauna from Dashanpu, Zigong, Sichuan. III. The Sauropod Dinosaurs 1. Shunosaurus* (Sichuan Publishing House of Science and Technology, 1988), vol. 3, 89 pp.

88. B. Csuti, E. L. Sargent, U. S. Bechert, *The Elephant's Foot: Prevention and Care of Foot Conditions in Captive Asian and African Elephants* (Iowa State Univ. Press, 2001), 163 pp.

89. E. C. Ramsay, R. W. Henry, “Anatomy of the elephant foot,” in *The Elephant's Foot: Prevention and Care of Foot Conditions in Captive Asian and African Elephants*, B. Csuti, E. L. Sargent, U. S. Bechert, Eds. (Iowa State Univ. Press, 2001), chap. 2, pp. 9–12.

90. G. E. Weissengruber, G. Forstenpointner, Musculature of the crus and pes of the African elephant (*Loxodonta africana*): Insight into semiplantigrade limb architecture. *Anat. Embryol.* **208**, 451–461 (2004).

91. S. J. Hall, *Basic Biomechanics* (McGraw-Hill Education, 2012).

92. C. M. Holliday, R. C. Ridgely, J. C. Sedlmayr, L. M. Witmer, Cartilaginous epiphyses in extant archosaurs and their implications for reconstructing limb function in dinosaurs. *PLOS ONE* **5**, e13120 (2010).

93. J. Dushoff, M. P. Kain, B. M. Bolker, I can see clearly now: Reinterpreting statistical significance. *Methods Ecol. Evol.* **10**, 756–759 (2019).

94. G. F. Egger, K. Witter, G. E. Weissengruber, G. Forstenpointner, Articular cartilage in the knee joint of the African Elephant, *Loxodonta africana*, Blumenbach 1797. *J. Morphol.* **269**, 118–127 (2008).

95. J. Malda, J. C. Grauw, K. E. M. Benders, M. J. L. Kik, C. H. A. van de Lest, L. B. Creemers, W. J. A. Dhert, P. R. van Weeren, Of mice, men and elephants: The relation between articular cartilage thickness and body mass. *PLOS ONE* **8**, e57683 (2013).

96. L. E. Hall, A. E. Fragomeni, D. W. Fowler, “The flexion of sauropod pedal unguals and testing the substrate grip hypothesis using the trackway fossil record,” in *Dinosaur Tracks: The Next Steps*, P. L. Falkingham, D. Marty, A. Richter, Eds. (Life of the Past, Indiana Univ. Press, 2016), chap. 9, pp. 138–151.

97. A. Benz, “The elephant's hoof: Macroscopic and microscopic morphology of defined locations under consideration of pathological changes,” thesis, University of Zurich (2005).

98. S. A. A. El-Gendy, A. Derbalah, M. E. R. A. El-Magd, Histo-morphological study on the footpad of ostrich (*Struthio camelus*) in relation to locomotion. *J. Vet. Anat.* **4**, 77–97 (2011).

99. R. Zhang, D. Han, S. Ma, G. Luo, Q. Ji, S. Xue, M. Yang, J. Li, Plantar pressure distribution of ostrich during locomotion on loose sand and solid ground. *PeerJ* **5**, e3613 (2017).

100. R. Zhang, L. Ling, D. Han, H. Wang, G. Yu, L. Jiang, D. Li, Z. Chang, FEM analysis in excellent cushion characteristic of ostrich (*Struthio camelus*) toe pads. *PLOS ONE* **14**, e0216141 (2019).

101. M. E. Fowler, Comparative clinical anatomy of ratites. *J. Zoo Wildl. Med.* **22**, 204–227 (1991).

102. J. A. Bright, E. J. Rayfield, The response of cranial biomechanical finite element models to variations in mesh density. *Anat. Rec.* **294**, 610–620 (2011).

103. J. A. Bright, A review of paleontological finite element models and their validity. *J. Paleo.* **88**, 760–769 (2014).

104. W.-P. Cheng, F.-T. Tang, C.-W. Ju, Stress distribution of the foot during mid-stance to push-off in barefoot gait: A 3-D finite element analysis. *Clin. Biomech.* **16**, 614–620 (2001).

105. J. T.-M. Cheung, M. Zhang, A 3-dimensional finite element model of the human foot and ankle for insole design. *Arch. Phys. Med. Rehabil.* **86**, 353–358 (2005).

106. H.-Y. K. Cheng, C.-L. Lin, H.-W. Wang, S.-W. Chou, Finite element analysis of plantar fascia under stretch—The relative contribution of windlass mechanism and Achilles tendon force. *J. Biomech.* **41**, 1937–1944 (2008).

107. D. D. Taylor, D. M. Hood, G. D. Potter, H. A. Hogan, C. M. Honnas, Evaluation of displacement of the digital cushion in response to vertical loading in equine forelimbs. *Am. J. Vet. Res.* **66**, 623–629 (2005).

108. A. Benz, W. Zenker, T. B. Hildebrandt, G. Weissengruber, K. Eulenberger, H. Geyer, Microscopic morphology of the elephant's hoof. *J. Zoo Wildl. Med.* **40**, 711–725 (2009).

109. A. Gefen, M. Megido-Ravid, Y. Itzchak, In vivo biomechanical behavior of the human heel pad during the stance phase of gait. *J. Biomech.* **34**, 1661–1665 (2001).

110. K.-J. Chi, V. L. Roth, Scaling and mechanics of carnivoran footpads reveal the principles of footpad design. *J. R. Soc. Interface* **7**, 1145–1155 (2010).

111. R. M. Alexander, Mechanics of posture and gait of some large dinosaurs. *Zool. J. Linn. Soc.* **83**, 1–25 (1985).

112. R. C. McClure, *Functional Anatomy of the Horse Foot* (University of Missouri, 1993), pp. 1–2.

113. A. B. Clifford, The evolution of the unguligrade manus in artiodactyls. *J. Vertebr. Paleontol.* **30**, 1827–1839 (2010).

114. M. D. D'Emic, J. A. Wilson, T. E. Williamson, A sauropod dinosaur pes from the latest Cretaceous of North America and the validity of *Alamosaurus sanjuanensis* (Sauropoda, Titanosauria). *J. Vertebr. Paleontol.* **31**, 1072–1079 (2011).

115. F. F. von Huene, Los saurisquios y ornitisquios del Cretaceo Argentino. *Anales del Museo de la Plata (Series 2)* **3**, 1–194 (1929).

116. J. B. Hatcher, Structure of the fore limb and manus of *Brontosaurus*. *Ann. Carnegie Mus.* **1**, 356–376 (1902).

117. W. P. Coombs Jr., Sauropod habits and habitats. *Palaeogeogr. Palaeoclimatol. Palaeoecol.* **17**, 1–33 (1975).

118. W. Janensch, Die gliedmaszen und gliedmaszengurtel der sauropoden der Tendaguru-Schichten. *Palaeontographica (Supplement 7)* **3**, 177–235 (1961).

119. P. M. Galton, J. W. A. van Heerden, Partial hindlimb of *Blikanasaurus cromptoni* n. gen. and n. sp., representing a new family of prosauropod dinosaurs from the Upper Triassic of South Africa. *Geobios* **18**, 509–516 (1985).

120. M. F. Bonnan, The evolution of manus shape in sauropod dinosaurs: Implications for functional morphology, forelimb orientation, and phylogeny. *J. Vertebr. Paleontol.* **23**, 595–613 (2003).

121. M. R. Gallup, Functional morphology of the hindfoot of the Texas sauropod *Pleurocoelus* sp. indet. *Geol. Soc. Am. Spec. Pap.* **238**, 71–74 (1989).

122. M. D. D'Emic, Revision of the sauropod dinosaurs of the lower cretaceous trinity group, southern USA, with the description of a new genus. *J. Syst. Palaeontol.* **11**, 707–726 (2013).

123. J. F. Heathcote, "The anatomy and phylogeny of *Cetiosauriscus stewarti*: A Middle Jurassic sauropod from Peterborough, England," thesis, Gonville and Caius College (2002).

124. J. S. McIntosh, W. P. Coombs Jr., D. A. Russell, A new diplodocid sauropod (Dinosauria) from Wyoming, U.S.A. *J. Vertebr. Paleontol.* **12**, 158–167 (1992).

125. R. D. Martínez, O. d. V. Giménez, J. Rodríguez, M. Luna, M. C. Lamanna, An articulated specimen of the basal titanosaurian (Dinosauria: Sauropoda) *Epachthosaurus sciuttoi* from the early Late Cretaceous Bajo Barreal Formation of Chubut Province, Argentina. *J. Vertebr. Paleontol.* **24**, 107–120 (2004).

126. C. Wiman, Die Kreide-Dinosaurier aus Shantung, *Palaeontol. Sinica (Ser. C)* **6**, 1–67 (1929).

127. E. Tschopp, O. Mateus, Osteology of *Galeamopus pabsti* sp. nov. (Sauropoda: Diplodocidae), with implications for neurocentral closure timing, and the cervico-dorsal transition in diplodocids. *PeerJ* **5**, e3179 (2017).

128. H. You, F. Tang, Z. Luo, A new basal titanosaur (Dinosauria: Sauropoda) from the Early Cretaceous of China. *Acta Geol. Sin.* **77**, 424–429 (2003).

129. X. He, C. Wang, S. Liu, F. Zhou, T. Liu, K. Cai, B. Dai, A new species of sauropod from the Early Jurassic of Gongxian Co., Sichuan. *Acta Geol. Sichuan* **18**, 1–7 (1998).

130. B. R. Erickson, History of the Poison Creek Expeditions 1976–1990, with description of *Haplocanthosaurus* post cranials and a subadult diplodocid skull. *Monogr. Sci. Mus. Minn. (Paleontol.)* **8**, 1–33 (2014).

131. E. Fraas, Ostafrikanische Dinosaurier. *Palaeontographica* **55**, 105–144 (1908).

132. X. Zhao, “The Jurassic Reptilia,” in *The Jurassic System of China*, S.-e. Wang, Z. Cheng, N. Wang, Eds. (Stratigraphy of China, Geological Publishing House, 1985), pp. 286–291.

133. B. J. González Riga, Speeds and stance of titanosaur sauropods: Analysis of *Titanopodus* tracks from the Late Cretaceous of Mendoza, Argentina. *An. Acad. Bras. Cienc.* **83**, 279–290 (2011).

134. C.-C. Young, X. Zhao, *Mamenchisaurus hochuanensis* sp. nov. *Inst. Vertebr. Paleontol. Paleoanthropol. Monogr. Ser. A* **8**, 1–30 (1972).

135. B. J. González Riga, P. D. Mannion, S. F. Poropat, L. D. Ortiz David, J. P. Coria, Osteology of the late cretaceous argentinean sauropod dinosaur *Mendozasaurus neguyelap*: Implications for basal titanosaur relationships. *Zool. J. Linn. Soc.* **184**, 136–181 (2018).

136. R. Royo-Torres, C. Fuentes, M. Meijide Calvo, F. M. Fuentes, M. Meijide Fuentes Jr., A new Brachiosauridae sauropod dinosaur from the lower Cretaceous of Europe (Soria Province, Spain). *Cretac. Res.* **80**, 38–55 (2017).

137. X. He, K. Li, K. Cai, *The Middle Jurassic Dinosaurian Fauna from Dashanpu, Zigong, Sichuan. IV. The Sauropod Dinosaurs 2. Omeisaurus tianfuensis* (Sichuan Scientific and Technological Publishing House, 1988), 143 pp.

138. M. Borsuk-Białynicka, A new camarasaurid sauropod *Opisthocoelicaudia skarzynskii* gen. n., sp. n. from the upper cretaceous of Mongolia. *Palaeontol. Pol.* **37**, 5–64 (1977).

139. P. M. Galton, An early prosauropod dinosaur from the Upper Triassic of Nordwürttemberg West Germany. *Stutt. Beitr. Naturkd. B* **106**, 1–25 (1984).

140. R. P. Ratkevich, New Cretaceous brachiosaurid dinosaur, *Sonorasaurus thompsoni* gen. et sp. nov, from Arizona. *J. Ariz-Nev. Acad. Sci.* **31**, 71–82 (1998).

141. R. Allain, P. Taquet, B. Battail, J. Dejax, P. Richir, M. Veran, F. Limon-Duparcmeur, R. Vacant, O. Mateus, P. Sayarath, B. Khenthavong, S. Phouyavong, Un nouveau genre de dinosaure sauropode de la formation des Gres superieurs (Aptien-Albien) du Laos. *C. R. Hebd. Séances Acad. Sci. Sér. IIA (Sci. Terre Planèt.)* **329**, 609–616 (1999).

142. R. Royo-Torres, P. Upchurch, The cranial anatomy of the sauropod *Turiasaurus riodevensis* and implications for its phylogenetic relationships. *J. Syst. Palaeontol.* **10**, 553–583 (2012).

143. R. Royo-Torres, A. Cobos, L. Alcalá, A giant European dinosaur and a new sauropod clade. *Science* **314**, 1925–1927 (2006).

144. M. A. Raath, Fossil vertebrate studies in Rhodesia: A new dinosaur (Reptilia: Saurischia) from near the Trias-Jurassic boundary. *Arnoldia* **5**, 1–37 (1972).

145. M. Avanzini, G. Leonardi, P. Mietto, *Lavinipes cheminii* ichnogen., ichnosp. nov., a possible sauropodomorph track from the Lower Jurassic of the Italian Alps. *Ichnos* **10**, 179–193 (2003).

146. V. F. Santos, J. J. Moratalla, R. Royo-Torres, New sauropod trackways from the Middle Jurassic of Portugal. *Acta Palaeontol. Pol.* **54**, 409–422 (2009).

147. B. J. González Riga, J. O. Calvo, A new wide-gauge sauropod track site from the Late Cretaceous of Mendoza, Neuquén Basin, Argentina. *Palaeontology* **52**, 631–640 (2009).

148. P. J. Currie, D. Badamgarav, E. B. Koppelhus, The first late cretaceous footprints from the nemegt locality in the Gobi of Mongolia. *Ichnos* **10**, 1–13 (2003).
